# A borindolizine platform for the design of fluorophores with tunable emissions

**DOI:** 10.1039/d5sc04328j

**Published:** 2025-09-15

**Authors:** Chirag N. Apte, Nicholas W. Heller, Ben Zhen Huang, Adam Marr, Kjell Jorner, Alan Aspuru-Guzik, Andrei K. Yudin

**Affiliations:** a Department of Chemistry, University of Toronto Toronto Canada andrei.yudin@utoronto.ca; b Department of Chemistry and Chemical Engineering, Chalmers University of Technology Kemigården 4 Gothenburg SE-41258 Sweden

## Abstract

Here we describe the design and applications of borindolizine, a novel scaffold with broadly tunable fluorescence and a high Stokes shift. Two classes of emitters were synthesized through rational scaffold modification, resulting in blue-emitting carboxyborindolizines (*λ*_max,em_ = 431–459 nm) and green-emitting aryl borindolizines (*λ*_max,em_ = 489–519 nm). Experimental structure-emission trends were used to validate a computational spectral prediction model and to subsequently design a red-emissive borindolizine scaffold. The red-emissive isoquinolyl borinidolizine was prepared, and the experimental emission (*λ*_max,abs_ = 370 nm, *λ*_max,em_ = 635 nm) was in excellent agreement with the theoretical emission (*λ*_max,em_ = 646 nm). These results show how the application of data science can produce fluorophores with desirable spectroscopic properties through the borindolizine scaffold.

## Abstract 2

Here we describe the design and applications of borindolizine, a novel scaffold with broadly tunable fluorescence and a high Stokes shift.

## Graphical abstract

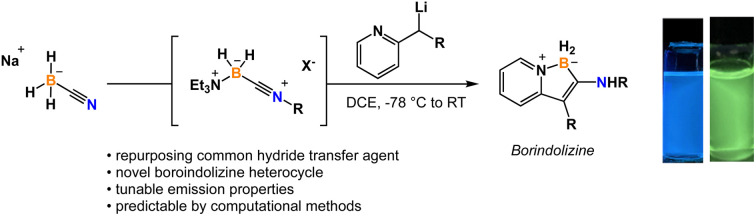

## Introduction

Small molecule fluorescent probes are extensively used in imaging applications in biomedical research,^1–3^ material sciences^4–7^ and drug discovery.^8–10^ The diverse range of applications of small molecule fluorophores necessitates the design of molecules with specific photophysical profiles that are defined by their absorption wavelength (*λ*_max,abs_), emission wavelength (*λ*_max,em_) and quantum yield (*Φ*).^11,12^ The required absorption and emission wavelengths are based on the nature of the application, while higher quantum yields are generally preferred. Imaging applications such as multiplexed fluorescence microscopy benefit from incorporating multiple fluorophores with different emission wavelengths across the ultraviolet to infrared spectrum.^13,14^ Organic chromophores with infrared or near-infrared emissions are particularly attractive for biological imaging due to excellent tissue penetration and consequently better image quality.^15–17^ However, the design of red-emitting fluorophores with low molecular weights poses a significant challenge due to the difficulty of establishing small HOMO–LUMO energy gaps within compact conjugated systems.^18–20^ Control of *λ*_max,abs_ and *λ*_max,em_ values of fluorophores can be effected primarily through structural modification of the chromophore scaffold which is directly influenced by its synthetic tunability. Therefore, the development of novel heterocyclic motifs with a focus on facile and significant structural modularity is instrumental to designing platforms for custom fluorophore synthesis.

Historically, privileged fluorescent motifs such as coumarins, fluoresceins, BODIPY and rhodamines have been extensively studied for wavelength tunability but often suffer from challenging core scaffold modularity and difficulty in achieving *λ*_max,em_ that exceed 600 nm.^21–23^

**Fig. 1:**
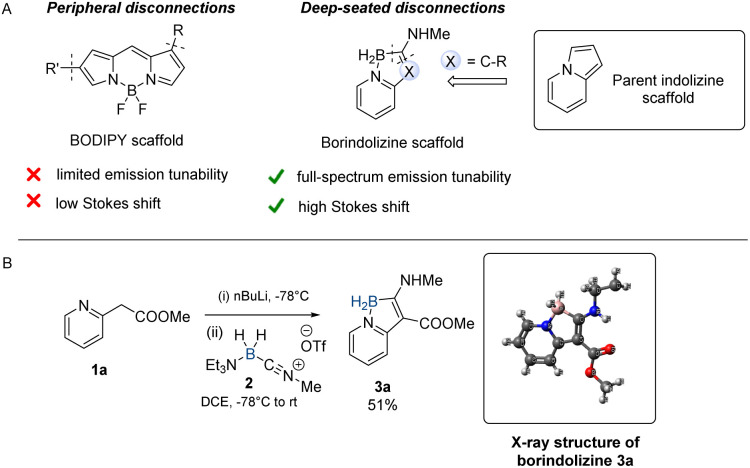
(A) Comparing retrosynthetic strategies of structural modifications of BODIPY and borindolizine. (B) Synthesis of borindolizine 3a.

Synthetically, such tunability is typically achieved through peripheral electronic modifications of the core structure, often resulting in minimal changes in fluorescence emission wavelengths.^3,24^ More recently, novel heterocyclic cores showcasing high synthetic modularity through combinatorial screens have been developed to showcase tunable emission.^24–26^ A notable example of tunable fluorescence by Chenoweth and coworkers was showcased on a quinoline scaffold through a Suzuki-based library synthesis.^26^ While numerous electronic changes were effected through a peripheral disconnection approach (Fig. 1A), only a small degree of emission tunability (90 nm, 482–576 nm, 2.15–2.57 eV) was observed. This underscores the need for versatile synthetic techniques capable of deep-seated scaffold modifications that offer significant modification of photophysical properties.

As part of a research program aimed at functional mimicry of heterocyclic scaffolds through boron isosterism, we recently explored the synthesis of a novel class of boron-containing heterocycles – boramidine – which turned out to exhibit violet-blue fluorescence (378–425 nm, 2.91–3.28 eV, dichloromethane).^27^ Boramidine features a 6,5-fused boron-containing heterocyclic core that is assembled rapidly in a single synthetic step from 2-aminopyridine and *N*-alkyl nitrilium aminoborane 2. Unfortunately, peripheral electronic changes in the boramidine scaffold resulted in minimal changes in the violet-blue emission of the molecule. As such, in this study we envisioned applying our modular boron heterocycle synthesis to the development of a class of fluorescent emitters with high *λ*_max,em_ tunability. The boramidine scaffold is transformed by a N-to-C substitution to afford a boron analog of indolizine – borindolizine (Fig. 1A). In this manner, we access B–N variants of known C–C heterocycles through a deep-seated scaffold modification of the 5-membered boron heterocyclic core to study the impact of structure on the *λ*_max,em_.

## Results and discussion

We hypothesized that the B–N to B–C substitution could be made through the addition of stabilized carbon nucleophiles to *N*-alkylnitrilium cyanoboranes. Alkyl 2-pyridylacetates can be deprotonated using organometallic bases in a facile manner and subsequently alkylated at the benzylic methylene position. Methyl 2-pyridylacetate 1a was deprotonated by *n*-butyllithium at −78 °C, followed by the addition of a solution of nitrilium 2 in dichloroethane and warming to room temperature. Chromatographic purification of the reaction mixture afforded borindolizine 3a in a 51% yield as a highly blue-fluorescent solid (Fig. 1B). Spectroscopic analysis of 3a in dichloromethane showed a blue fluorescent *λ*_max,em_ at 438 nm (*λ*_max,abs_ = 289 nm, 378 nm) with a high Stokes shift (3608 cm^−1^), which is red-shifted from boramidine 1 (R=H) with a *λ*_max,em_ at 392 nm. Borindolizine 3a was crystallized from dichloromethane followed by X-ray analysis which shows a highly planar structure that incorporates an intramolecular hydrogen bond between the aniline-like N–H and the ester C=O (see SI). The compound is bench-stable over an extended period and can be stored under ambient conditions.

**Scheme 1:**
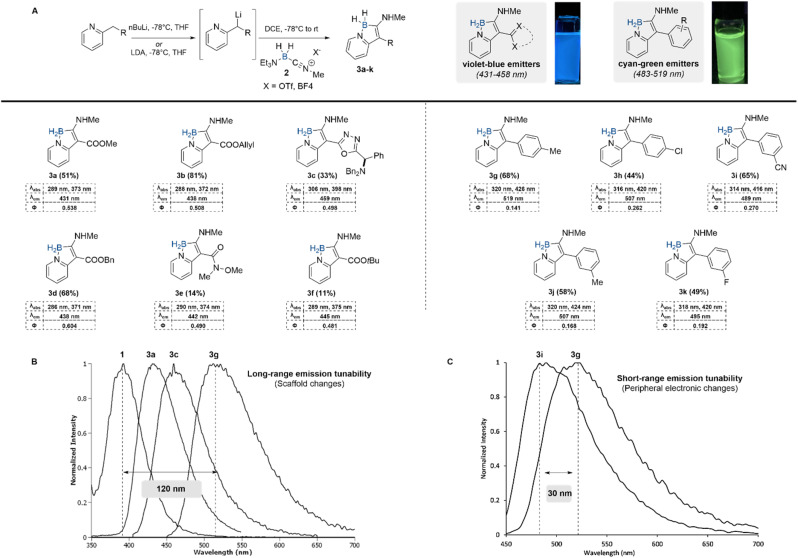
Scope of substituted borindolizine heterocycles synthesis through lithiation–carboxyborylation of 2-methylpyridines. All photophysical data was acquired using dichloromethane as a solvent. (A) General synthesis of borindolizines. (B) Overlayed emission spectra of boron-heterocyclic scaffolds in dichloromethane displaying long range tunability. (C) Overlayed emission spectra of selected aryl borindolizines in dichloromethane displaying short-range tunability.

To explore the chemical as well as the emissive scope of the borindolizine scaffold, a series of substituted 2-methylpyridines were subjected to the standard reaction conditions (Scheme 1). Precursors for borindolizines 1a–g were derived from 2-pyridylacetic acid through standard amide bond-forming conditions. Ester-substituted borindolizines 3a, 3b, 3d, 3e, and 3f were synthesized from their corresponding protected 2-pyridylacetates. Lower yields were observed for Weinreb amide 3e and *tert*-butyl ester 3f due to the limited stability of the lithiated carbanion. Pyridyl 1,3,4-oxadiazole precursor 1c afforded heteroaryl-substituted borindolizine 3c in a 33% yield. The emission wavelengths in dichloromethane of the 2-pyridylacetic acid-derived borindolizines were observed to be between 431–459 nm (violet-blue), wherein the 1,3,4-oxadiazole substituted borindolizine 3c was observed to have the highest emission wavelength (459 nm). Fluorescent quantum yields of the 2-pyridylacetic acid-derived borindolizines were between 0.481–0.604.

To explore the effect that different solvents had on the fluorescent properties of these compounds, we evaluated the photophysical properties of 3a in acetonitrile. In acetonitrile, there was a minimal shift in the absorption wavelengths (*λ*_max,abs_ = 373 nm in dichloromethane, *λ*_max,abs_ = 371 nm in acetonitrile) of 3a. However, the emission wavelength of 3a red-shifted (*λ*_max,em_ = 431 nm in dichloromethane, *λ*_max,em_ = 446 nm in acetonitrile) and the quantum yield dropped (0.538 in dichloromethane, 0.332 in acetonitrile), indicating that solvent polarity could significantly influence both the efficiency and emission wavelength of the fluorophores.

To further evaluate the effect of solvent polarity on photophysical characteristics, we evaluated the photophysical properties of 3b in ethanol. Interestingly, the emission wavelength of 3b also red-shifted in ethanol (*λ*_max,em_ = 438 nm in dichloromethane, *λ*_max,em_ = 442 nm in ethanol) and the quantum yield increased from 0.508 in dichloromethane to 0.607 in ethanol.

With our initial results in hand, we began comparing the electronic properties between our borindolizines and fluorescent indolizines. Similar to our findings, the *λ*_max,em_ of fluorescent indolizines undergo bathochromic shifts as electron density increases at the C3 position.^28–30^ High *λ*_max,em_ are observed in phenyl-substituted indolizines, with increased electron density in the aromatic ring corresponding to a decrease in quantum yield.^31^ A similar trend was observed with borindolizines by switching the substituent at the C3 position from an ester (3d, *λ*_max,em_ = 438 nm, *Φ* = 0.60) to an oxadiazole (3c, *λ*_max,em_ = 459 nm, *Φ* = 0.50). Due to the bathochromic shifts observed in phenyl-substituted indolizines, we decided to evaluate the effect of phenyl substituents on the photophysical properties of the borindolizine scaffold. We hypothesized that phenyl-substituted borindolizines would afford further red-shifted fluorophores.

To access the required pyridyl phenyl precursors, we employed a Barluenga–Valdés cross-coupling between pyridotriazoles and aryl boronic acids as demonstrated by Shen and coworkers^32^ (see SI). Employing our heterocycle forming conditions, a scope of pyridyl phenyls with varying electronics were reacted to form green-emissive borindolizines 3g–k in moderate to good yields (*λ*_max,em_ of 489–519 nm, *Φ* of 0.141–0.270). Gratifyingly, 4-chloro substituted (3h) borindolizine could be synthesized using *n*-butyl lithium in a 44% yield with no observable amounts of dehalogenation side product.

As reported in our earlier work with the synthesis of boramidines,^27^ limited fluorescence tunability was observed with changes in the substitution of the pyridine ring. In our scope of borindolizines, we have demonstrated rapid scaffold modifications from enaminoester-based scaffolds (3a–f) to biaryl scaffolds (3g–k) under the same synthetic regime. Consequently, we observed long-range emission tunability in wavelengths between the boramidine (397 nm, 3.12 eV), ester (3a, 431 nm, 2.87 eV), heteroaryl (3c, 458 nm, 2.71 eV), and 4-tolyl (3g, 519 nm, 2.39 eV) substituted borindolizines – effectively spanning a 120 nm range from violet to the green regions (Scheme 1A). Furthermore, changing the electronics of the pendant aryl group through substituent changes results in a smaller change in emission wavelengths, offering a handle for fine-tuning of emission (Scheme 1B).

**Fig. 2:**
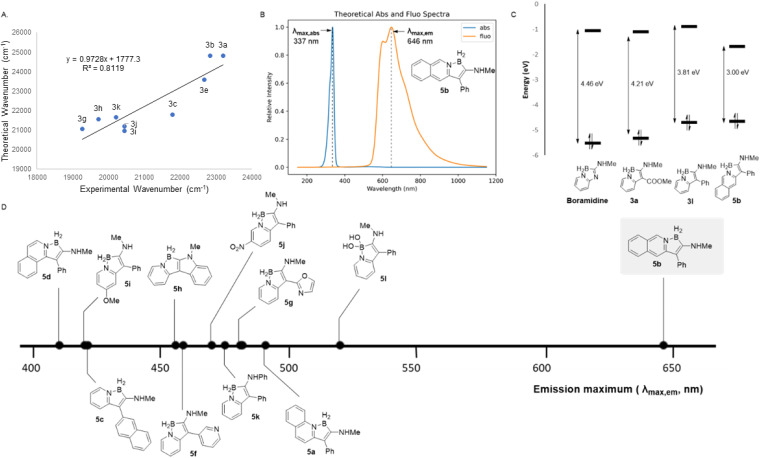
(A) Correlation between theoretical and experimental emission peak maxima (wavenumber, cm^−1^). (B) Predicted absorbance and emission spectra of 5b. (C) Comparison of HOMO–LUMO energy gaps of boramidine and borindolizine scaffolds. (D) Trends in theoretical emission wavelengths of simulated compounds 5a–l.

Electronic variability of the heterocyclic scaffold affords violet emissive boramidines 1, blue-emissive carboxy-borindolizines (3a–f) and green-emissive biaryl borindolizines (3g–k). We envisioned that this scaffold hopping approach could be extended to rationally design red-emissive fluorophores resulting in a library of Stokes-shifted emitters that accesses violet, blue, green and red wavelengths. To this end, the absorption and emission spectra of all synthesized heterocycles were investigated computationally (see SI). Spectral simulations reproduce all qualitative features of the emission and absorption spectra (Fig. 2A).

While simulated spectra were observed to be blue-shifted relative to experimental spectra, a moderate correlation (*R*^2^ = 0.81) was observed between the corresponding absorption and emission maxima wavenumber values (Fig. 2A). Motivated by the agreement between experimental and computational data, we modelled the absorbance and fluorescence emission spectra for 12 structures (5a–l) containing a range of structural modifications (SI) and a general trend between fluorophore structure and spectral properties was established. Amongst a variety of structural variations, the effects of extension of conjugation on the emission were investigated through several benzannulated (5a–d) modifications of the borindolizine scaffold (Fig. 2C). Promisingly, isoquinoline-based borindolizine 5b was predicted to be a red-emissive fluorophore with a *λ*_max,em_ of 646 nm (Fig. 2B). In addition to spectral simulations, HOMO–LUMO energy gaps were calculated (B3LYP/6-31G**) for all simulated molecules. Lower-level and computationally inexpensive ground state DFT calculations offered compelling qualitative insights into the effects of scaffold changes on the frontier molecular orbitals of the heterocyclic scaffold (Fig. 2D). From a frontier molecular orbital perspective, there are two major strategies to reduce the HOMO–LUMO gap (HLG) – extension of conjugation (LUMO lowering effect) and changing the electron density of the conjugated system.^20^ The extension of conjugation on the pyridine ring system leading to the isoquinolyl scaffold 5b results in a LUMO lowering effect, reducing the band gap energy to 3.00 eV. Overall, the large change in band gap energy across the scope of molecules investigated (1.46 eV) is a strong indication of the dramatic changes in the intrinsic electronics of the borindolizine scaffold.

**Fig. 3:**
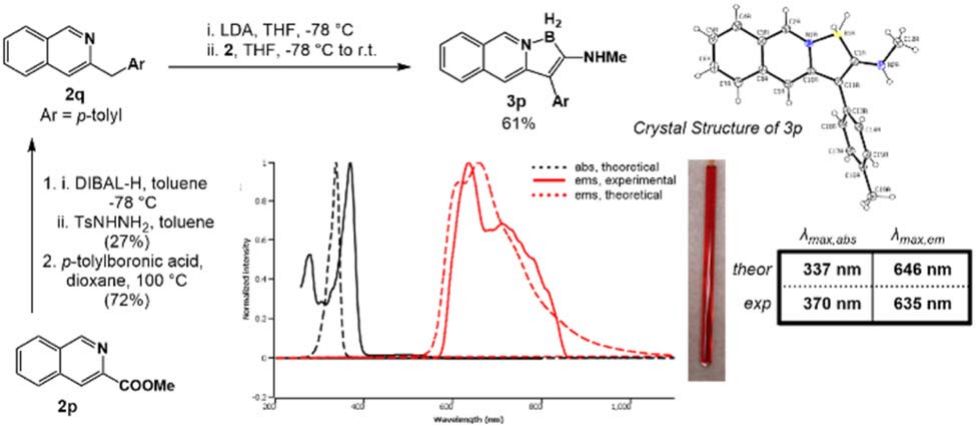
Synthesis and photophysical characterization of iso-quinolyl borindolizine 3p.

Guided by the above computational screen, isoquinoline precursor 2q was synthesized through reduction and subsequent Barluenga–Valdés cross-coupling^33^ of methyl isoquinoline-3-carboxylate 2p with *p*-tolylboronic acid (Fig. 3A). Employing the standard borylative procedure, isoquinolyl borindolizine 3p was isolated as a red solid in a 68% yield. Satisfyingly, the compound was red-emissive in dichloromethane with a *λ*_max,em_ of 635 nm and a broad emission between 610 and 840 nm in excellent agreement with the predicted emission value of 646 nm. There is a forbidden transition in the region ∼480–500 nm, which is validated by the calculations as the S_0_ → S_1_ transition with a very low oscillator strength (calculated at 0.006). This result validates the capacity of the excited-state computations to effectively predict the absorption and emission wavelengths of the borindolizine class of fluorophores, allowing for the rational design and photophysical tunability of these fluorophores.

## Conclusions

This work highlights the enabling aspects of borylated building blocks such as borane nitrilium to rapidly access structurally diverse boron heterocycles with useful photophysical properties. The borindolizine scaffold is constructed in a single synthetic step from readily accessible substituted 2-methylpyridines. This deep-seated disconnection allows for significant changes in the scaffold as well as in the fluorescence emission wavelengths resulting in two new classes of emitters – violet-blue (carboxy-borindolizines) and cyan-green (aryl borindolizines) with high Stokes shifts. Subsequently, the experimental emission values were used to validate a computational model for predicting photophysical spectra. Through this model, a new class of small molecule red-emitters was developed through a synthetically validated approach. Altogether, borindolizines represent a boron-containing heterocyclic platform for the synthesis of tunable fluorophores and should serve as an inspiration for unique approaches to targeted chromophore design.

## Author contributions

C. A. and A. K. Y. conceived the project. C. A., N. W. H. and A. K. Y. wrote the manuscript. A. K. Y. and A. A. G. supervised the project. C. A. and N. W. H. designed and conducted the synthesis of the compounds and carried out spectroscopic studies. A. M. and K. J. conducted the computational investigation. All authors have given approval to the final version of the manuscript.

## Conflicts of interest

A. K. Y. is an associate editor of *Chemical Science*.

## Supplementary Material

SC-016-D5SC04328J-s001

## Data Availability

The data supporting this article have been included as part of the SI. See DOI: https://doi.org/10.1039/d5sc04328j.
