# Supplementary material for: A borindolizine platform for the design of fluorophores with tunable emissions
Source: Chem Sci. 2025 Sep 15;16(42):19786–91. doi: 10.1039/d5sc04328j (PMC12461217; doi:10.1039/d5sc04328j)
Supplement: SC-016-D5SC04328J-s001 [file SC-016-D5SC04328J-s001.pdf]

Supplementary Information (SI) for Chemical Science.
This journal is © The Royal Society of Chemistry 2025

A borindolizine platform for the design of fluorophores with tunable emissions

Chirag N. Apte, Nicholas W. Heller, Ben Zhen Huang, Adam Marr, Kjell Jorner, Alan Aspuru-Guzik, Andrei K. Yudin*

Supporting Information

Contents

Synthesis of substituted methyl 2-pyridyl precursors 3

General procedure for synthesis of boron heterocycles.................... 8

Summarized photophysical data of synthesized compounds 3a – 3p 34

Quantum Yield Measurements.................... 36

Computational Study: Spectral simulations.................... 38

Crystallographic data.................... 50

General: All solvents were of reagent grade quality and were not dried using dessicants unless mentioned otherwise. All reagents were purchased from commercial sources and used as received.

Chromatography: Manual or automated flash column chromatography was carried out using Silicycle 230- 400 mesh silica gel and Brockman I 150 mesh basic alumina. Thin-layer chromatography (TLC) was performed on Macherey Nagel pre-coated glass backed TLC plates (SIL G/UV254, 0.25 mm) and visualized using a UV lamp (254 nm and 365 nm), KMnO4 or curcumin stain.

Nuclear Magnetic Resonance Spectroscopy: 1H NMR, 13C, 11B and 19F spectra were recorded on Varian Mercury 300 MHz, 400 MHz, 500 MHz, 600 MHz or 700 MHz spectrometers1 H NMR spectra chemical shifts (δ) are reported in parts per million (ppm) referenced to residual protonated solvent peak (CDCl3 δ = 7.26). Spectral data is reported as follows: chemical shift, multiplicity (s = singlet, d = doublet, t = triplet, q = quartet, dd = doublet of doublets, dt = doublet of triplets, ddt = doublet of doublet of triplets, dtd = doublet of triplet of doublets, m = multiplet, br = broad), coupling constant (J) in Hertz (Hz), and integration. 13C NMR spectra chemical shifts (δ) are reported in parts per million (ppm) were referenced to carbon resonances in the NMR solvent (CDCl3 δ = 77.2; center line).

Mass Spectroscopy: High resolution mass spectra were obtained on a VG 70- 250S (double focusing) mass spectrometer at 70 eV or on an ABI/Sciex Qstar mass spectrometer with DART source, MS/MS and

accurate mass capabilities. RP-HPLC/MS: Low-resolution mass spectra (ESI) were collected on an Agilent Technologies 1200 series HPLC paired to a 6130 Mass Spectrometer. Compounds were resolved on Phenomenex's Kinetex 2.6u C18 50x4.6mm column at room temperature with a flow of 1 mL/min. The gradient consisted of eluents A (0.1% formic acid in double distilled water) and B (0.1% formic acid in HPLC-grade acetonitrile). Method A: A linear gradient starting from 5% of B to 95% over 15 min at a flow rate of 1.0 mL/min. Method B: A linear gradient starting from 5% of B to 95% over 4 min at a flow rate of 1.0 mL/min. Stays constant at 95% for 1 min and then returns to 5% over 0.5 min. Method C: Stays constant at 5% of B for 0.5 min at a flow rate of 1.0 mL/min, followed by a linear gradient to 95% over 5.5 min. Stays constant at 95% of B for 0.5 min and then returns to 5% B over 0.5 min.

<u>Synthesis of Substituted Methyl 2-Pyridyl Precursors</u>

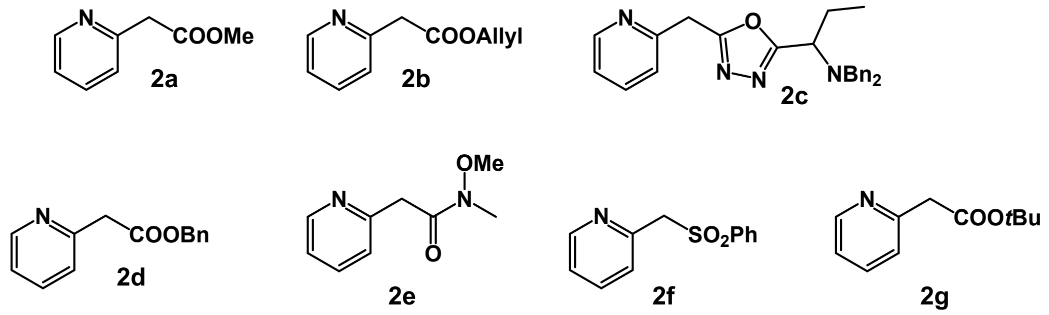

Figure S1: 2-pyridyl ester precursors to carboxyborindolizines (2a - 2g)

Protected 2-pyridyl esters **2a**, **2b**, **2d**, **2e** and **2g** were synthesized by standard amide and ester bond-forming procedures as detailed by Hartwig *et. al.* [1]. 1H and 13C spectra of the synthesized esters matched literature values and were used for subsequent operations. Sulfone **2f** was synthesized from sodium phenylsulfinate and 2-(chloromethyl)pyridine based on the procedure by Masson *et. al.* [2] Oxadiazole **2c** was synthesized based on an adapted procedure of the multicomponent reaction based on N-(isocyanoimino)triphenylphosphorane.[3]

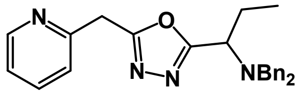

Oxadiazole 2c: 2-Pyridylacetic acid (87 mg, 0.5 mmol) was dissolved in dichloromethane (2.5 mL) followed by propionaldehyde (54 µL, 0.75 mmol) and dibenzylamine (144 µL, 0.75 mmol). Then, (N-isocyaoimino)triphenylphosphorane (167 mg, 0.55 mmol) was added under stirring at room temperature, and the reaction mixture was refluxed for 16 hours. The solvent was then removed under vacuum and the resulting residue was purified by reverse-phase chromatography (30% MeCN/H2O to 100% MeCN/H2O, 0.1% Formic Acid) to afford the desired product after lyophilization (34.5%), brown liquid; ^{1}H NMR (500 MHz, $CDCl_3$) δ 8.61 (m, 1H), 7.71 (t, J = 7.7 Hz, 1H), 7.33 (m, 10H), 7.24 (m, 2H), 4.48 (s, 2H), 3.90 (m, 3H), 3.27 (d, J = 13.7 Hz, 2H), 2.00 (m, 2H), 0.95 (t, J = 7.3 Hz, 3H). ^{13}C NMR (126 MHz, $CDCl_3$) δ 166.4, 164.3, 154.2, 149.6, 139.0, 137.1, 128.8, 128.2, 127.0, 123.2, 122.5, 55.7, 54.2, 34.5, 23.1, 11.0.

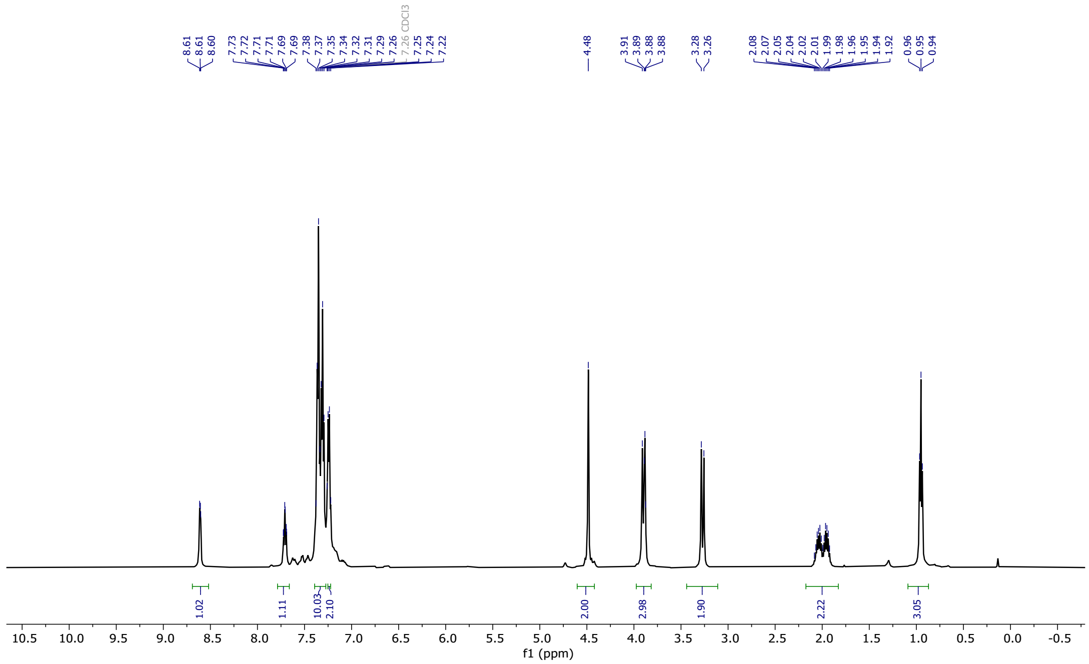

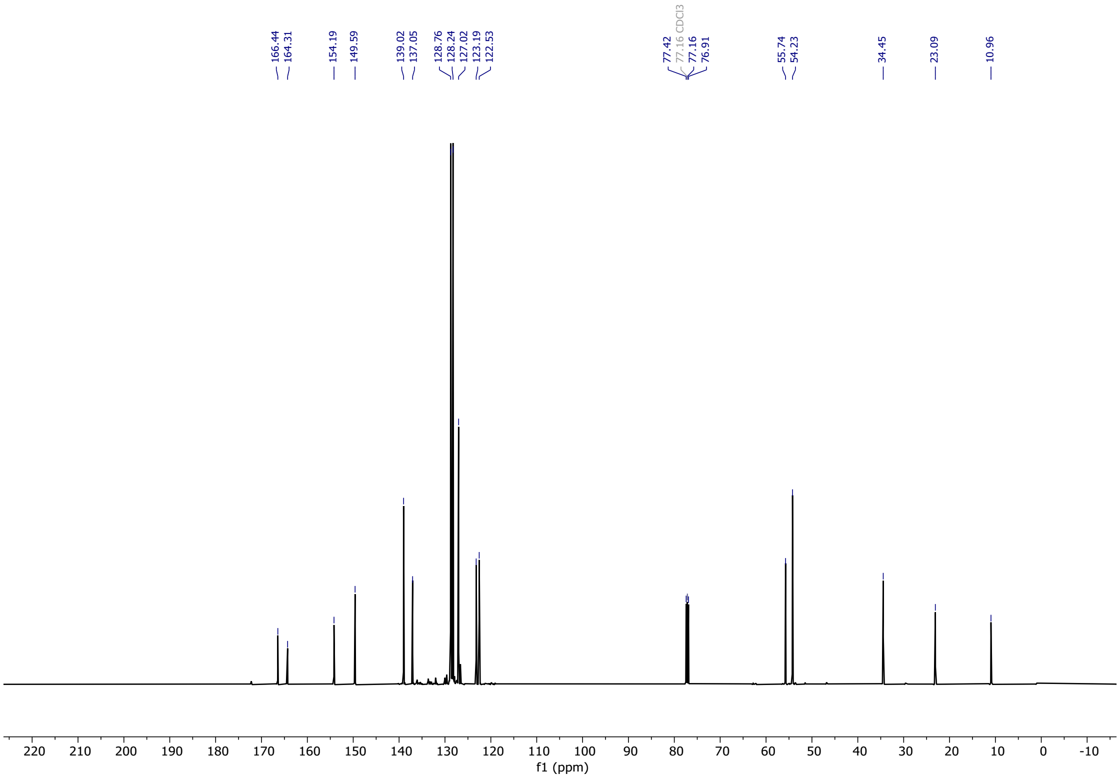

Figure S2: ^{1}H and ^{13}C NMR of oxadiazole **2c** in $CDCl_3$ (298K)

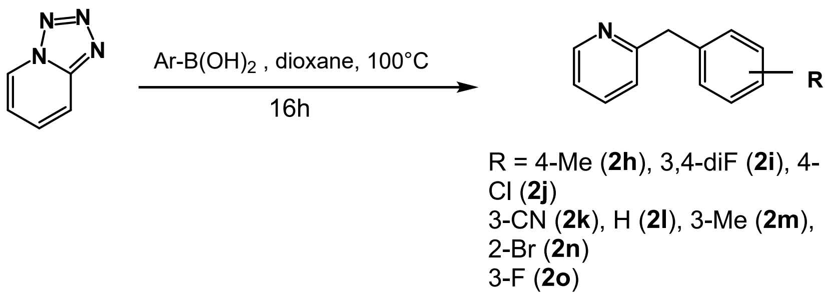

Scheme S1: Synthesis of pyridylaryls 2h - 2o

2-pyridyl aryl subtrates **2h** – **2o** were synthesized based on the protocol by Shen *et. al.* from their corresponding boronic acids.[4] Spectra of all synthesized compounds matched literature precedent.

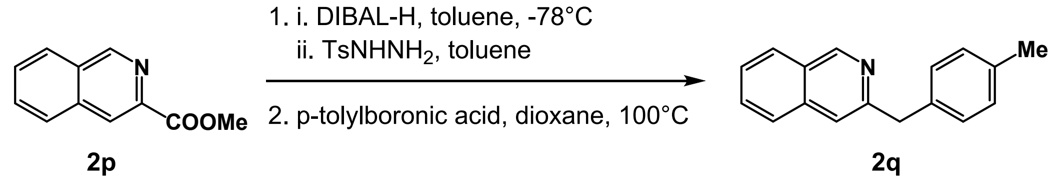

Scheme S2: Synthesis of isoquinolylaryl 2q from methyl 3-isoquinoline carboxylate

A flame-dried 25 mL RBF was charged with a solution of methyl 3-isoquinolinecarboxylate (**2p**, 384 mg, 2.1 mmol) in dry toluene (13 mL) and cooled to -78°C. A solution of DIBAL-H in toluene (1.5M, 2.34 mL, 3.4 mmol) was added dropwise over the course of 30 mins. The reaction was stirred for 3 hours and , warmed up to 0°C and diluted with diethyl ether (dry, 20 mL). Deionized water (0.15 mL), 15% aqueous sodium hydroxide (0.15 mL) and deionized water (0.4 mL) were added dropwise, sequentially, followed by magnesium sulfate. The suspension was warmed up to room temperature, filtered, concentrated and resolubilized in methanol (10 mL). Tosylhydrazide (430 mg, 2.3 mmol) was added in a single portion and stirred for 5 mins and complete conversion to the corresponding tosylhydrazone **2p'** was observed (Rf = 0.25, 7:3 ethyl acetate:hexanes). The tosylhydrazone intermediate was purified by flash column chromatography in a 27% yield (182 mg, 0.56 mmol). ^{1}H NMR (500 MHz, $CDCl_3$) δ 9.26 (s, 1H), 8.06 (d, J = 0.9 Hz, 1H), 7.90 (m, 3H), 7.79 (ddd, J = 8.2, 6.9, 1.3 Hz, 1H), 7.74 (m, 2H), 7.41 (s, 1H), 7.30 (d, J = 8.0 Hz, 2H), 2.40 (s, 3H). ^{13}C NMR (126 MHz, $cdcl_3$) δ 151.3, 146.3, 143.7, 137.9, 136.7, 136.0, 131.9, 129.7, 129.3, 128.2, 128.1, 127.8, 127.5, 123.2, 21.7.

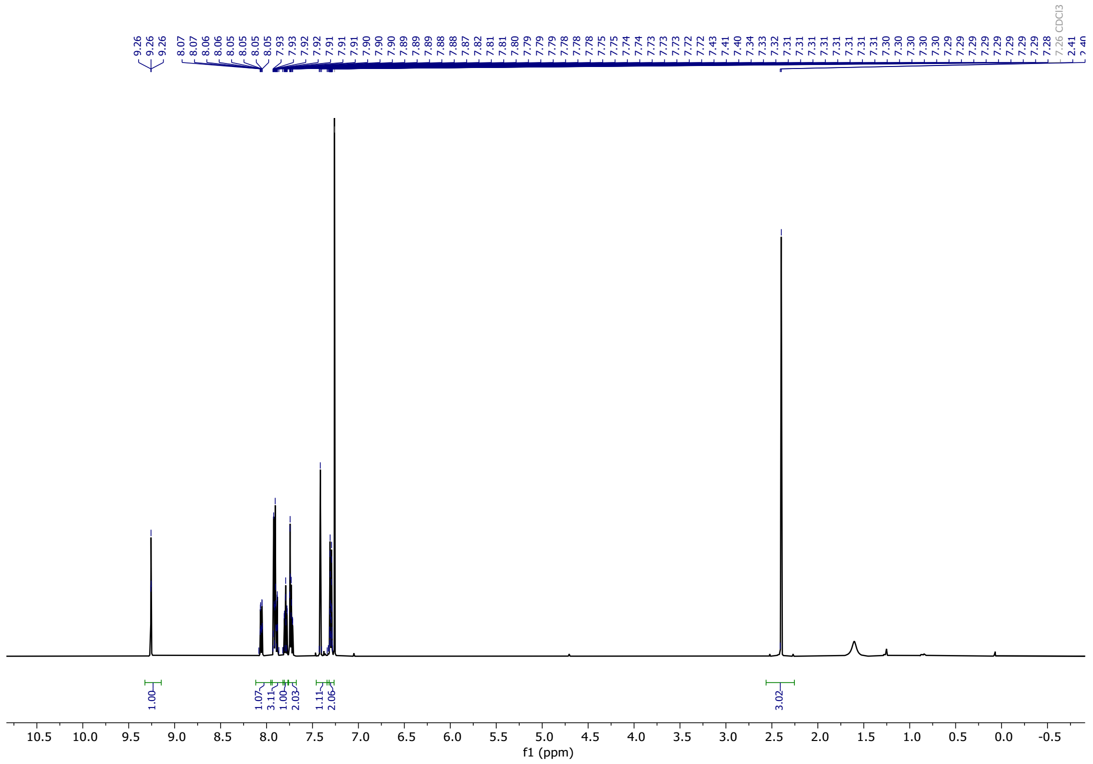

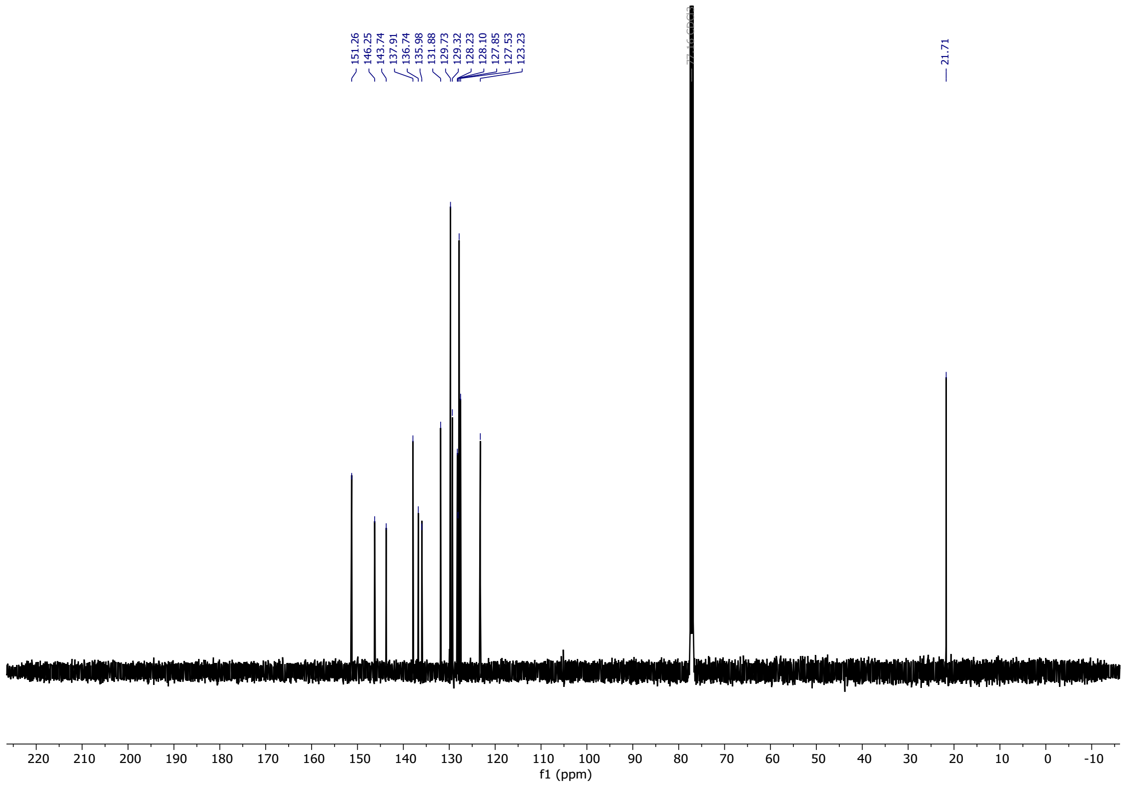

Figure S3: ^{1}H and ^{13}C NMR of tosylhydrazone **2p'** in $CDCl_3$ (298K)

To a flame-dried 10 mL scintillation vial, tosylhydrazone **2p’** (58 mg, 0.18 mmol, 1 equiv.), 4-methylphenyl boronic acid (73 mg, 0.54 mmol, 3 equiv.) and dioxane (1.8 mL) were added and heated up to 100°C for 16 hours. The crude biaryl **2q** was purified by flash column chromatography (20% ethyl acetate in hexanes) to afford pure **2q** in a 72% yield (30 mg) as a pale yellow solid. ^{1}H NMR (500 MHz, $CDCl_3$) δ 9.22 (s, 1H), 7.93 (d, *J* = 8.2 Hz, 1H), 7.71 (d, *J* = 8.3 Hz, 1H), 7.63 (ddd, *J* = 8.2, 6.8, 1.2 Hz, 1H), 7.53 (ddd, *J* = 8.1, 6.8, 1.2 Hz, 1H), 7.43 (s, 1H), 7.22 (d, *J* = 7.9 Hz, 2H), 7.14 (d, *J* = 7.7 Hz, 2H), 4.29 (s, 2H), 2.33 (s, 3H). ^{13}C NMR (126 MHz, $CDCl_3$) δ 154.8, 152.4, 136.8, 136.7, 136.0, 130.5, 129.4, 129.2, 127.6, 127.2, 126.7, 126.4, 118.7, 44.0, 21.2.

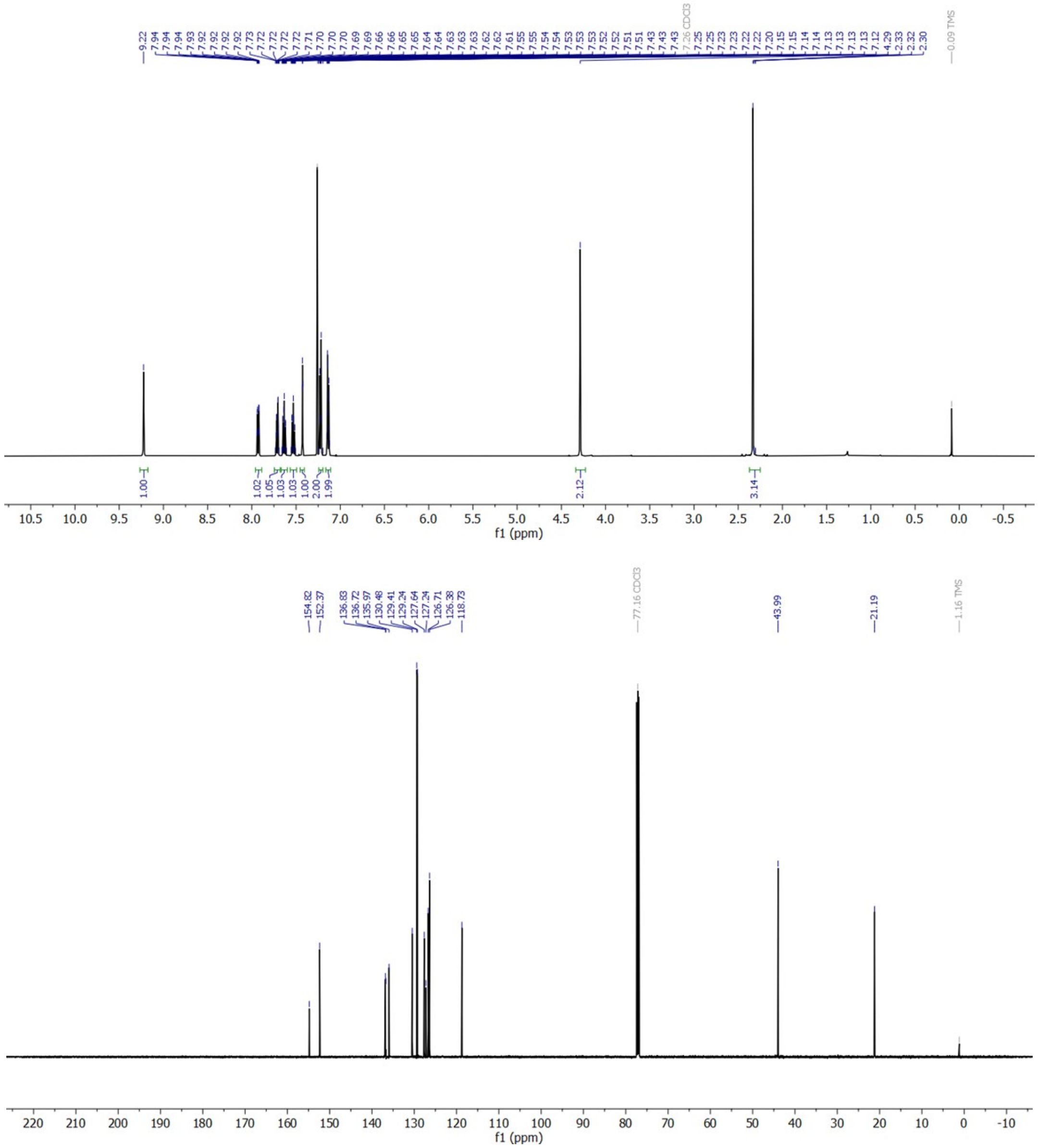

Figure S4: ^{1}H and ^{13}C NMR of isoquinolylaryl **2a** in $CDCl_3$ (298K)

General procedure for synthesis of N-alkylnitriuliumborane

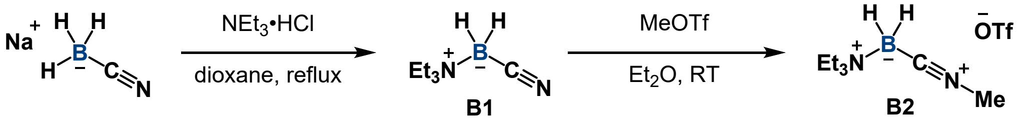

N-methylnitriliumborane was synthesized based on the protocol by Lebedev *et. al.* from sodium cyanoborohydride.[5] Spectra of all synthesized compounds matched literature precedent.

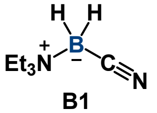

B1

Triethylamine cyanoborane (B1): 62%, colorless oil. ^{1}H NMR (500 MHz, $CDCl_3$) δ 2.90 (q, J = 7.3 Hz, 6H), 1.22 (t, J = 7.3 Hz, 9H). ^{13}C NMR (126 MHz, $CDCl_3$) δ 51.8, 47.1, 8.9, 8.5.

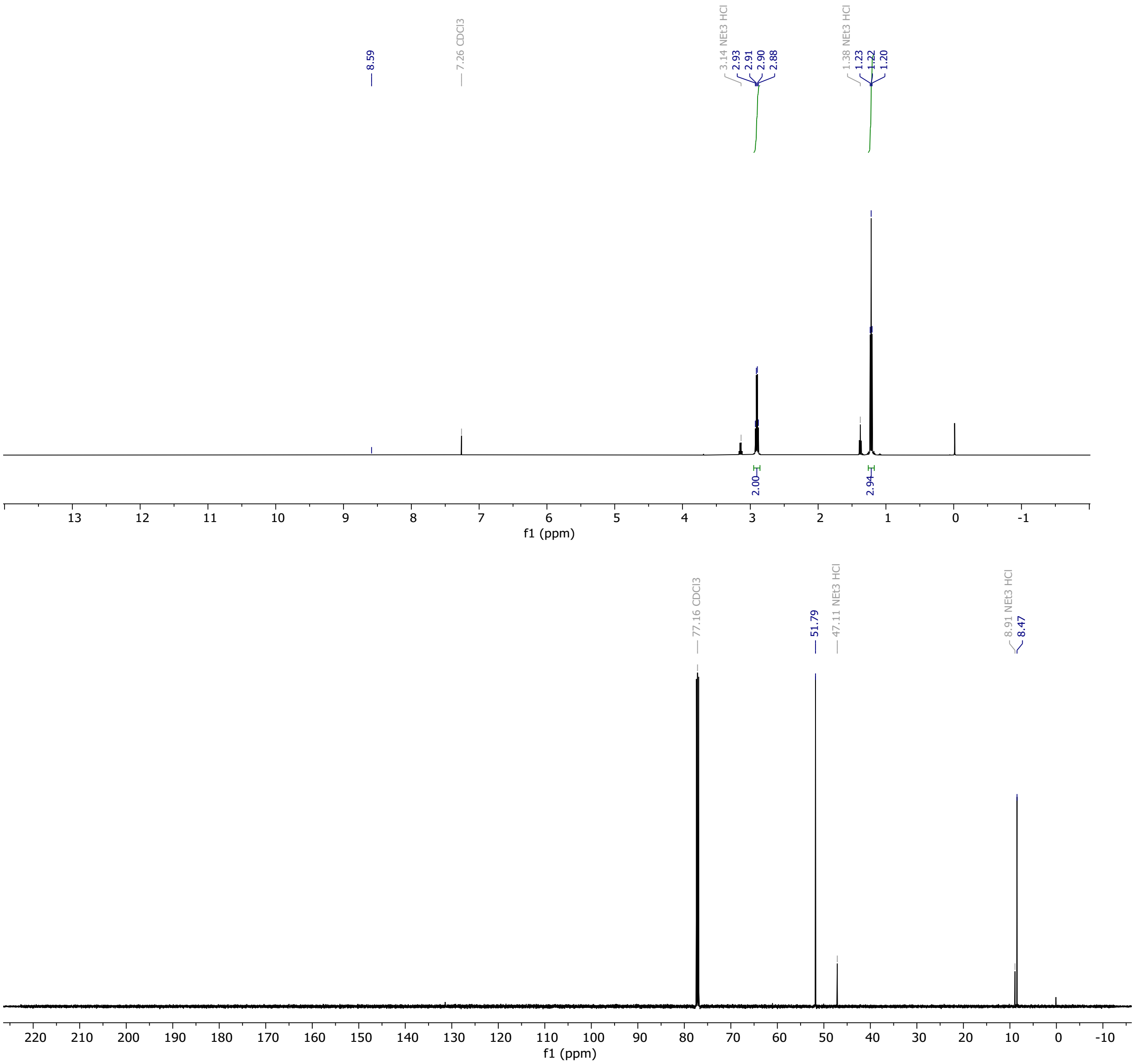

Figure S5: ^{1}H and ^{13}C NMR of triethylamine cyanoborane **B1** in $CDCl_3$ (298K)

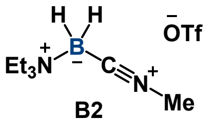

B2

N-methylnitriliumborane (B2): 95%, white solid. ^{1}H NMR (500 MHz, $CDCl_3$) δ 3.83 (t, *J* = 2.0 Hz, 3H), 3.04 (q, *J* = 7.2 Hz, 7H), 1.23 (t, *J* = 7.3 Hz, 12H). ^{13}C NMR (126 MHz, $CDCl_3$) δ 120.85 (q, *J* = 320.2 Hz), 53.03, 31.15, 8.42. ^{11}B NMR (128 MHz, $CDCl_3$) δ 19.10.

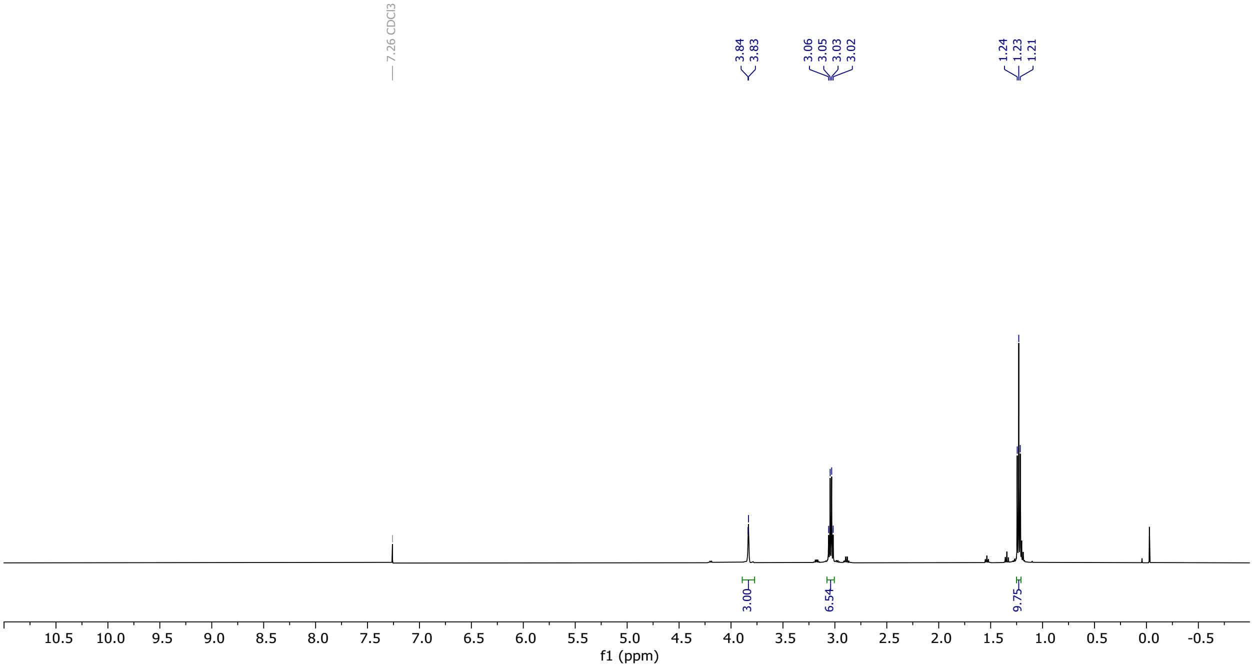

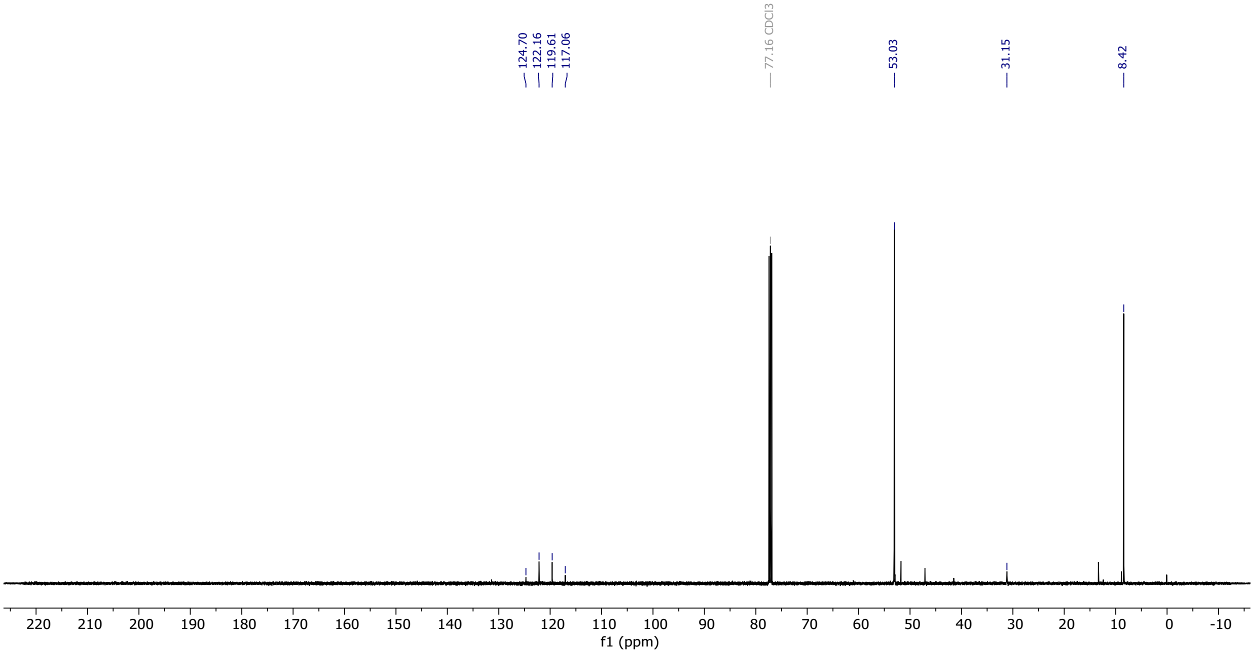

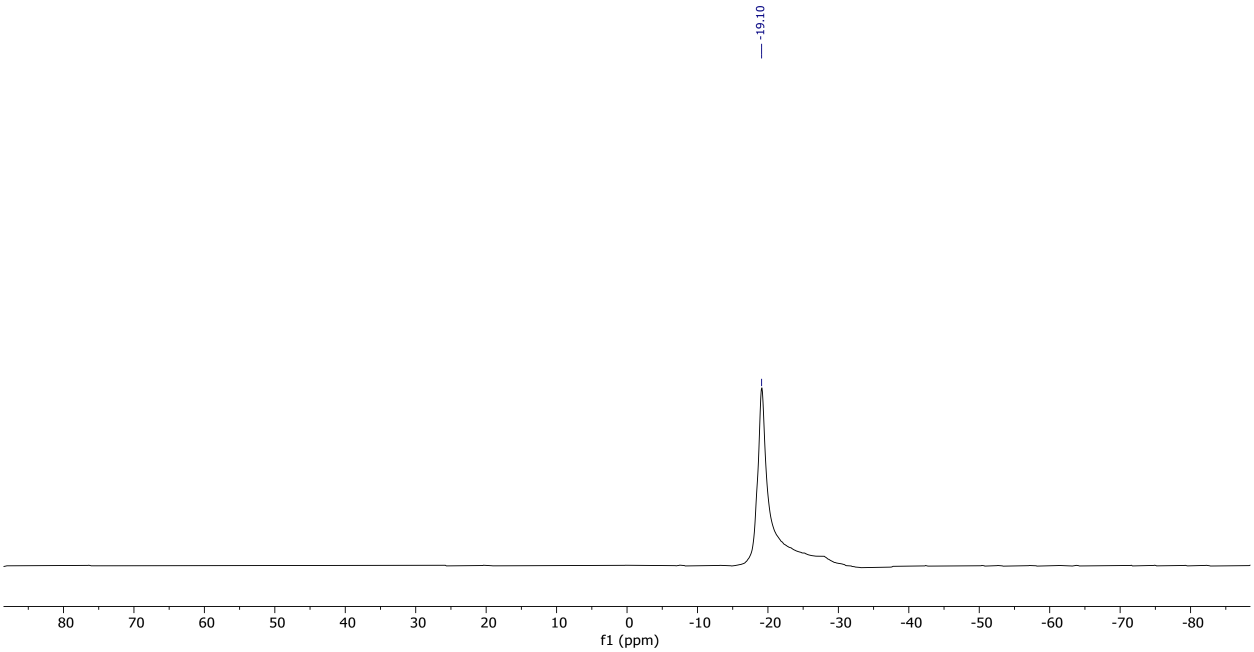

Figure S6: ^{1}H, ^{13}C and ^{11}B NMR of N-methylnitriliumborane **B2** in $CDCl_3$ (298K)

<u>**General procedure for synthesis of boron heterocycles**</u>

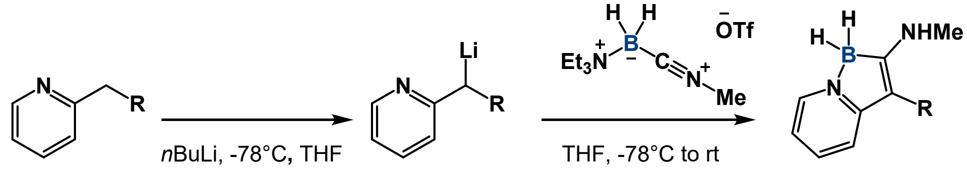

To a flame-dried 20 mL scintillation vial equipped with a magnetic stir bar and sealed with a rubber septum under nitrogen was added the pyridyl substrate (0.25 mmol) in THF (2.5 mL) and was cooled down to -78°C in a dry ice/acetone bath. *n*BuLi (2.5M in hexanes, 0.275 mmol, 1.1 eq) was added dropwise to the reaction over a period of 2 minutes, and the reaction was stirred for 10 minutes at -78°C and then was allowed to warm up to room temperature. A solution of nitrilium (84 mg, 0.275 mmol, 1.1 eq) in THF (1 mL) was added to the reaction in a single portion and was allowed to stir for 12h at room temperature. Celite was added to the reaction and the solvent was removed *in vacuo*. The crude residue was then purified by flash column chromatography (SiO_2) using ethyl acetate/hexanes (non-aryl substituted) or dichloromethane/methanol (Al_2O_3) (aryl substituted).

Note: for each compound, the carbon atom ipso to the $-BH_2$ was not detectable in ^{13}C NMR spectra due to splitting caused by the ^{11}B nucleus.

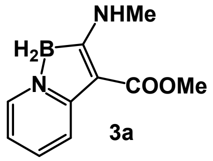

3a

Carboxyborindolizine 3a: 51% yield, orange solid.^{1}H NMR (400 MHz, $CDCl_3$) δ 8.02 (d, J = 5.9 Hz, 1H), 7.86 (s, 1H), 7.56 (t, J = 7.8 Hz, 1H), 6.73 (t, J = 1.2 Hz, 1H), 3.82 (s, 3H), 3.06 (d, J = 5.0 Hz, 3H). ^{13}C NMR (126 MHz, $CDCl_3$) δ 167.0, 160.8, 141.9, 138.3, 118.5, 114.3, 95.1, 50.2, 34.5. ^{11}B NMR (128 MHz, $CDCl_3$) δ -12.28 (t, J = 100.4 Hz). HRMS m/z (ESI) calcd for $C_{10}H_{14}BN_2O_2$ ([M] + H) 205.11428 found 205.11407.

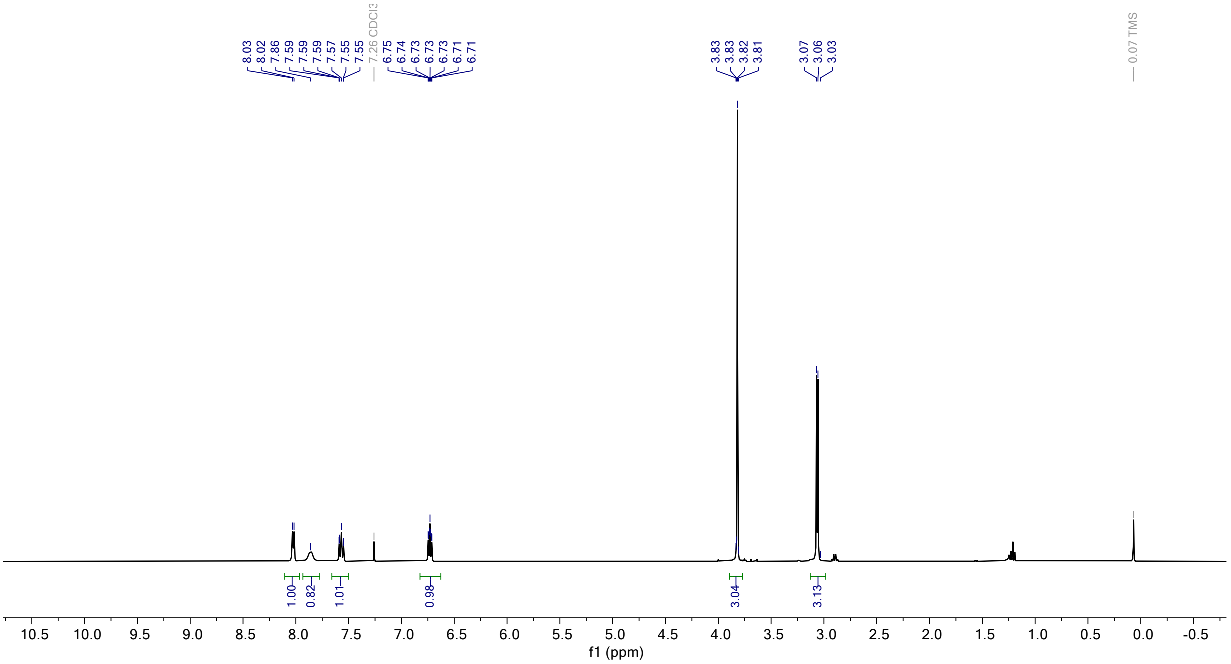

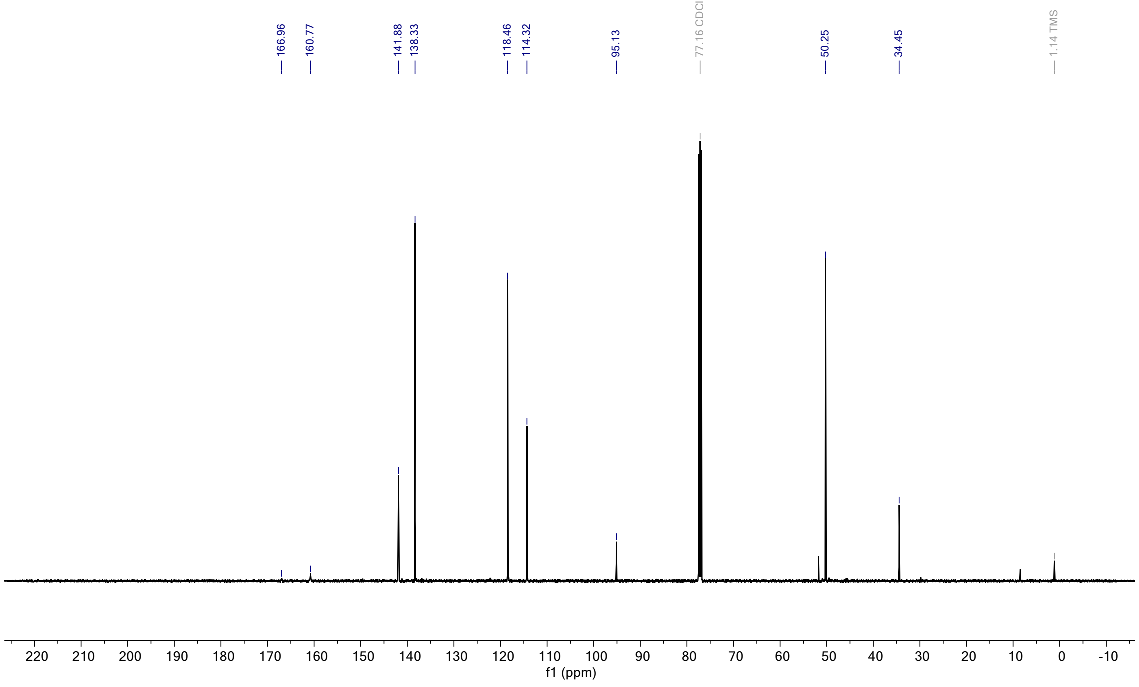

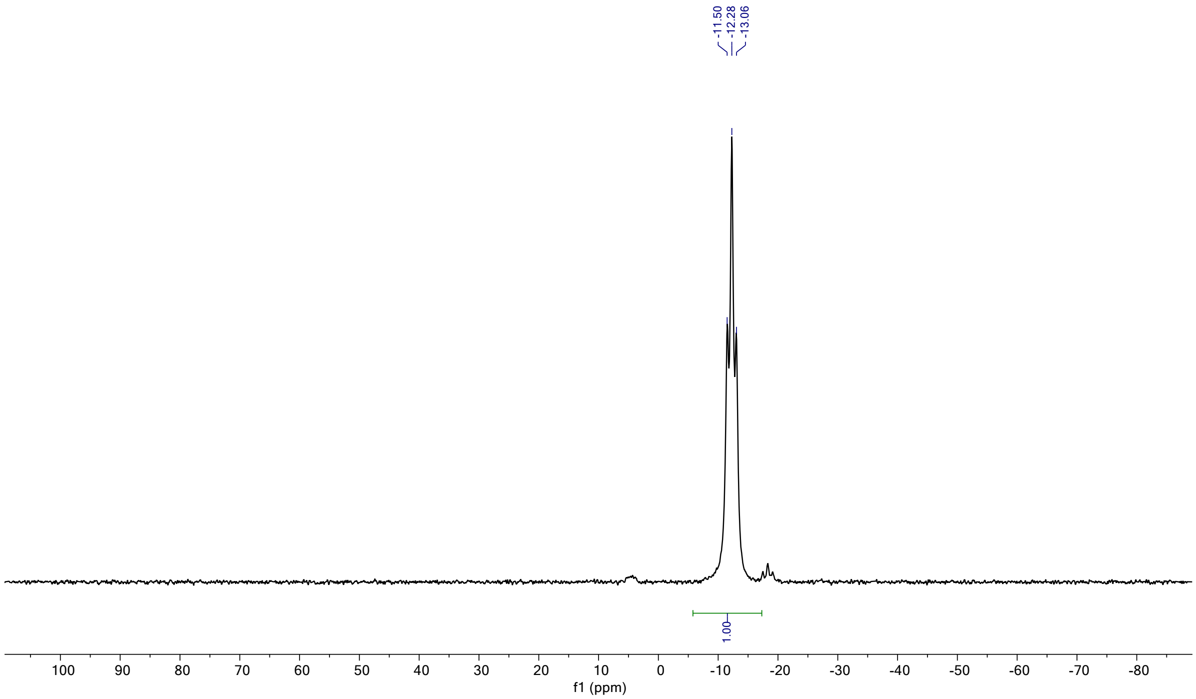

Figure S7: ^{1}H, ^{13}C and ^{11}B NMR of carboxyborindolizine **3a** in $CDCl_3$ (298K)

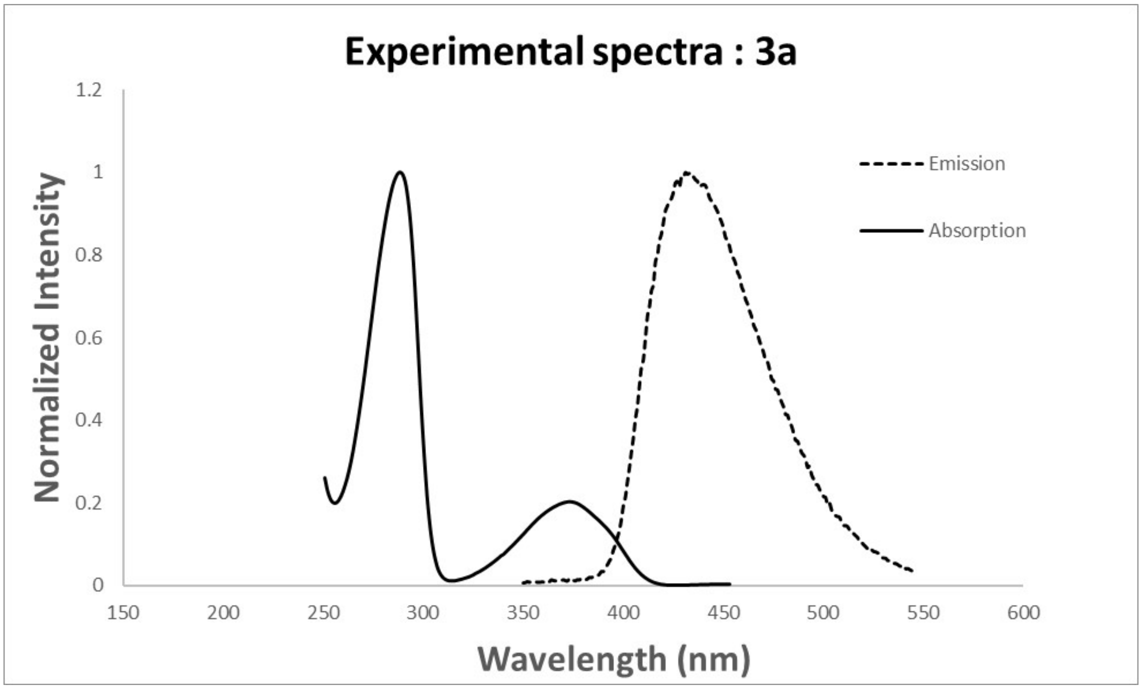

Figure S8: Overlayed absorption and emission spectra of carboxyborindolizine **3a** in dichloromethane.

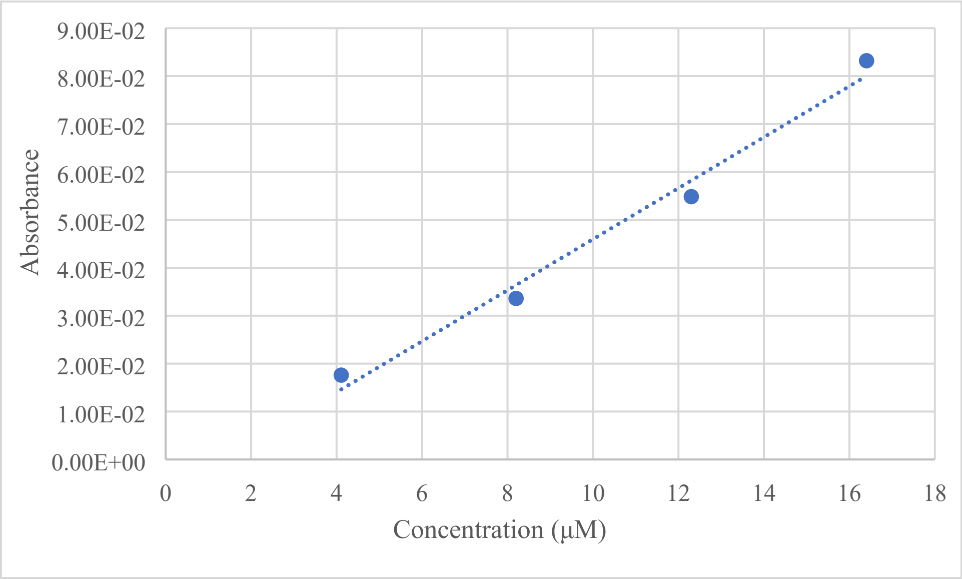

Figure 9: Molar absorptivity of carboxyborindolizine **3a** in dichloromethane (λ= 373 nm, ε = 5.3 × 10^3 $M^{-1}cm^{-1}$)

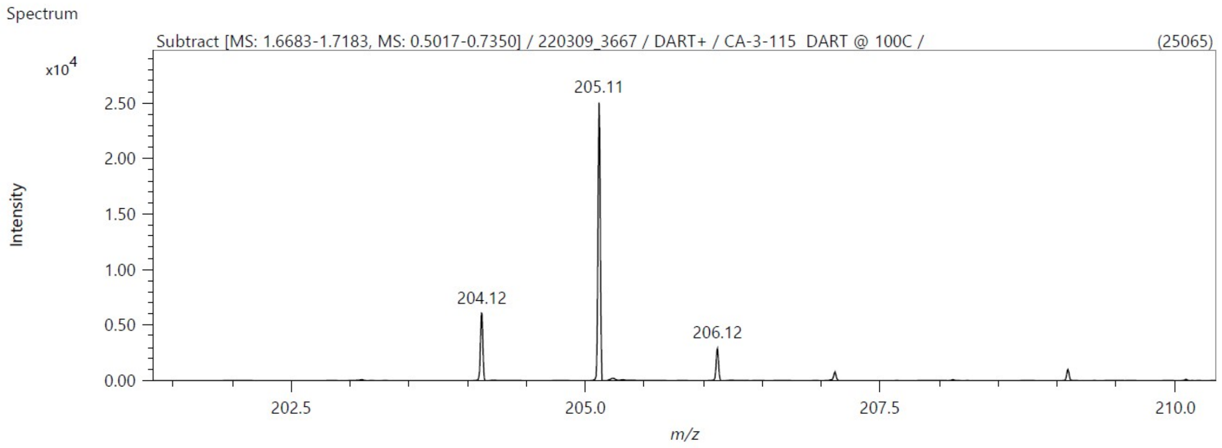

Elemental Composition

Parameters	
Tolerance:	±10.00 mDa
Electron:	Odd/Even
Charge:	+1
DBE:	-1.5 - 100.0

Elements Set 1:

Symbol	C	H	O	N	B
Min	0	0	0	0	1
Max	100	100	20	10	1

Results

Mass	Intensity	Formula	Calculated Mass	Mass Difference [mDa]	Mass Difference [ppm]	DBE
205.11407	25065.11	C10 H14 B N2 O2	205.11428	-0.22	-1.06	5.5
		C8 H12 B N5 O	205.11294	1.13	5.48	6.0
		C7 H16 B N O5	205.11160	2.46	12.01	1.0
		C6 H10 B N8	205.11160	2.47	12.03	6.5
		C5 H14 B N4 O4	205.11026	3.81	18.55	1.5
		C15 H14 B	205.11831	-4.24	-20.67	9.5

Figure S10: HRMS of carboxyborindolizine **3a** (DART+)

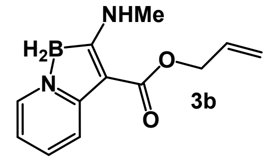

3b

Carboxyborindolizine 3b:: Yield: 81%, yellow solid; ^{1}H NMR (500 MHz, $CDCl_3$) δ 8.01 (d, J = 6.0 Hz, 1H), 7.87 (s, 1H), 7.56 (t, J = 7.8 Hz, 1H), 6.71 (t, J = 1.2 Hz, 1H), 6.04 (m, 1H), 5.35 (dd, J = 17.2, 1.5 Hz, 1H), 5.24 (dd, J = 10.5, 1.4 Hz, 1H), 4.76 (dt, J = 5.5, 1.5 Hz, 2H), 3.05 (d, J = 5.0 Hz, 3H). ^{13}C NMR (126 MHz, $CDCl_3$) δ 166.1, 160.6, 141.8, 138.3, 133.4, 118.5, 117.6, 114.3, 94.9, 63.7, 34.4. ^{11}B NMR (128 MHz, $CDCl_3$) δ -12.29 (t, J = 98.8 Hz). HRMS m/z (DART+) calcd for $C_{12}H_{16}BN_2O_2$ ([M] + H) 231.12993 found 231.13040.

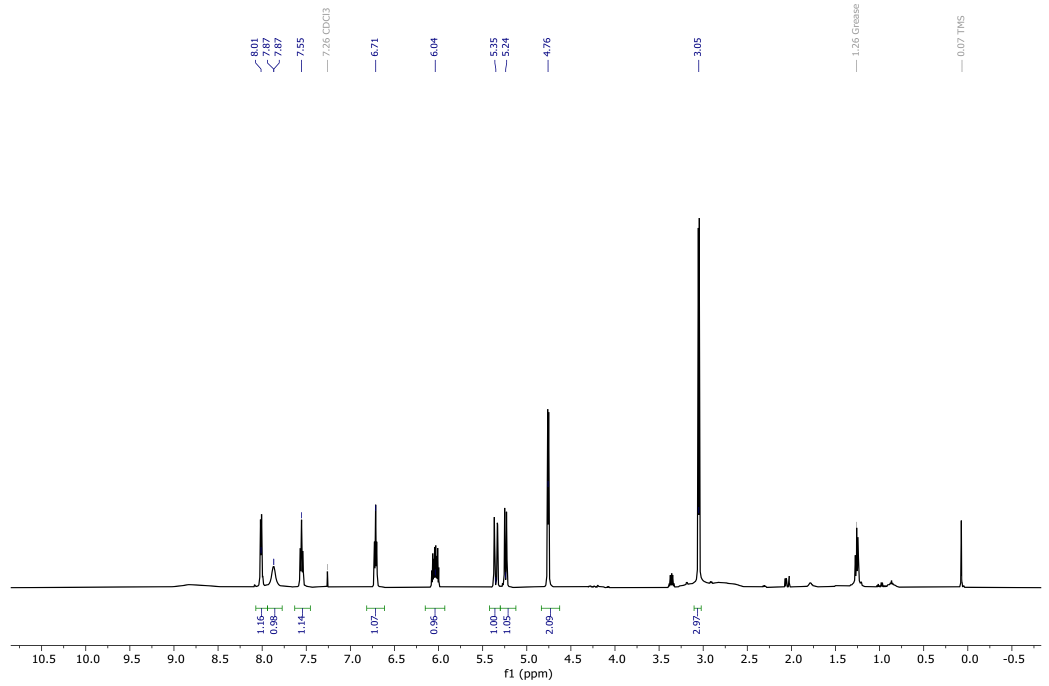

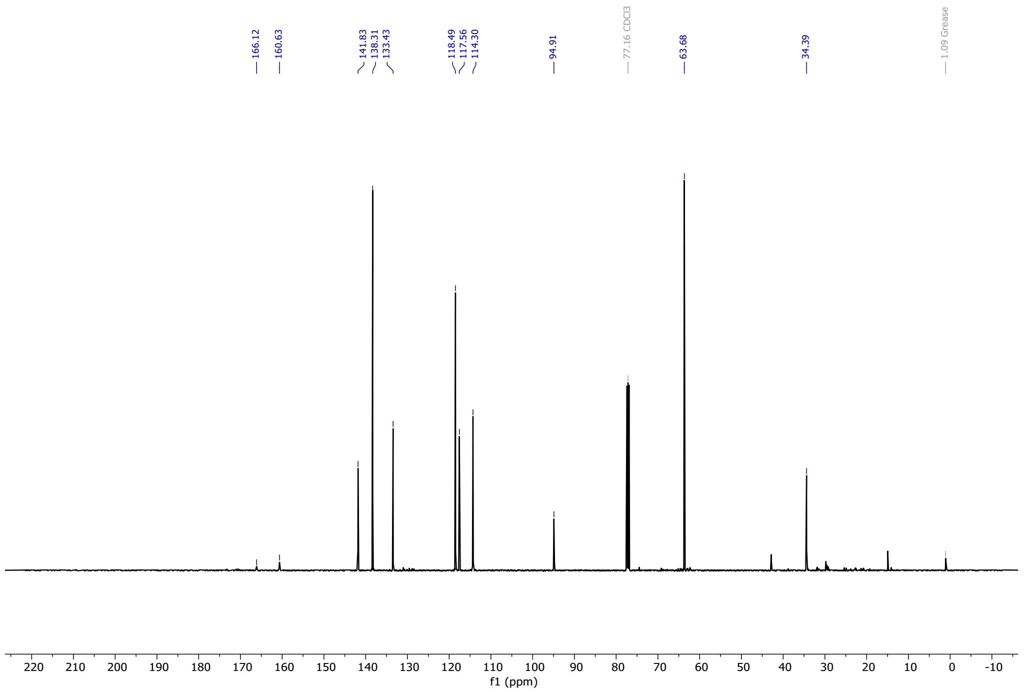

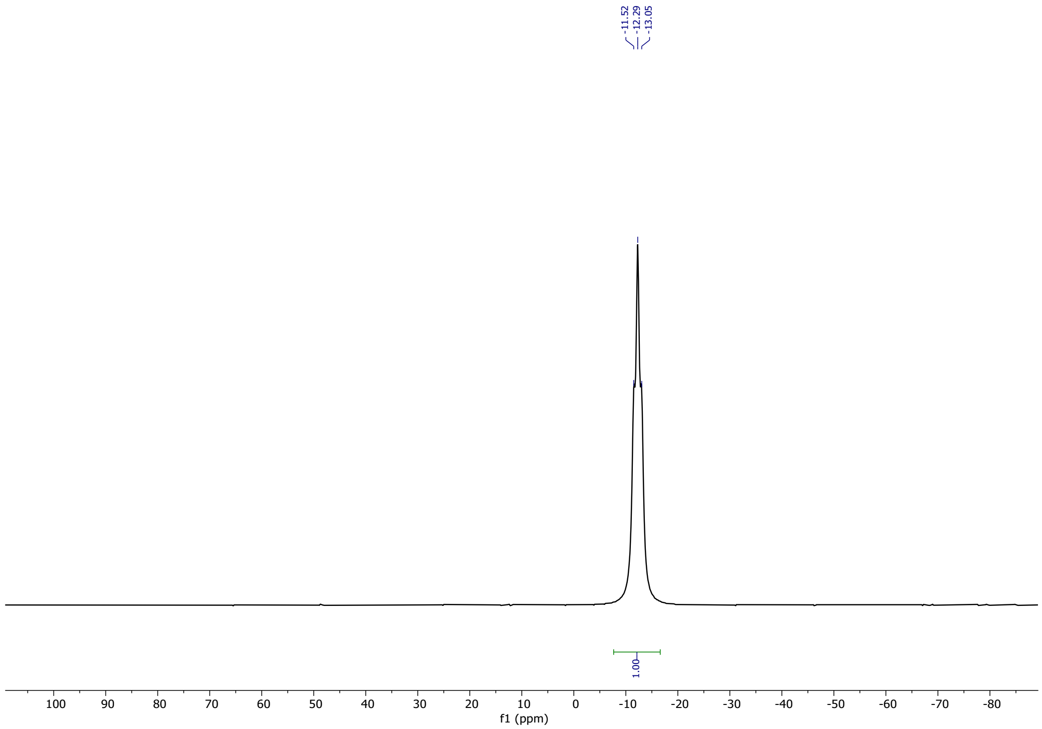

Figure S11: ^{1}H, ^{13}C and ^{11}B NMR of carboxyborindolizine **3b** in $CDCl_3$ (298K)

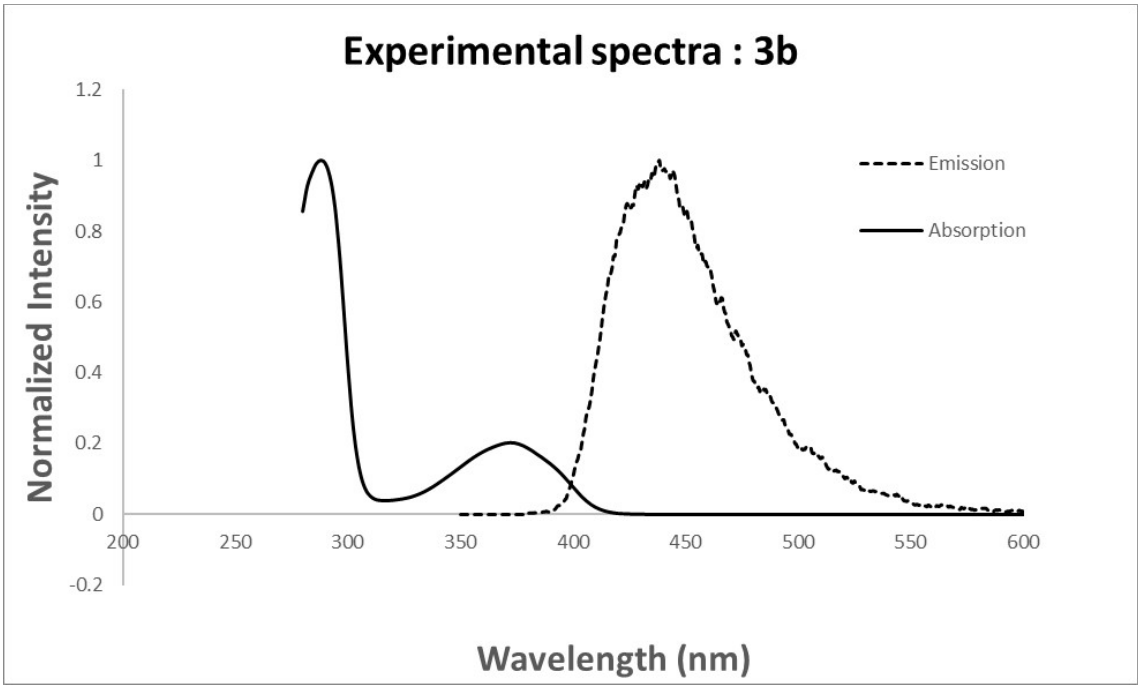

Figure S12: Overlayed absorption and emission spectra of carboxyborindolizine **3b** in dichloromethane.

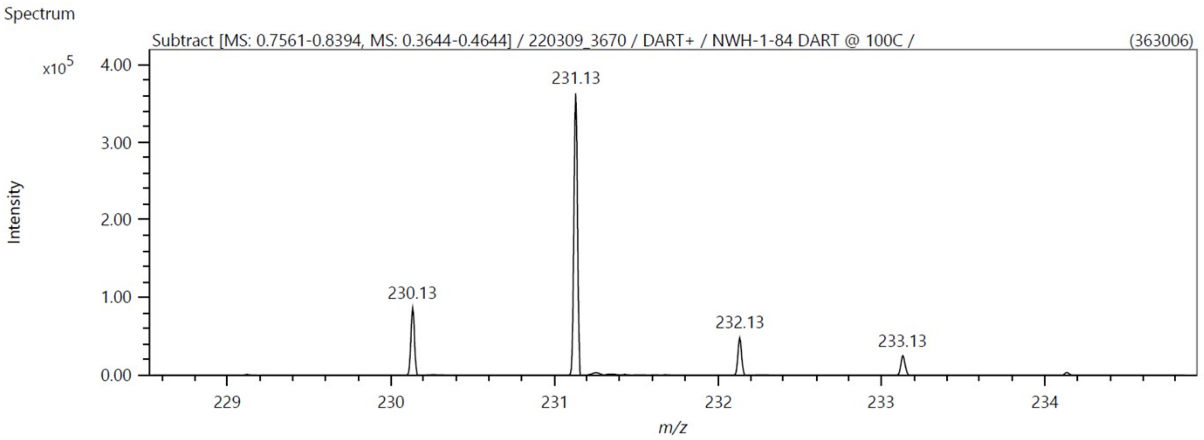

Elemental Composition

Parameters	
Tolerance:	±10.00 mDa
Electron:	Odd/Even
Charge:	+1
DBE:	-1.5 - 100.0

Elements Set 1:

Symbol	C	H	O	N	B	S
Min	0	0	0	0	1	0
Max	100	100	20	10	1	1

Results

Mass	Intensity	Formula	Calculated Mass	Mass Difference [mDa]	Mass Difference [ppm]	DBE
231.13040	363005.86	C5 H16 B N8 S	231.13062	-0.22	-0.97	2.5
		C12 H16 B N2 O2	231.12993	0.46	2.00	6.5
		C7 H18 B N5 O S	231.13196	-1.57	-6.78	2.0
		C10 H14 B N5 O	231.12859	1.80	7.81	7.0
		C H16 B N8 O5	231.13312	-2.73	-11.80	-1.5
		C9 H20 B N2 O2 S	231.13331	-2.91	-12.59	1.5

Figure S13: HRMS of carboxyborindolizine **3b** (DART+)

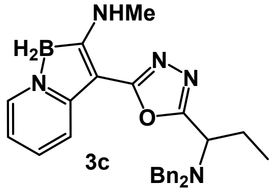

3c

Oxadiazole borindolizine 3c: : Yield: 33%, yellow solid; ^{1}H NMR (400 MHz, $CDCl_3$) δ 8.55 (s, 1H), 8.01 (d, *J* = 5.9 Hz, 1H), 7.58 (m, 2H), 7.34 (m, 4H), 7.24 (t, *J* = 7.4 Hz, 4H), 7.17 (m, 2H), 6.66 (t, *J* = 1.4 Hz, 1H), 3.89 (m, 3H), 3.31 (d, *J* = 13.8 Hz, 2H), 3.08 (d, *J* = 4.9 Hz, 3H), 2.00 (m, 2H), 0.93 (t, *J* = 7.3 Hz, 3H). ^{13}C NMR (101 MHz, $CDCl_3$) δ 163.0, 160.8, 159.1, 142.6, 139.4, 138.7, 129.0, 128.4, 127.2, 115.9, 113.9, 89.5, 55.9, 54.5, 34.7, 23.6, 11.3. ^{11}B NMR (128 MHz, $CDCl_3$) δ -11.98. HRMS m/z (DART+) calcd for $C_{27}H_{31}BN_5O$ ([M] + H) 452.26162 found 452.26183.

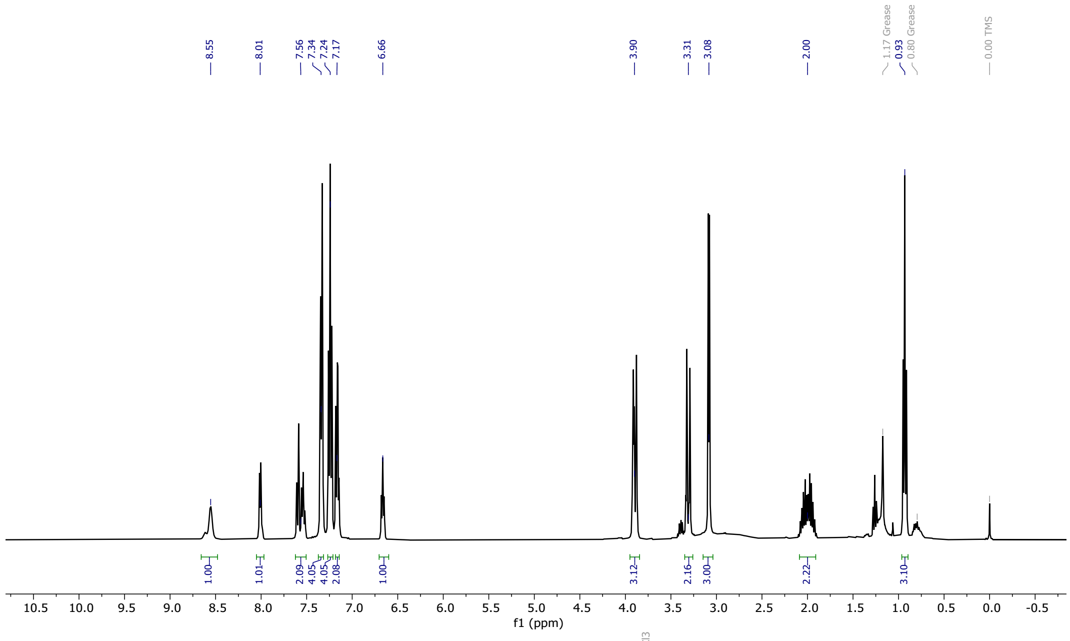
8.55
8.01
7.56
7.34
7.24
7.17
6.66
3.90
3.31
3.08
2.00
1.17 Grease
0.93
0.80 Grease
0.00 TMS
1.00
1.01
2.09
4.05
4.05
2.08
1.00
3.12
2.16
3.00
2.22
3.10
10.5 10.0 9.5 9.0 8.5 8.0 7.5 7.0 6.5 6.0 5.5 5.0 4.5 4.0 3.5 3.0 2.5 2.0 1.5 1.0 0.5 0.0 -0.5
f1 (ppm)

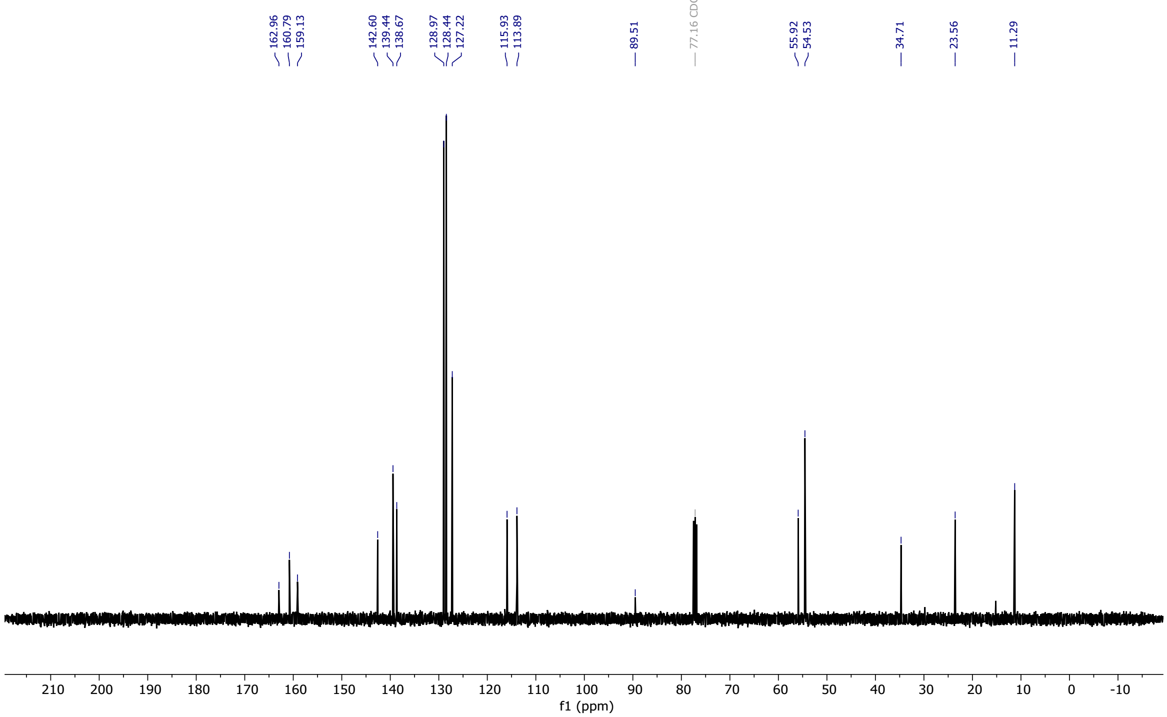
162.96
160.79
159.13
142.60
139.44
138.67
128.97
128.44
127.22
115.93
113.89
89.51
77.16 CDCl3
55.92
54.53
34.71
23.56
11.29
210 200 190 180 170 160 150 140 130 120 110 100 90 80 70 60 50 40 30 20 10 0 -10
f1 (ppm)

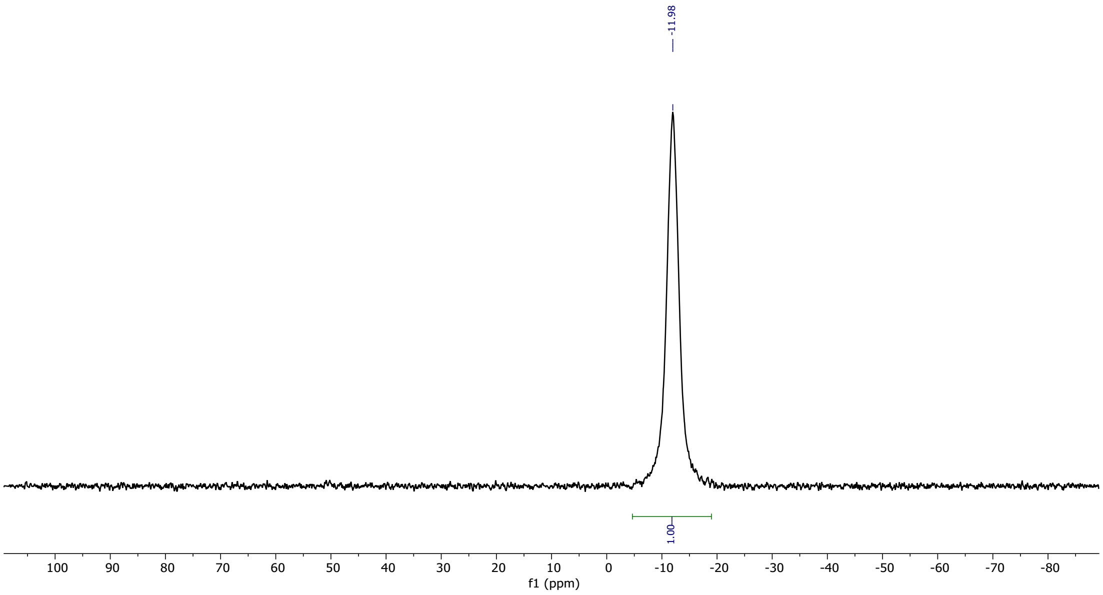

Figure S14: ^{1}H, ^{13}C and ^{11}B NMR of carboxyborindolizine **3c** in $CDCl_3$ (298K)

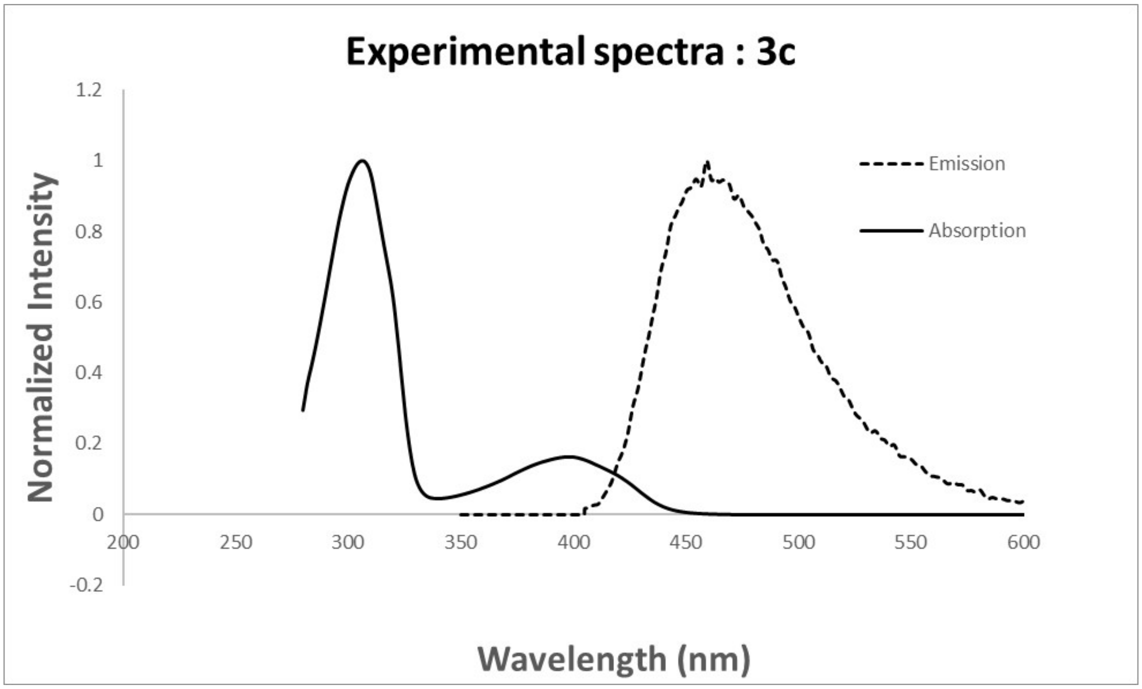

Figure S15: Overlayed absorption and emission spectra of oxadiazole borindolizine **3c** in dichloromethane.

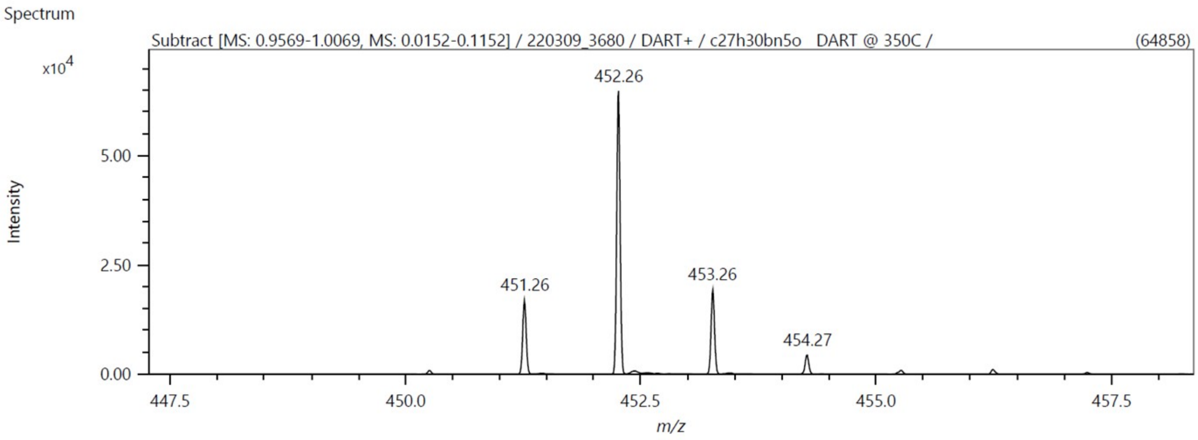

Elemental Composition

Parameters		Elements Set 1:					
Tolerance:	±10.00 mDa	Symbol	C	H	O	N	B
Electron:	Odd/Even	Min	0	0	0	0	1
Charge:	+1	Max	100	100	20	10	1
DBE:	-1.5 - 100.0						

Results

Mass	Intensity	Formula	Calculated Mass	Mass Difference [mDa]	Mass Difference [ppm]	DBE
452.26183	64858.28	C27 H31 B N5 O	452.26162	0.21	0.47	15.5
		C13 H33 B N10 O7	452.26213	-0.30	-0.66	3.0
		C29 H33 B N2 O2	452.26296	-1.13	-2.50	15.0
		C26 H35 B N O5	452.26028	1.55	3.42	10.5
		C25 H29 B N8	452.26027	1.55	3.44	16.0
		C15 H35 B N7 O8	452.26347	-1.64	-3.62	2.5

Figure S16: HRMS of carboxyborindolizine **3c** (DART+)

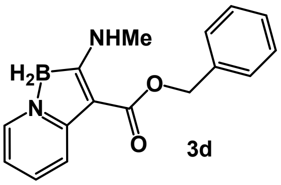

Carboxyborindolizine 3d: Yield: 68%, yellow liquid; ^{1}H NMR (500 MHz, $CDCl_3$) δ 8.03 (d, J = 0.9 Hz, 1H), 7.88 (s, 1H), 7.53 (t, J = 7.8 Hz, 1H), 7.43 (m, 2H), 7.38 (m, 2H), 7.33 (m, 1H), 6.72 (t, J = 1.2 Hz, 1H), 5.32 (s, 2H), 3.06 (d, J = 5.0 Hz, 3H). ^{13}C NMR (126 MHz, $CDCl_3$) δ 160.7, 141.9, 138.4, 137.3, 128.7, 128.0, 128.0, 118.5, 114.4, 94.9, 64.8, 34.5. ^{11}B NMR (128 MHz, $CDCl_3$) δ -12.25 (t, J = 102.5 Hz). HRMS m/z (DART+) calcd for $C_{16}H_{18}BN_2O_2$ ([M] + H) 281.14558 found 281.14687.

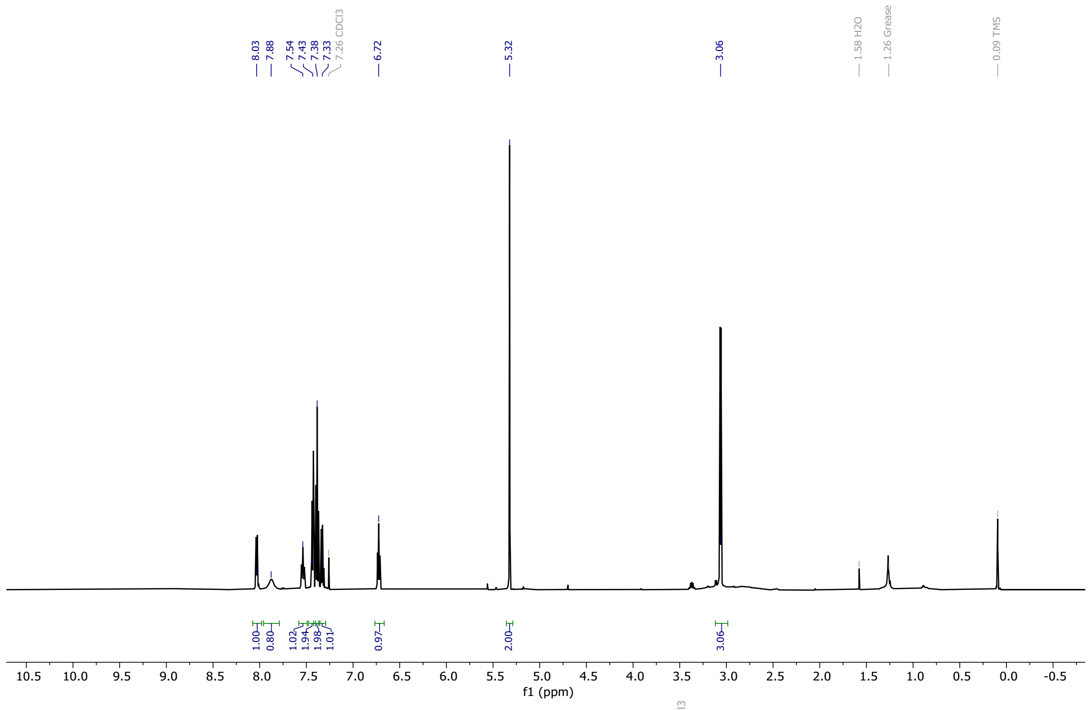
8.03
7.88
7.54
7.43
7.38
7.33
7.26 CDCl3
6.72
5.32
3.06
1.58 H2O
1.26 Grease
0.09 TMS
1.00
0.80
1.02
1.94
1.98
1.01
0.97
2.00
3.06
10.5 10.0 9.5 9.0 8.5 8.0 7.5 7.0 6.5 6.0 5.5 5.0 4.5 4.0 3.5 3.0 2.5 2.0 1.5 1.0 0.5 0.0 -0.5
f1 (ppm)

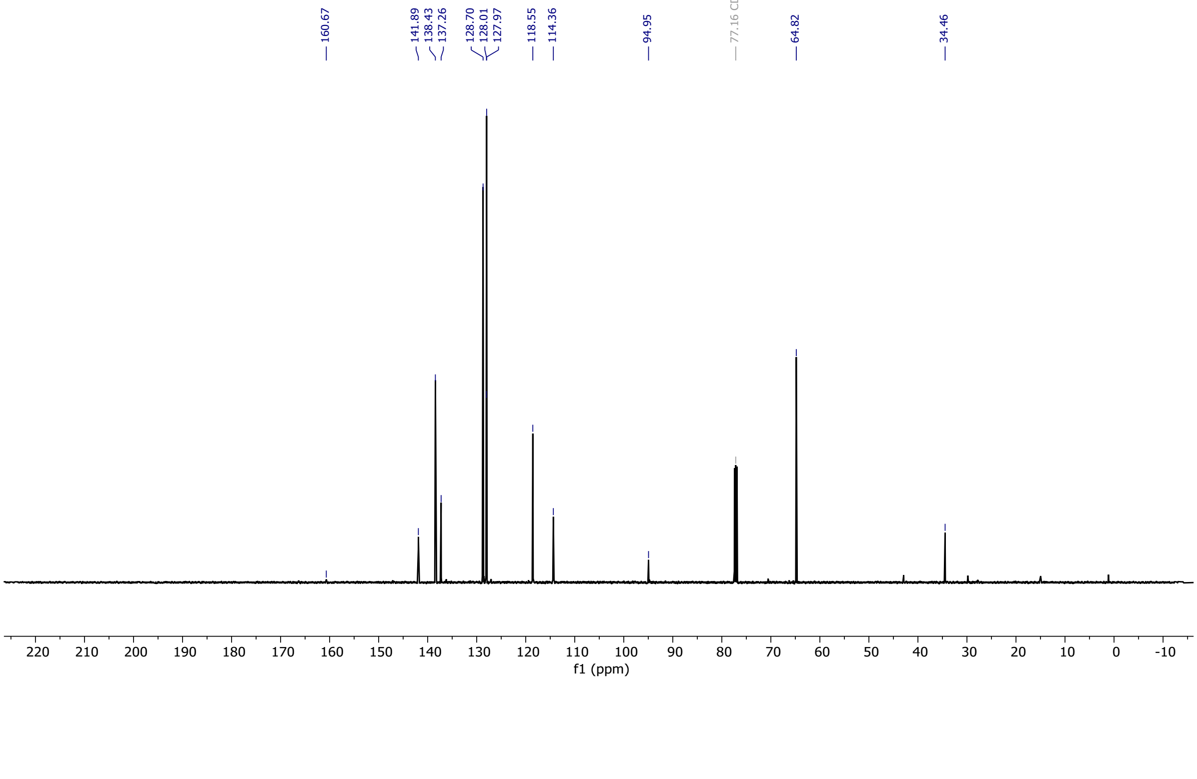
160.67
141.89
138.43
137.26
128.70
128.01
127.97
118.55
114.36
94.95
77.16 CDCl3
64.82
34.46
220 210 200 190 180 170 160 150 140 130 120 110 100 90 80 70 60 50 40 30 20 10 0 -10
f1 (ppm)

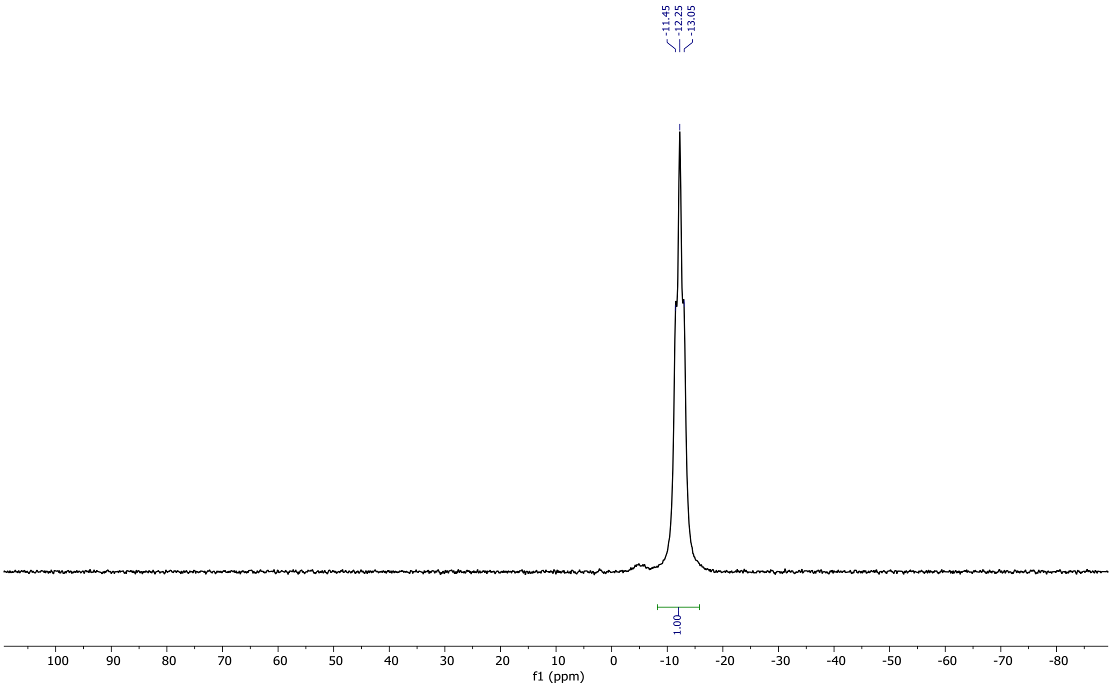

Figure S17: ^{1}H, ^{13}C and ^{11}B NMR of carboxyborindolizine **3d** in $CDCl_3$ (298K)

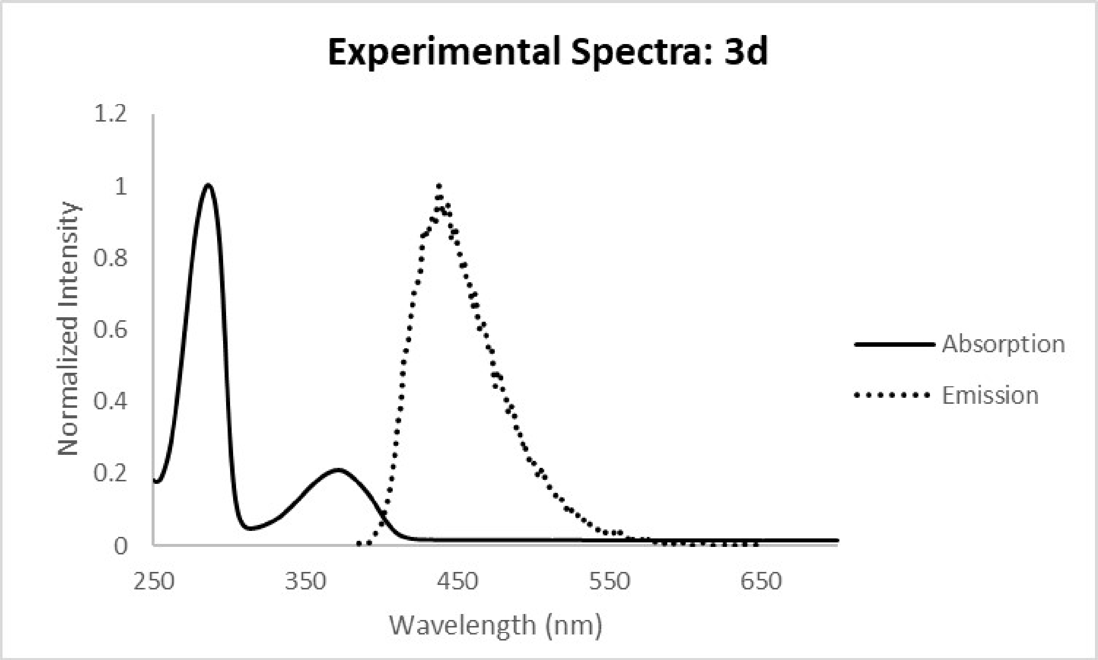

Figure S18: Overlayed absorption and emission spectra of borindolizine **3d** in dichloromethane.

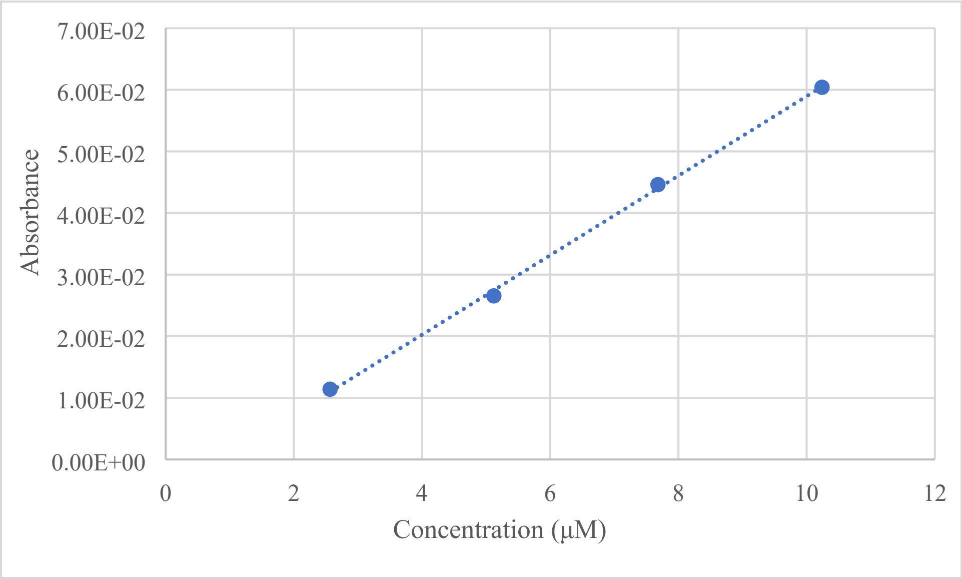

Figure 19: Molar absorptivity of carboxyborindolizine **3d** in dichloromethane (λ= 371 nm, $\varepsilon = 6.5 \times 10^3\ M^{-1}cm^{-1}$)

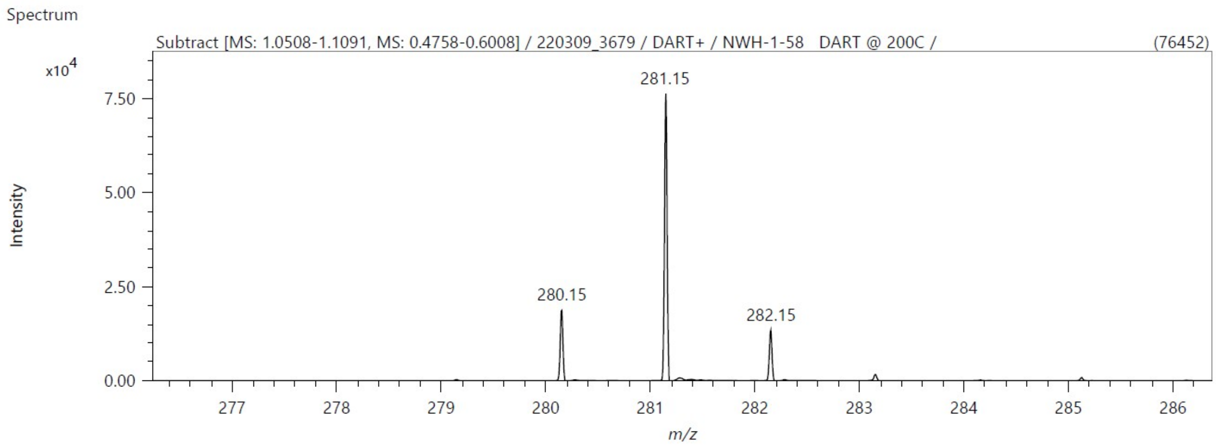

Elemental Composition

Parameters

Tolerance:	±10.00 mDa
Electron:	Odd/Even
Charge:	+1
DBE:	-1.5 - 100.0

Elements Set 1:

Symbol	C	H	O	N	B
Min	0	0	0	0	1
Max	100	100	20	10	1

Results

Mass	Intensity	Formula	Calculated Mass	Mass Difference [mDa]	Mass Difference [ppm]	DBE
281.14687	76452.25	C16 H18 B N2 O2	281.14558	1.29	4.57	9.5
		C5 H18 B N8 O5	281.14877	-1.90	-6.76	1.5
		C14 H16 B N5 O	281.14424	2.63	9.35	10.0
		C21 H18 B	281.14961	-2.74	-9.73	13.5
		C7 H20 B N5 O6	281.15012	-3.24	-11.54	1.0
		C13 H20 B N O5	281.14290	3.97	14.11	5.0

Figure S20: HRMS of carboxyborindolizine **3d** (DART+)

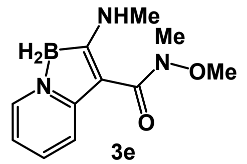

3e

Carboxyborindolizine 3e: Yield: 14%, yellow liquid; ^{1}H NMR (400 MHz, $CDCl_3$) δ 8.15 (s, 1H), 8.00 (d, J = 6.0 Hz, 1H), 7.52 (t, J = 7.8 Hz 1H), 7.33 (d, J = 8.8z Hz, 1H), 6.67 (t, J = 1.2 Hz, 1H), 3.57 (s, 3H), 3.17 (s, 3H), 3.03 (d, J = 5.0 Hz, 3H). ^{13}C NMR (126 MHz, $CDCl_3$) δ 170.1, 160.4, 141.7, 137.6, 118.5, 113.5, 97.4, 60.3, 36.4, 34.4. ^{11}B NMR (128 MHz, $CDCl_3$) δ -12.44 (t, J = 101.0 Hz). HRMS m/z (DART+) calcd for $C_{11}H_{17}BN_3O_2$ ([M] + H) 234.14083 found 234.14138.

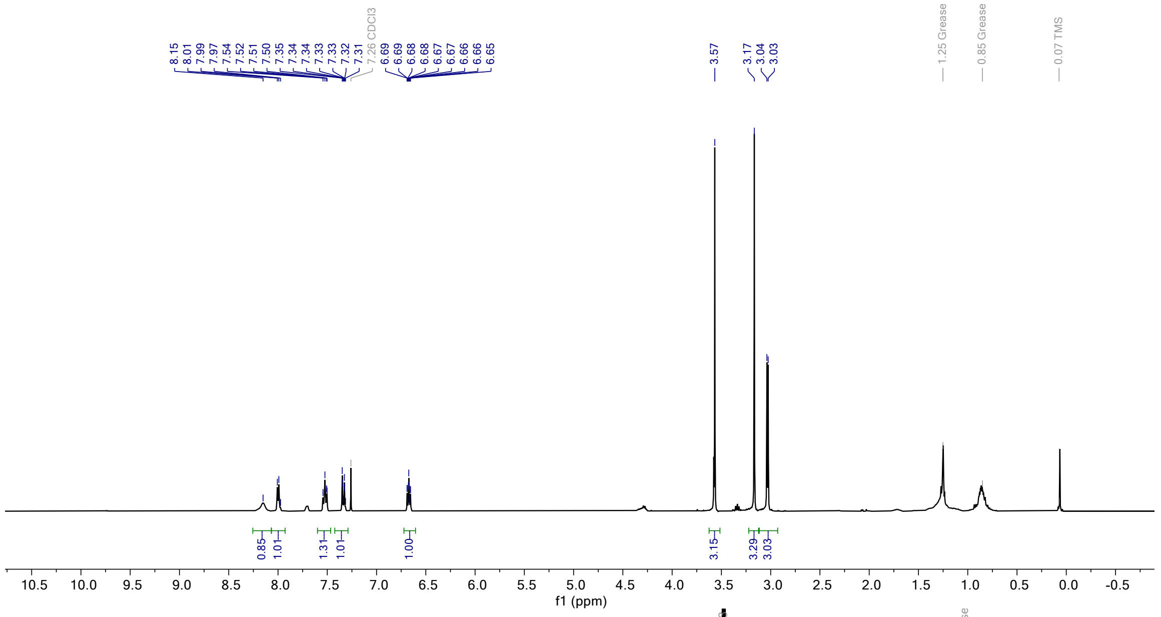

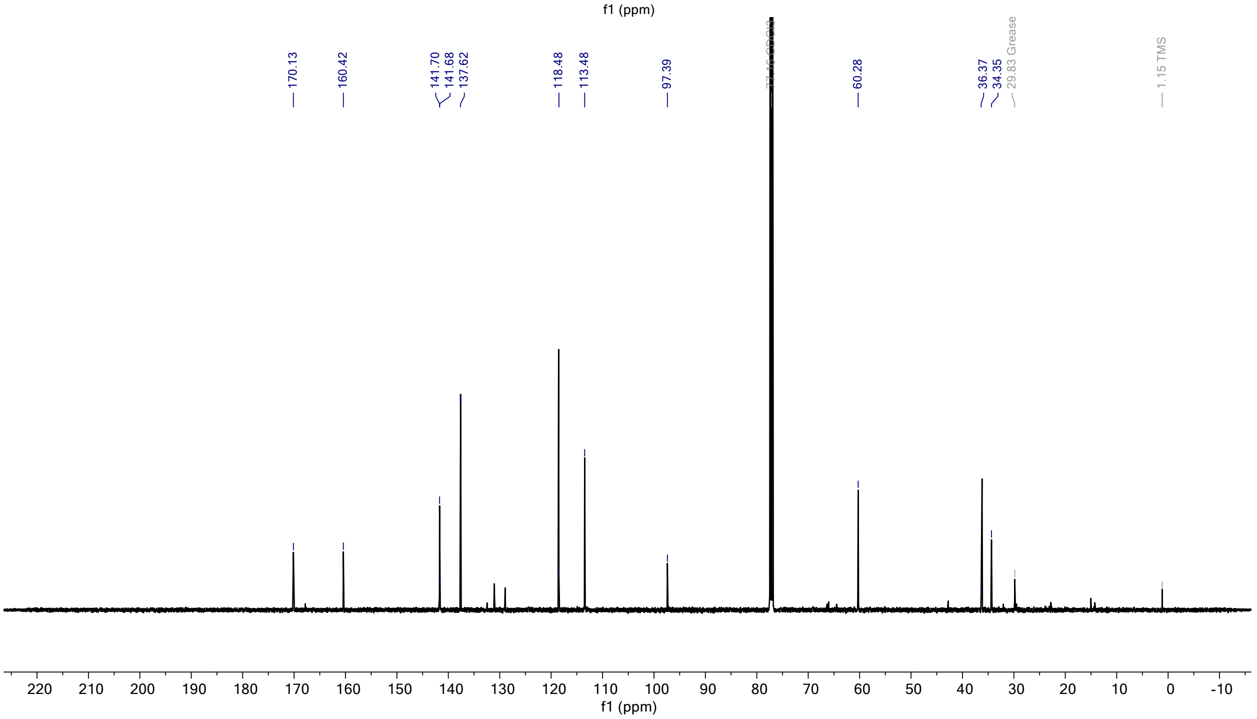

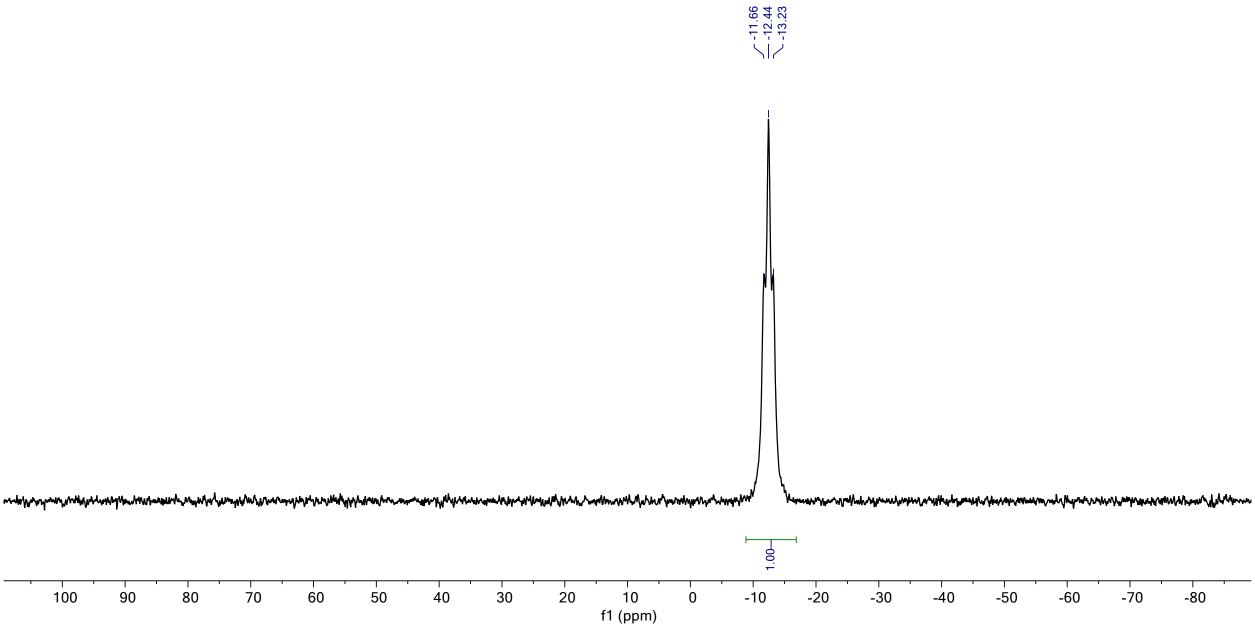

Figure S21: ^{1}H, ^{13}C and ^{11}B NMR of carboxyborindolizine **3e** in $CDCl_3$ (298K)

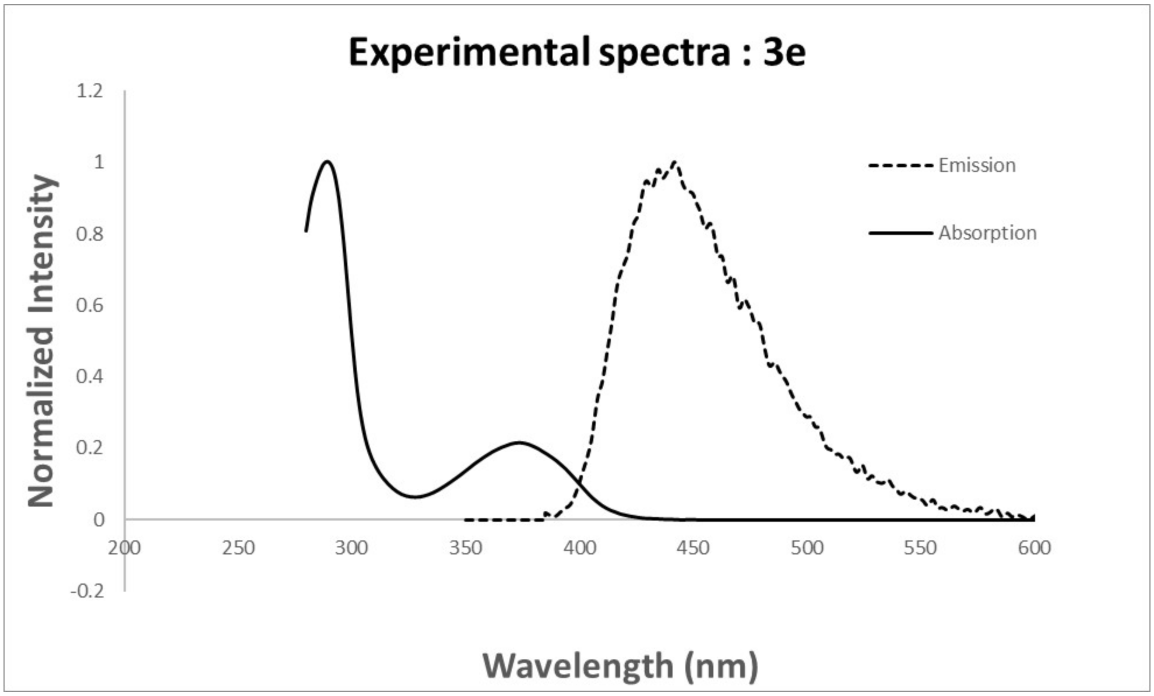

Figure S22: Overlayed absorption and emission spectra of borindolizine **3e** in dichloromethane.

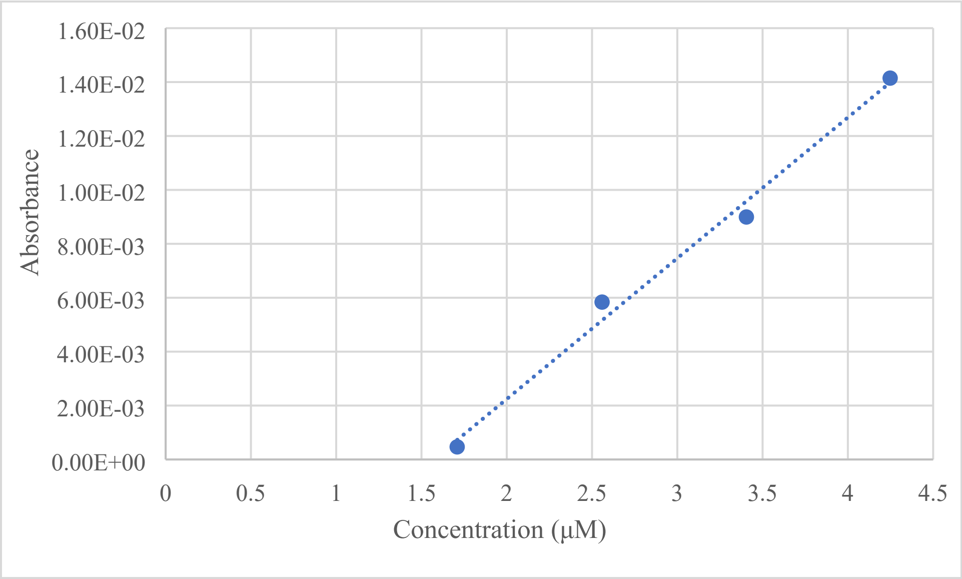

Figure 23: Molar absorptivity of carboxyborindolizine **3e** in dichloromethane (λ= 374 nm, $\varepsilon = 5.2 \times 10^3$ $M^{-1}cm^{-1}$)

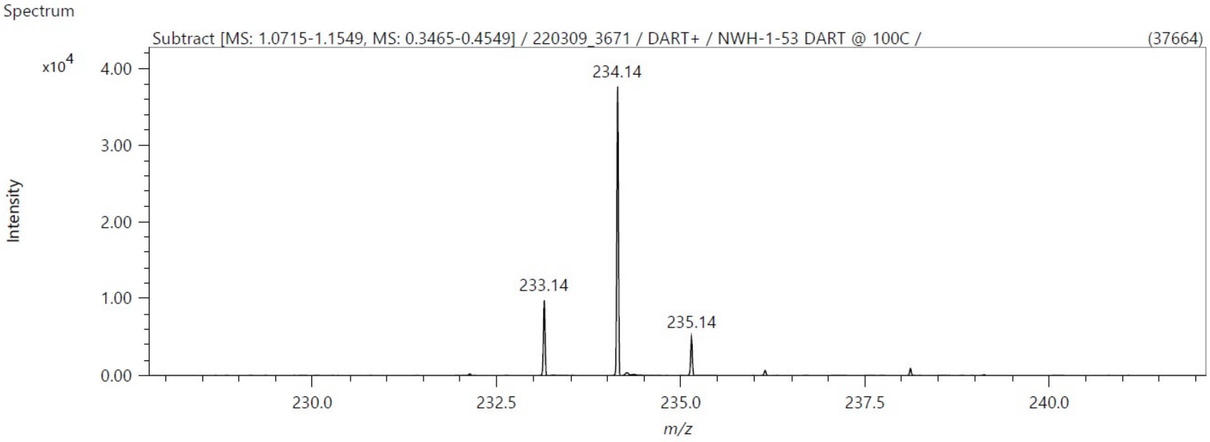

Elemental Composition

Parameters	
Tolerance:	±10.00 mDa
Electron:	Odd/Even
Charge:	+1
DBE:	-1.5 - 100.0

Elements Set 1:

Symbol	C	H	O	N	B	S
Min	0	0	0	0	1	0
Max	100	100	20	10	1	1

Results

Mass	Intensity	Formula	Calculated Mass	Mass Difference [mDa]	Mass Difference [ppm]	DBE
234.14138	37663.59	C4 H17 B N9 S	234.14152	-0.14	-0.60	1.5
		C11 H17 B N3 O2	234.14083	0.54	2.32	5.5
		C13 H19 B O3	234.14218	-0.80	-3.41	5.0
		C6 H19 B N6 O S	234.14286	-1.48	-6.34	1.0
		C9 H15 B N6 O	234.13949	1.89	8.06	6.0
		C8 H21 B N3 O2 S	234.14420	-2.83	-12.07	0.5

Figure S24: HRMS of carboxyborindolizine **3e** (DART+)

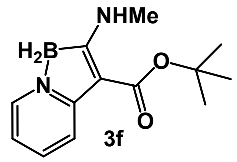

3f

Carboxyborindolizine 3f: Yield: 11%, yellow liquid; ^{1}H NMR (500 MHz, $CDCl_3$) δ 8.01 (d, *J* = 5.9 Hz, 1H), 7.84 (s, 1H), 7.55 (t, *J* = 7.9 Hz, 1H), 6.70 (t, *J* = 1.2 Hz, 1H), 3.04 (d, *J* = 5.0 Hz, 3H), 1.59 (s, 9H). ^{13}C NMR (126 MHz, $CDCl_3$) δ 161.0, 141.9, 141.9, 138.2, 118.4, 114.0, 96.3, 79.7, 34.3, 28.9. ^{11}B NMR (128 MHz, $CDCl_3$) δ -12.45 (t, *J* = 100.0 Hz). HRMS m/z (DART+) calcd for $C_{13}H_{20}BN_2O_2$ ([M] + H) 247.16123 found 247.16175.

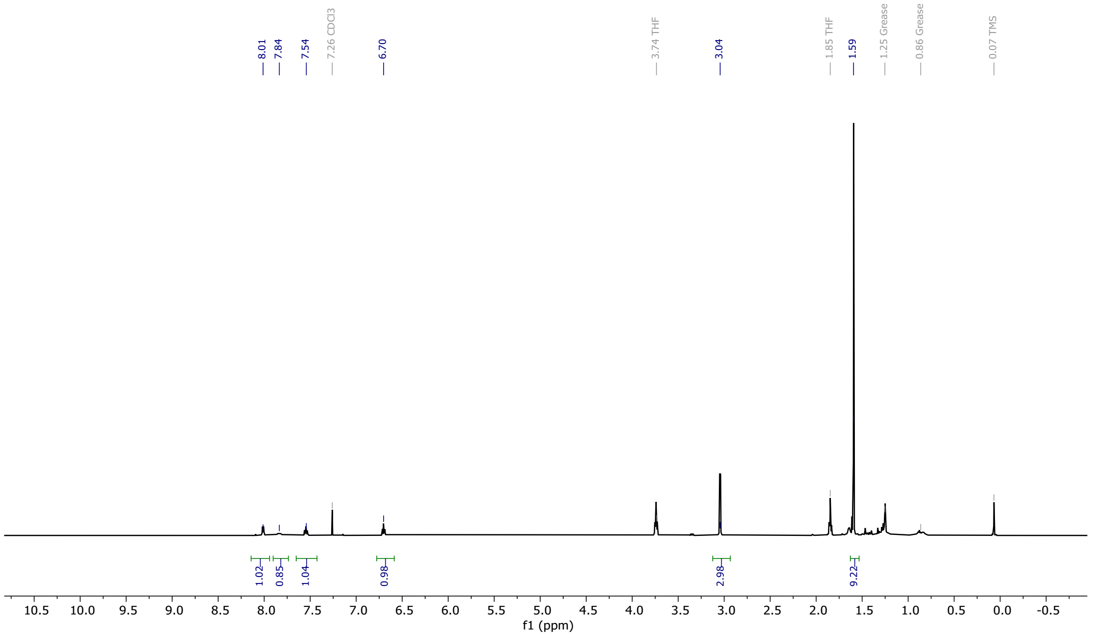

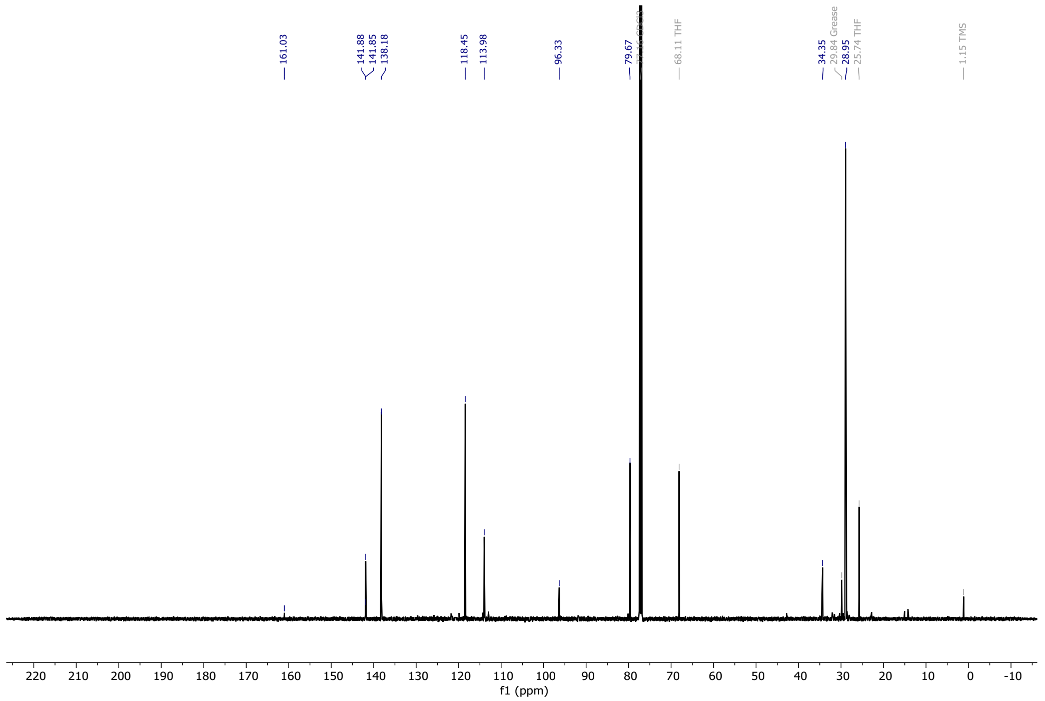

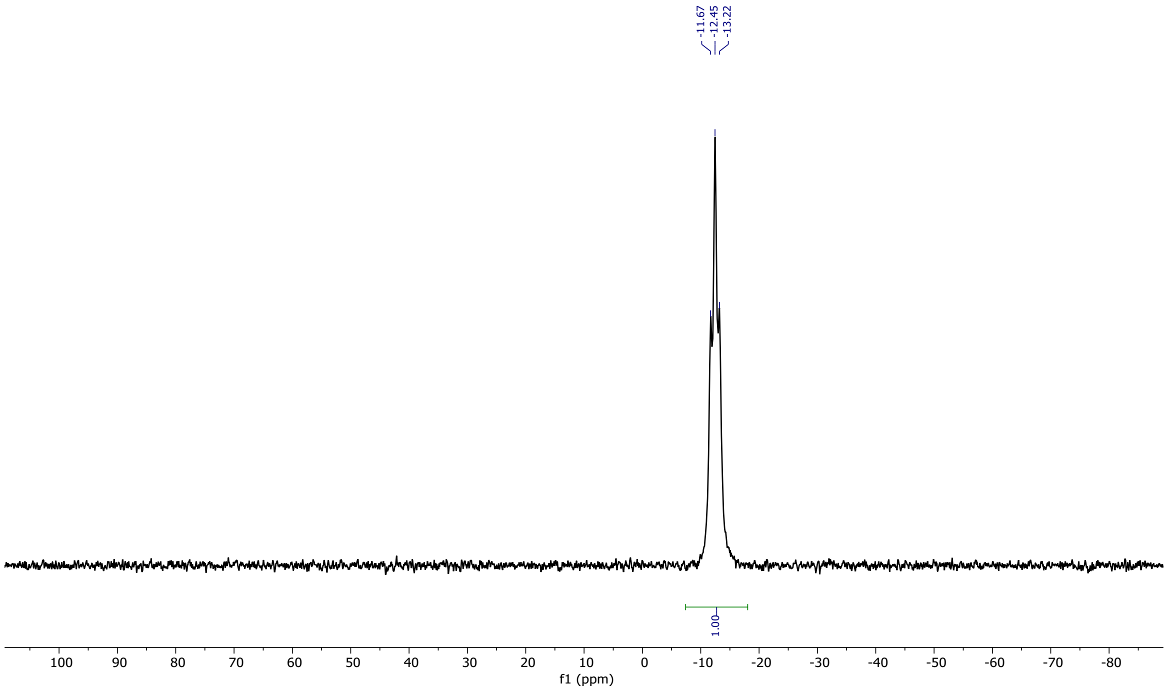

Figure S25: ^{1}H, ^{13}C and ^{11}B NMR of carboxyborindolizine **3f** in $CDCl_3$ (298K)

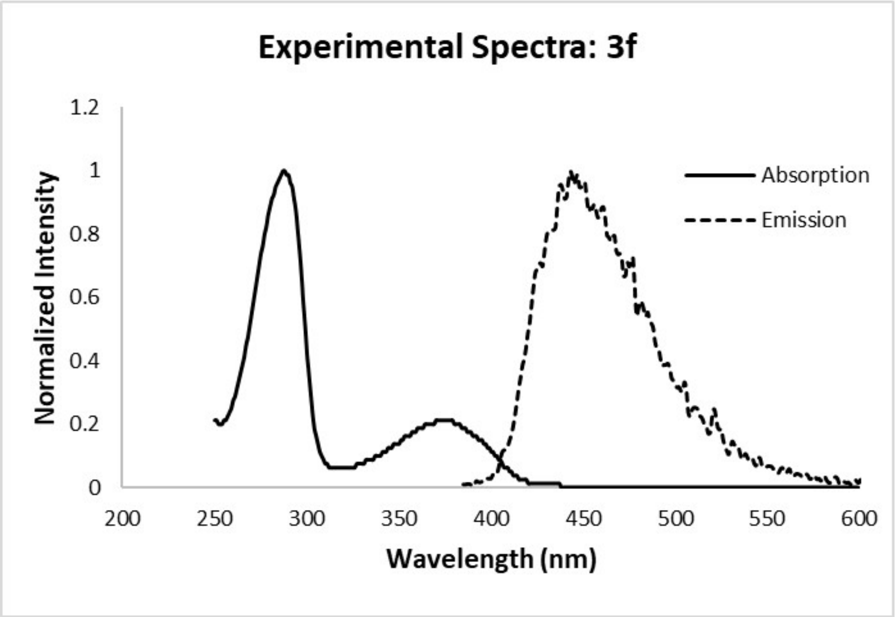

Figure S26: Overlayed absorption and emission spectra of borindolizine **3f** in dichloromethane.

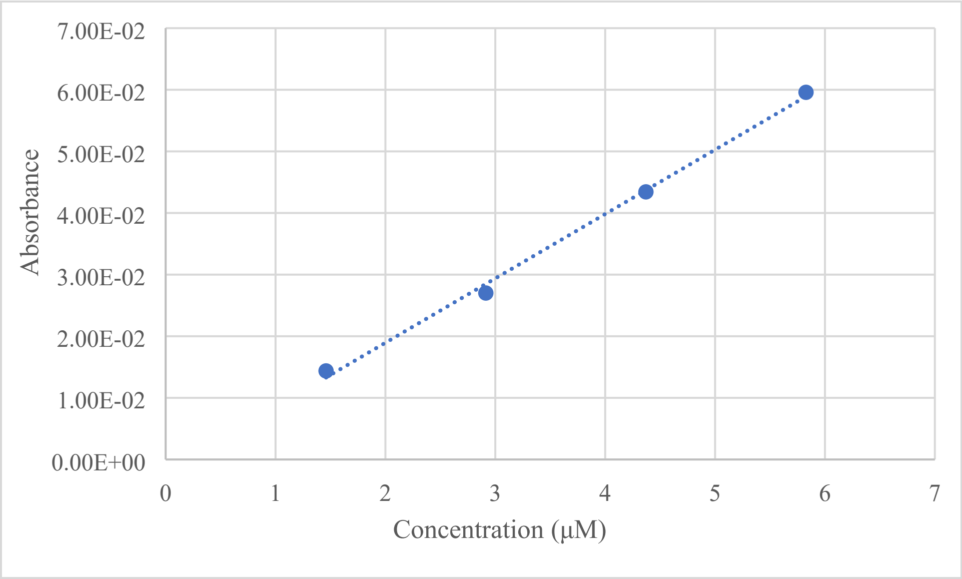

Figure 27: Molar absorptivity of carboxyborindolizine **3f** in dichloromethane (λ= 375 nm, ε = 10.4 $\times$ 10^3 $M^{-1}cm^{-1}$)

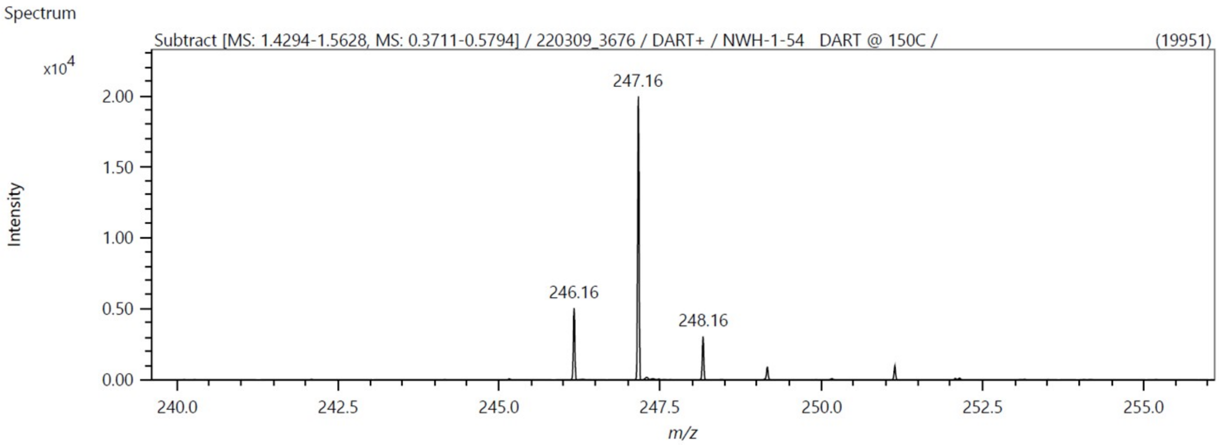

Elemental Composition

Parameters

Tolerance:	±10.00 mDa
Electron:	Odd/Even
Charge:	+1
DBE:	-1.5 - 100.0

Elements Set 1:

Symbol	C	H	O	N	B	F
Min	0	0	0	0	1	0
Max	100	100	20	10	1	1

Results

Mass	Intensity	Formula	Calculated Mass	Mass Difference [mDa]	Mass Difference [ppm]	DBE
247.16175	19950.55	C13 H20 B N2 O2	247.16123	0.51	2.07	5.5
		C10 H21 B N2 O3 F	247.16238	-0.63	-2.56	1.5
		C8 H19 B N5 O2 F	247.16104	0.71	2.88	2.0
		C11 H18 B N5 O	247.15989	1.85	7.50	6.0
		C6 H17 B N8 O F	247.15969	2.05	8.31	2.5
		C10 H22 B N O5	247.15855	3.19	12.91	1.0

Figure S28: HRMS of carboxyborindolizine **3f** (DART+)

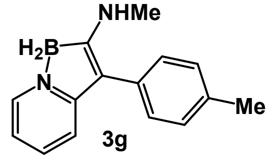

3g

Aryl borindolizine 3g: yield = 68%, red oil. ^{1}H NMR (600 MHz, $CDCl_3$) δ 8.02 (d, J = 5.9 Hz, 1H), 7.34 (t, J = 1.5 Hz, 1H), 7.27 (m, 4H), 6.97 (d, J = 8.5 Hz, 1H), 6.51 (t, J = 1.2 Hz, 1H), 5.37 (s, 1H), 2.99 (d, J = 4.9 Hz, 3H), 2.42 (s, 3H). ^{13}C NMR (151 MHz, $CDCl_3$) δ 162.9, 142.3, 137.5, 135.7, 130.1, 129.1, 129.1, 114.0, 111.4, 105.9, 34.1, 21.3. ^{11}B NMR (192 MHz, $cdcl_3$) δ -12.60. HRMS m/z (DART+) calcd for $C_{15}H_{18}BN_2$ ([M] + H) 237.15576 found 237.15653.

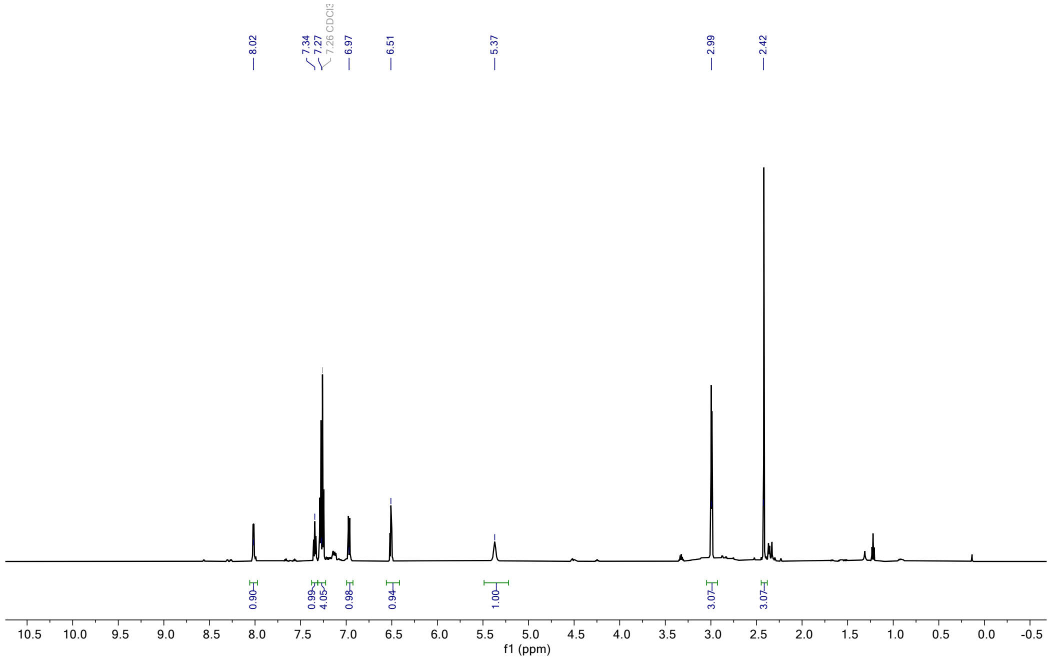

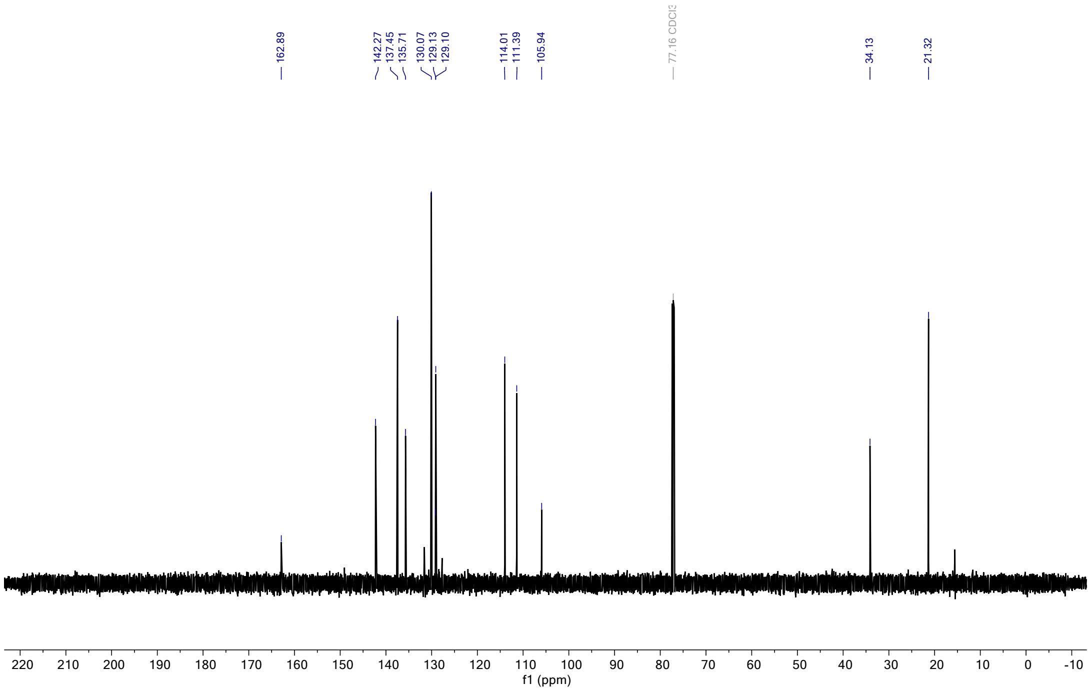

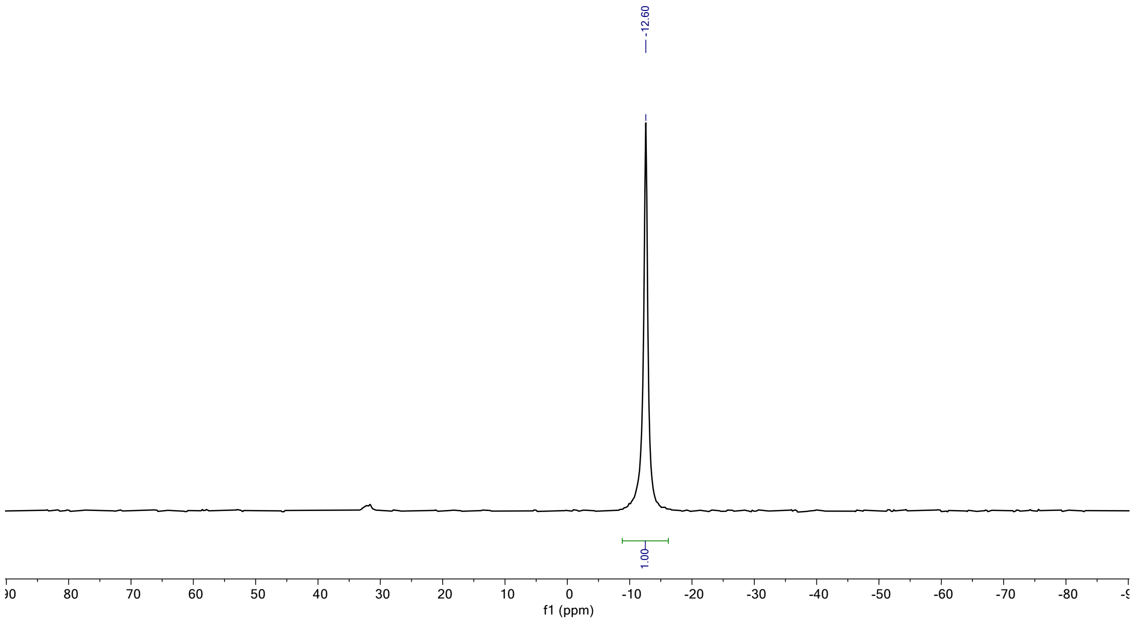

Figure S29: ^{1}H, ^{13}C and ^{11}B NMR of arylborindolizine **3g** in $CDCl_3$ (298K)

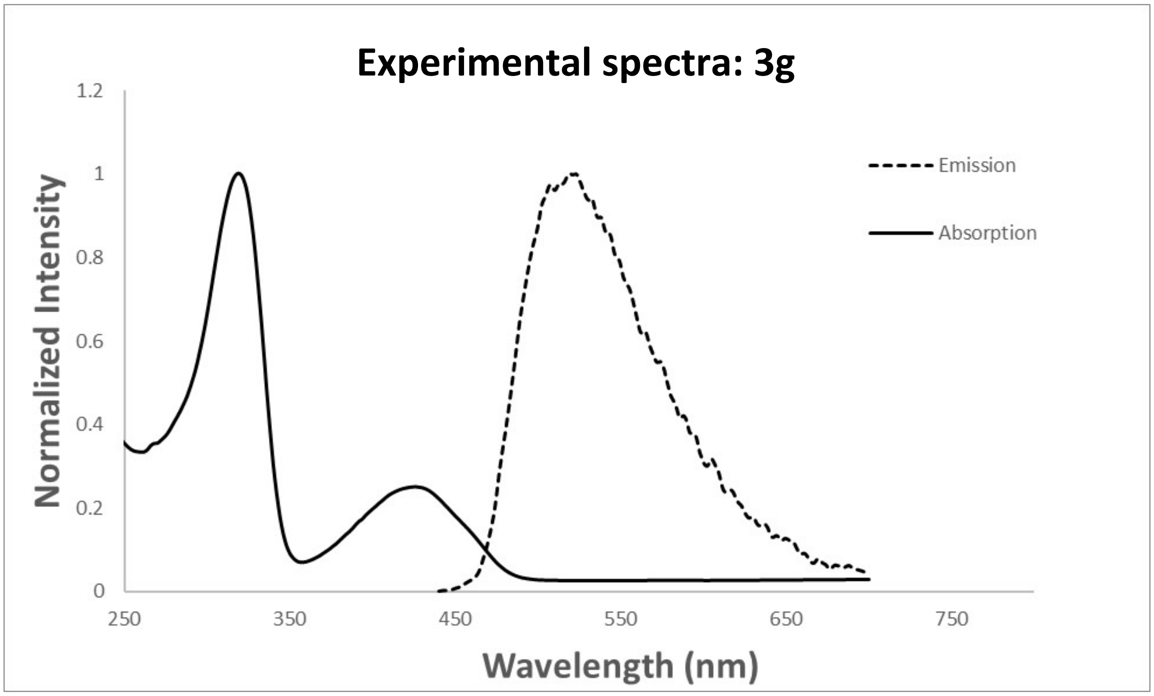

Figure S30: Overlayed absorption and emission spectra of borindolizine **3g** in dichloromethane.

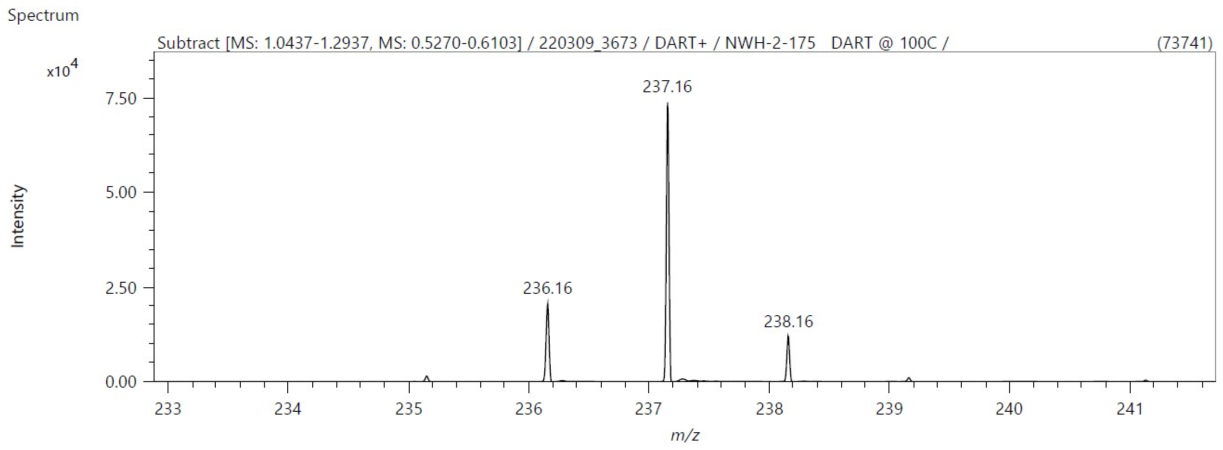

Elemental Composition

Parameters		Elements Set 1:						
Tolerance:	±10.00 mDa	Symbol	C	H	O	N	B	S
Electron:	Odd/Even	Min	0	0	0	0	1	0
Charge:	+1	Max	100	100	20	10	1	1
DBE:	-1.5 - 100.0							

Results

Mass	Intensity	Formula	Calculated Mass	Mass Difference [mDa]	Mass Difference [ppm]	DBE
237.15653	73741.09	C9 H24 B N O3 S	237.15645	0.08	0.36	-1.0
		C15 H18 B N2	237.15576	0.78	3.27	8.5
		C7 H22 B N4 O2 S	237.15510	1.43	6.02	-0.5
		C4 H18 B N8 O3	237.15894	-2.41	-10.17	0.5
		C12 H22 B N2 S	237.15913	-2.60	-10.94	3.5
		C5 H20 B N7 O S	237.15376	2.77	11.68	0.0

Figure S31: HRMS of carboxyborindolizine **3g** (DART+)

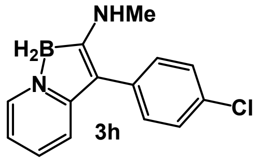

Aryl borindolizine 3h, yield = 44%; ^{1}H NMR (600 MHz, $CDCl_3$) δ 8.00 (d, *J* = 5.9 Hz, 1H), 7.39 (m, 2H), 7.35 (t, *J* = 7.8 Hz, 1H), 7.25 (m, 2H), 6.91 (d, *J* = 8.5 Hz, 1H), 6.52 (t, *J* = 1.2 Hz, 1H), 5.30 (s, 1H), 2.96 (d, *J* = 4.9 Hz, 3H). ^{13}C NMR (126 MHz, $CDCl_3$) δ 162.5, 142.4, 137.8, 133.3, 131.6, 130.6, 129.6, 113.8, 111.8, 104.8, 34.2.^{11}B NMR (192 MHz, $CDCl_3$) δ -12.47. HRMS m/z (DART+) calcd for $C_{14}H_{15}BN_2Cl$ ([M] + H) 257.10113 found 257.10183.

CRAPT ^{13}C NMR was used to characterize 3h to determine which carbon atoms were missing from each ^{13}C spectra

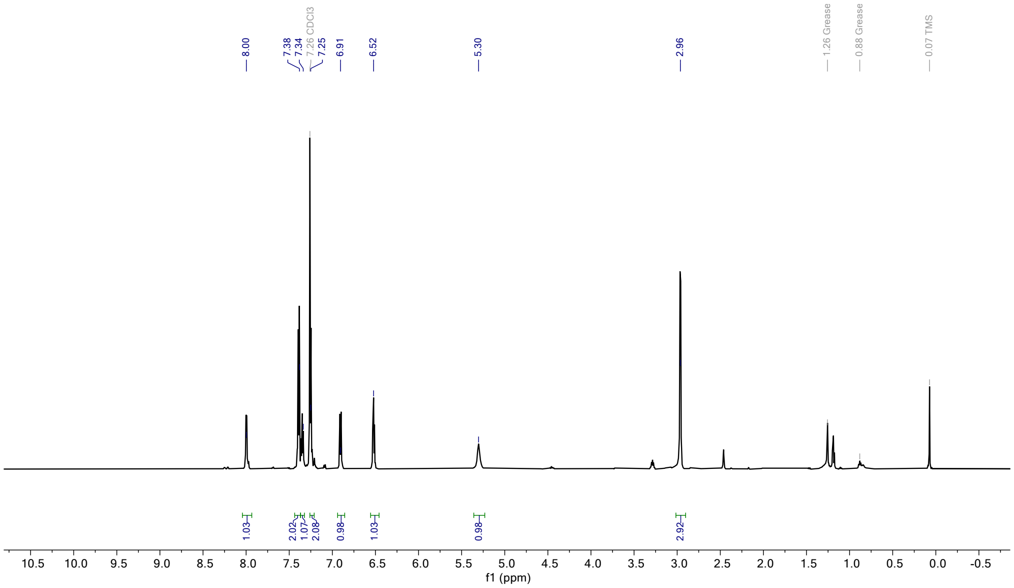
8.00
7.38
7.34
7.26 CDCl3
7.25
6.91
6.52
5.30
2.96
1.26 Grease
0.88 Grease
0.07 TMS
1.03
2.02
1.07
2.08
0.98
1.03
0.98
2.92
f1 (ppm)

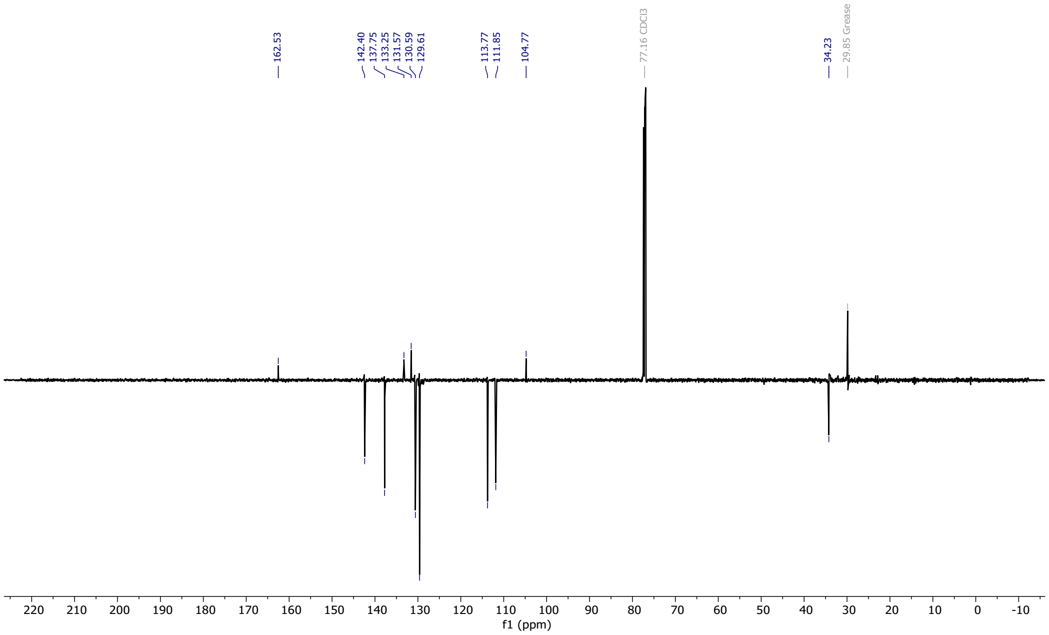
162.53
142.40
137.75
133.25
131.57
130.59
129.61
113.77
111.85
104.77
77.16 CDCl3
34.23
29.85 Grease
f1 (ppm)

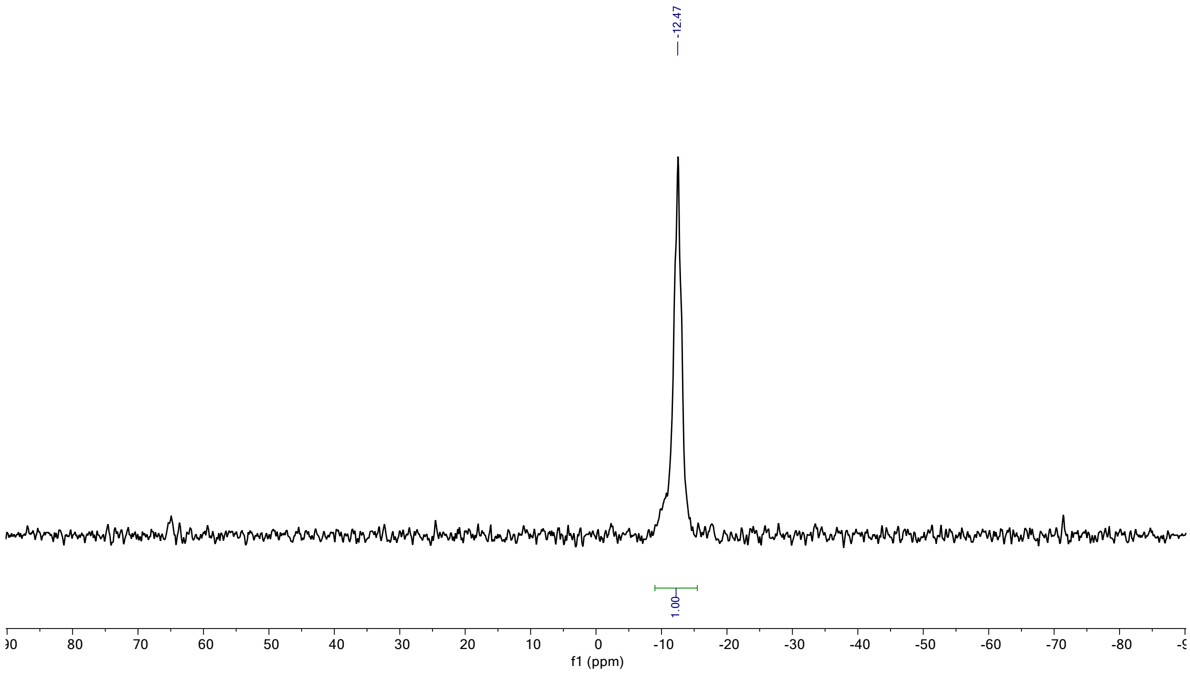

Figure S32: ^{1}H, ^{13}C and ^{11}B NMR of arylborindolizine **3h** in $CDCl_3$ (298K)

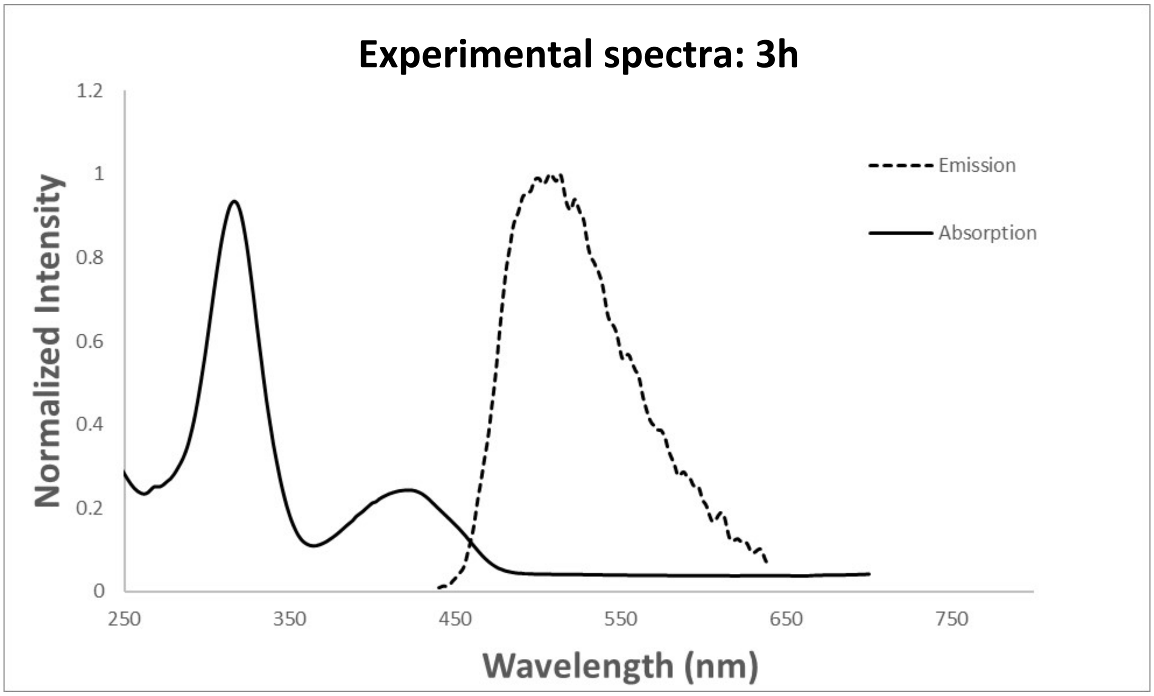

Figure S33: Overlayed absorption and emission spectra of borindolizine **3h** in dichloromethane.

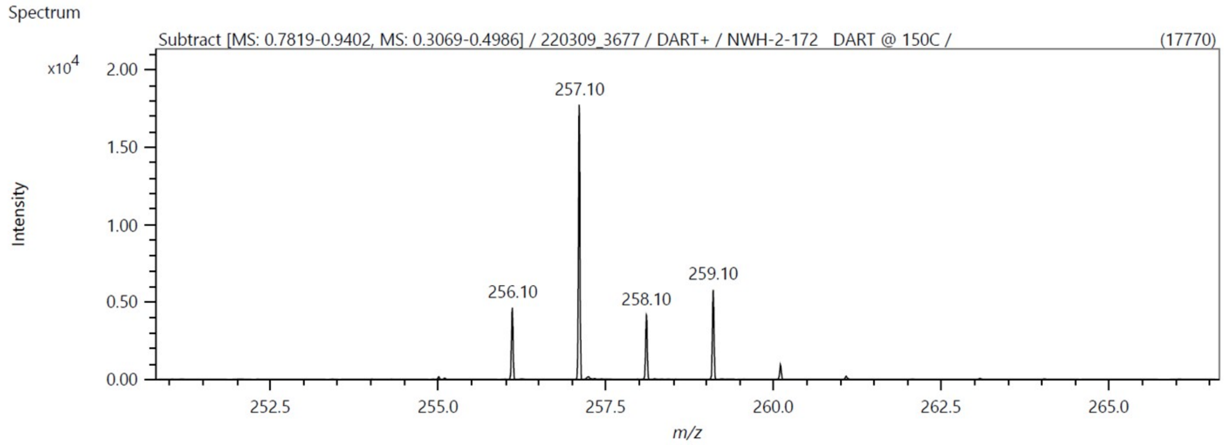

Elemental Composition

Parameters		Elements Set 1:						
Tolerance:	±10.00 mDa	Symbol	C	H	O	N	B	Cl
Electron:	Odd/Even	Min	0	0	0	0	1	0
Charge:	+1	Max	100	100	20	10	1	1
DBE:	-1.5 - 100.0							

Results

Mass	Intensity	Formula	Calculated Mass	Mass Difference [mDa]	Mass Difference [ppm]	DBE
257.10183	17770.37	C4 H10 B N10 O3	257.10249	-0.66	-2.56	5.5
		C5 H16 B N3 O8	257.10250	-0.66	-2.58	0.0
		C3 H14 B N6 O7	257.10115	0.68	2.64	0.5
		C14 H15 B N2 Cl	257.10113	0.70	2.72	8.5
		C17 H12 B N O	257.10065	1.19	4.62	13.0
		C6 H12 B N7 O4	257.10383	-2.00	-7.78	5.0

Figure S34: HRMS of carboxyborindolizine **3h** (DART+)

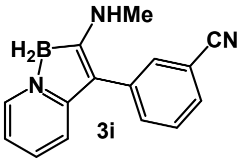

Aryl borindolizine 3i: 65% yield. ^{1}H NMR (600 MHz, $CDCl_3$) δ 8.02 (d, *J* = 6.2 Hz, 1H), 7.62 (s, 1H), 7.57 (m, 1H), 7.52 (m, 2H), 7.39 (m, 1H), 6.92 (d, *J* = 8.7 Hz, 1H), 6.58 (t, *J* = 1.2 Hz, 1H), 5.36 (s, 1H), 2.98 (d, *J* = 5.4 Hz, 3H). ^{13}C NMR (126 MHz, $CDCl_3$) δ 200.7, 162.0, 142.5, 138.0, 133.6, 132.5, 130.2, 129.4, 119.0, 113.5, 113.3, 112.3, 103.6, 34.2. ^{11}B NMR (192 MHz, $CDCl_3$) δ -12.47. HRMS m/z (DART+) calcd for $C_{15}H_{15}BN_3$ ([M] + H) 248.1535 found 248.13515.

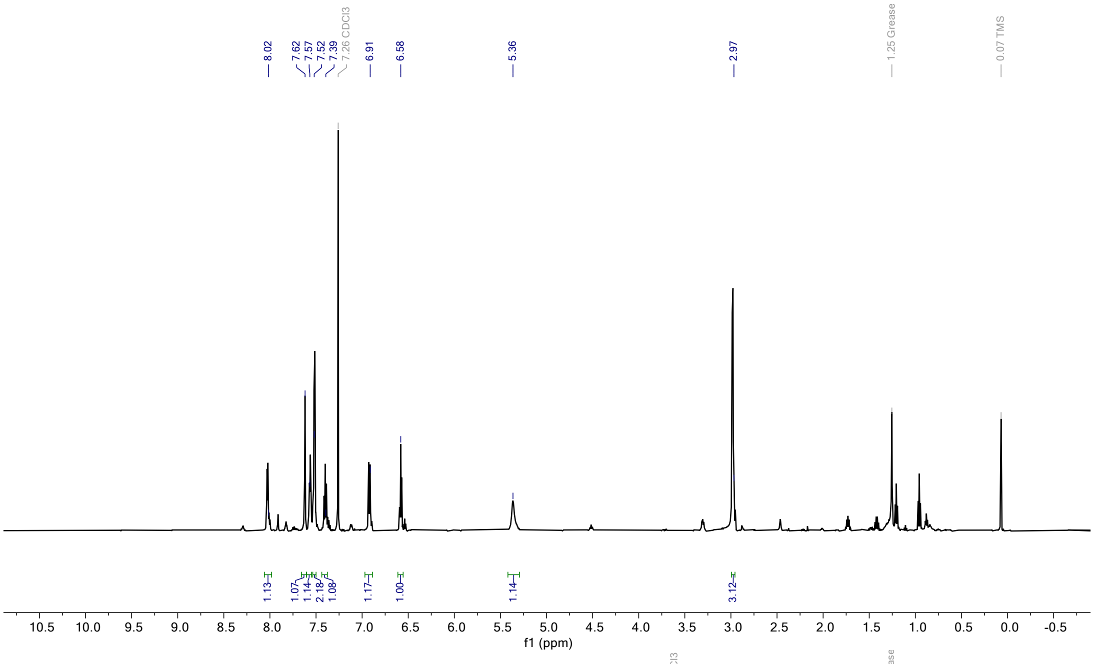
8.02
7.62
7.57
7.52
7.39
7.26 CDCl3
6.91
6.58
5.36
2.97
1.25 Grease
0.07 TMS
1.13
1.07
1.14
2.18
1.08
1.17
1.00
1.14
3.12
f1 (ppm)

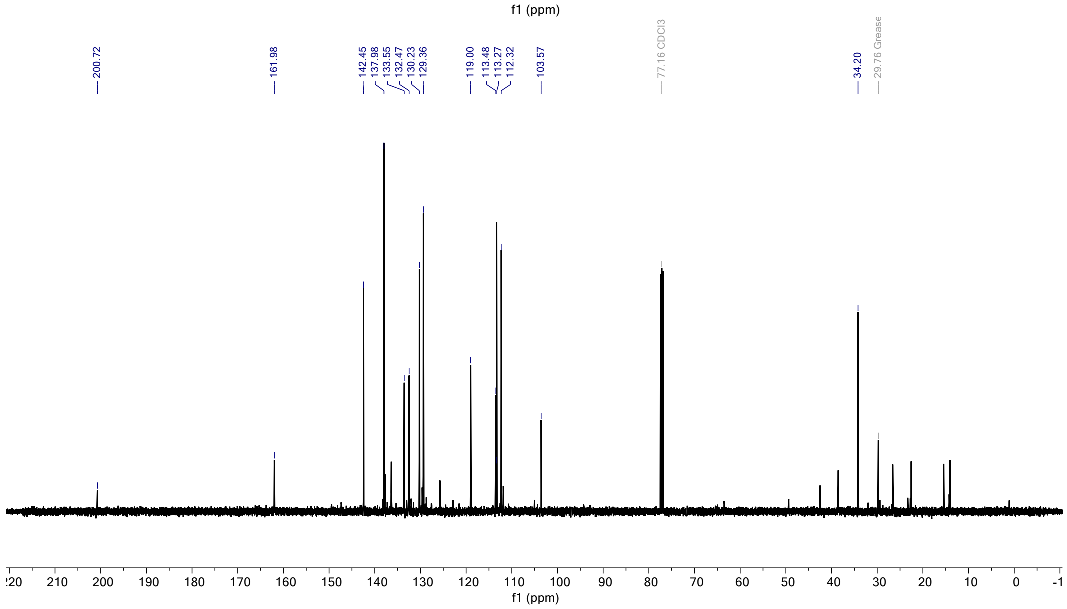
200.72
161.98
142.45
137.98
133.55
132.47
130.23
129.36
119.00
113.48
113.27
112.32
103.57
77.16 CDCl3
34.20
29.76 Grease
f1 (ppm)

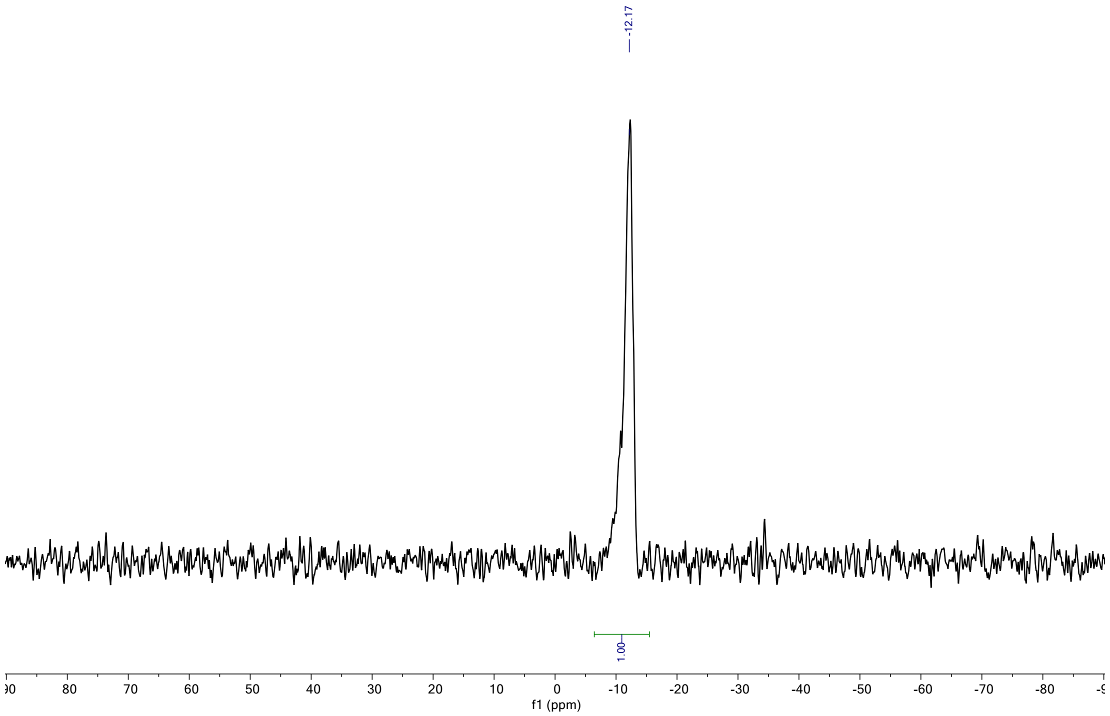

Figure S35: ^{1}H, ^{13}C and ^{11}B NMR of arylborindolizine **3i** in $CDCl_3$ (298K)

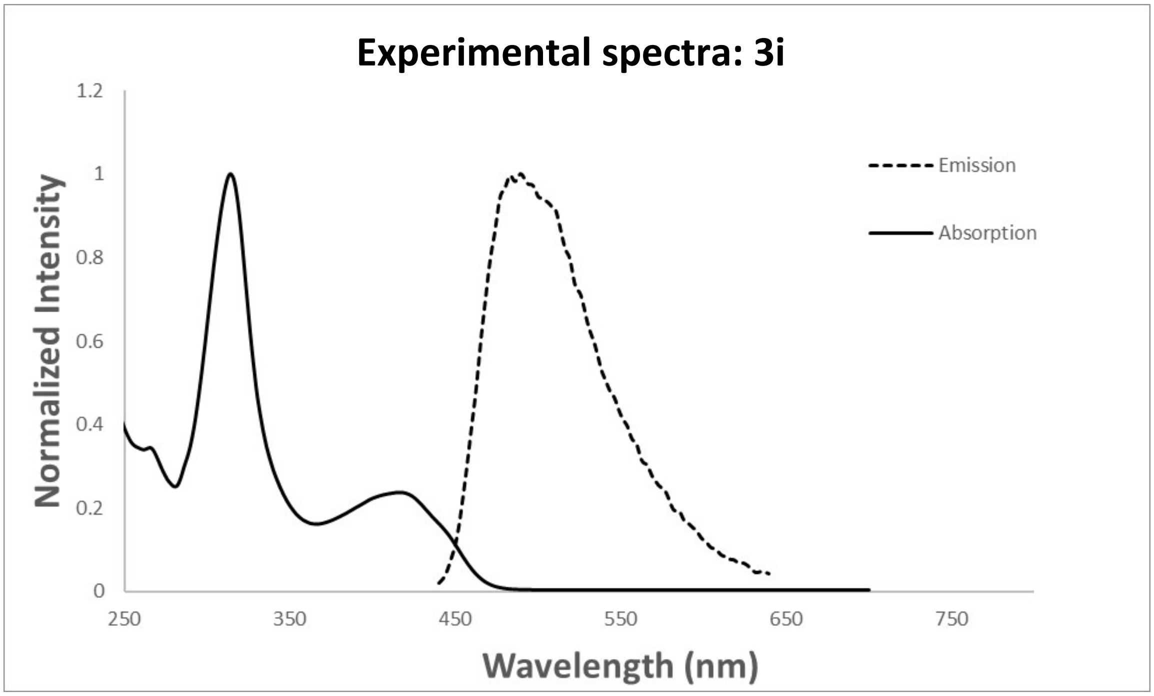

Figure S36: Overlayed absorption and emission spectra of borindolizine **3i** in dichloromethane.

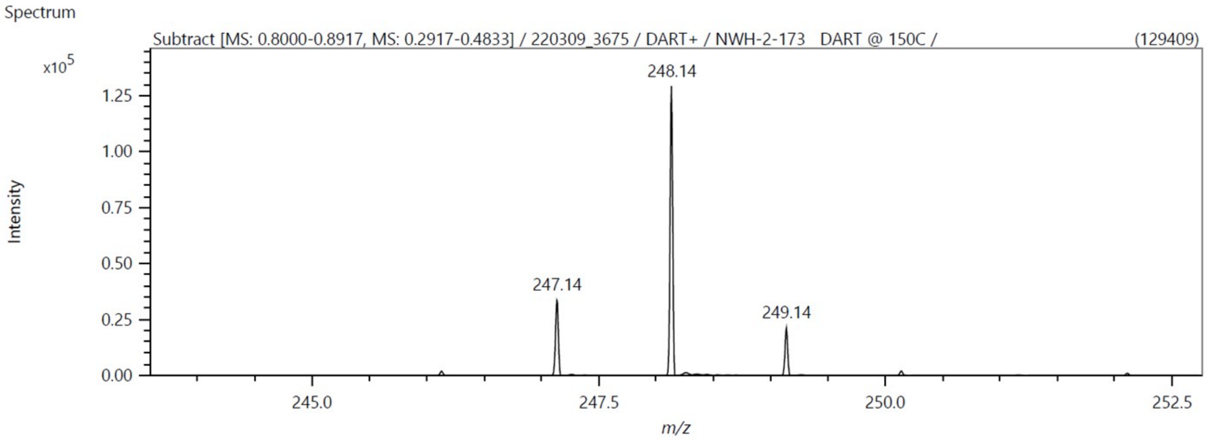

Elemental Composition

Parameters	
Tolerance:	±10.00 mDa
Electron:	Odd/Even
Charge:	+1
DBE:	-1.5 - 100.0

Elements Set 1:

Symbol	C	H	O	N	B	F
Min	0	0	0	0	1	0
Max	100	100	20	10	1	1

Results

Mass	Intensity	Formula	Calculated Mass	Mass Difference [mDa]	Mass Difference [ppm]	DBE
248.13515	129408.58	C10 H14 B N6 F	248.13515	0.00	-0.02	7.0
		C15 H15 B N3	248.13535	-0.20	-0.82	10.5
		C9 H18 B N2 O4 F	248.13382	1.33	5.37	2.0
		C12 H16 B N3 O F	248.13650	-1.35	-5.43	6.5
		C17 H17 B O	248.13670	-1.55	-6.23	10.0
		C12 H17 B N2 O3	248.13267	2.48	9.98	6.0

Figure S37: HRMS of carboxyborindolizine **3h** (DART+)

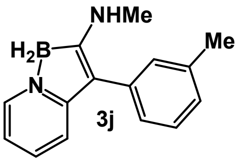

Aryl borindolizine 3j; yield = 68%; ^{1}H NMR (600 MHz, $CDCl_3$) δ 7.98 (d, J = 5.7 Hz, 1H), 7.33 (t, J = 2.2 Hz, 2H), 7.15 (m, 2H), 7.07 (d, J = 7.5 Hz, 1H), 6.97 (d, J = 8.6 Hz, 1H), 6.49 (t, J = 6.3 Hz, 1H), 5.39 (s, 1H), 2.97 (d, J = 4.9 Hz, 3H), 2.39 (s, 3H). ^{13}C NMR (151 MHz, $CDCl_3$) δ 162.8, 142.3, 139.0, 137.5, 134.7, 129.7, 129.3, 126.9, 126.2, 114.1, 111.5, 106.1, 34.1, 21.6. ^{11}B NMR (192 MHz, $CDCl_3$) δ -12.58. HRMS m/z (DART+) calcd for $C_{15}H_{18}BN_2$ ([M] + H) 237.15576 found 237.15639.

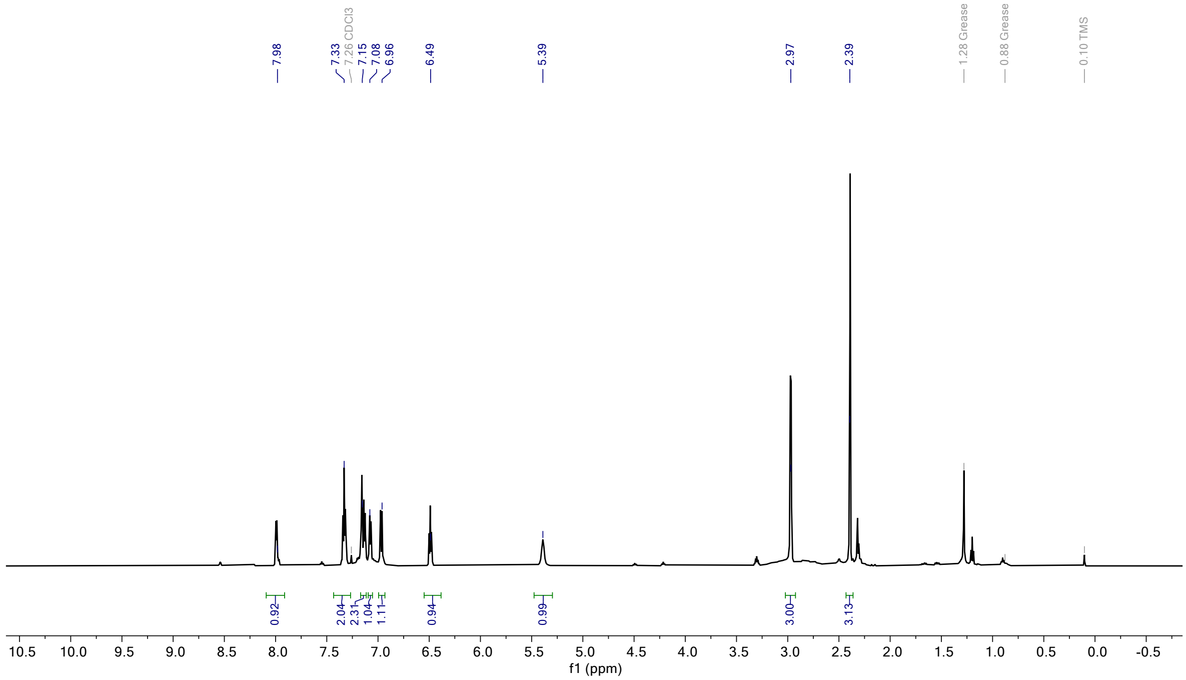
7.98
7.33
7.26 CDCl3
7.15
7.08
6.96
6.49
5.39
2.97
2.39
1.28 Grease
0.88 Grease
0.10 TMS
0.92
2.04
2.31
1.04
1.11
0.94
0.99
3.00
3.13
f1 (ppm)

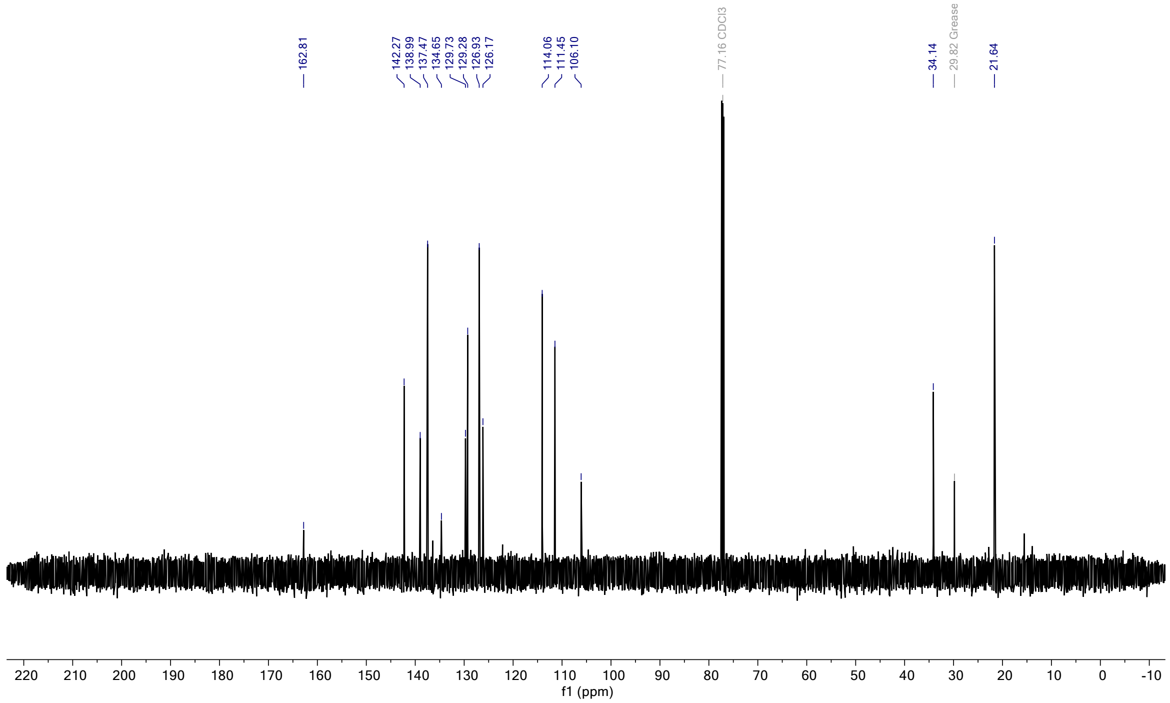
162.81
142.27
138.99
137.47
134.65
129.73
129.28
126.93
126.17
114.06
111.45
106.10
77.16 CDCl3
34.14
29.82 Grease
21.64
f1 (ppm)

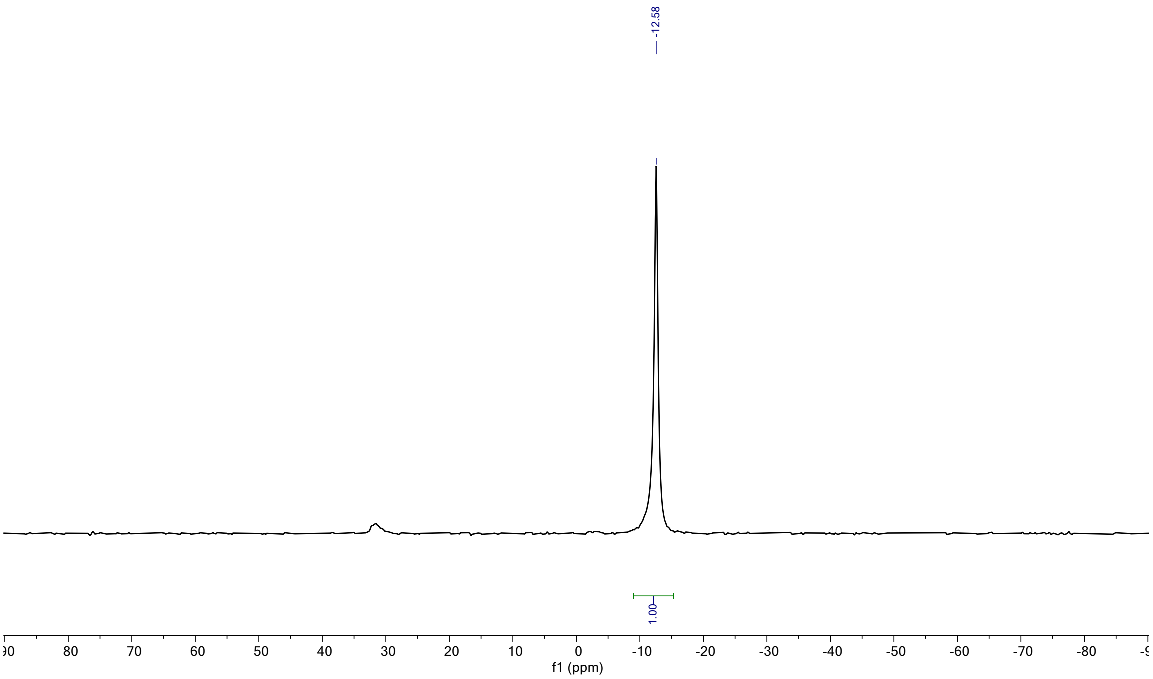

Figure S38: ^{1}H, ^{13}C and ^{11}B NMR of arylborindolizine **3j** in $CDCl_3$ (298K)

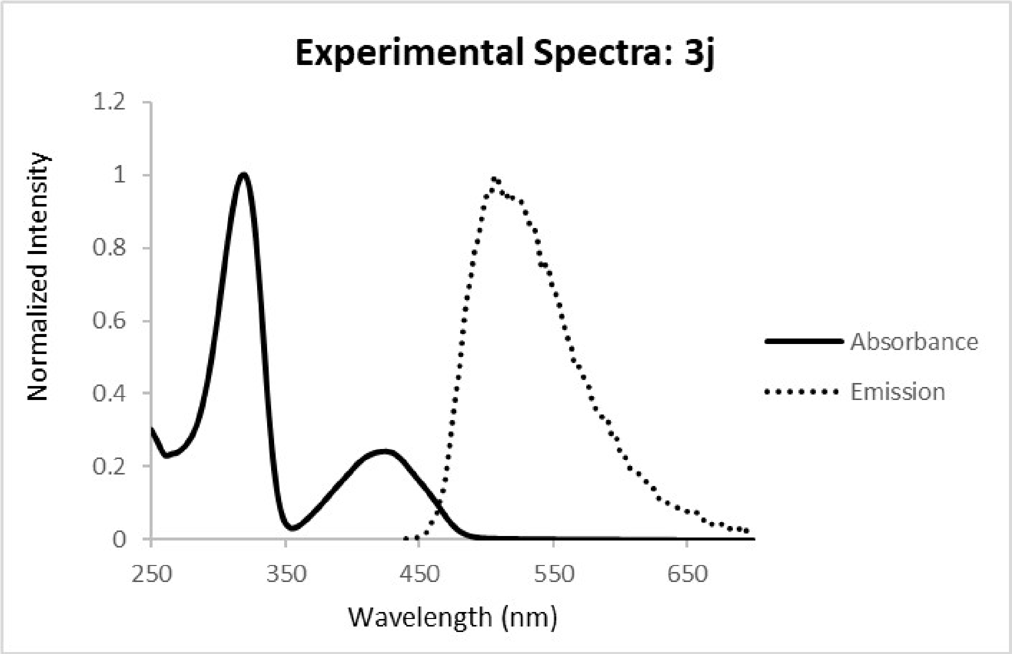

Figure S39: Overlayed absorption and emission spectra of borindolizine **3j** in dichloromethane.

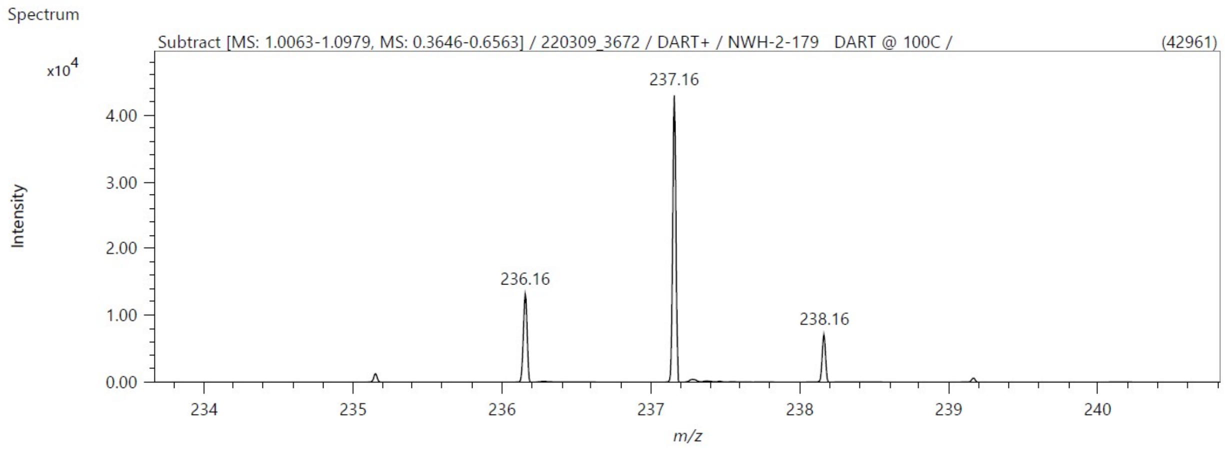

Elemental Composition

Parameters		Elements Set 1:						
Tolerance:	±10.00 mDa	Symbol	C	H	O	N	B	S
Electron:	Odd/Even	Min	0	0	0	0	1	0
Charge:	+1	Max	100	100	20	10	1	1
DBE:	-1.5 - 100.0							

Results

Mass	Intensity	Formula	Calculated Mass	Mass Difference [mDa]	Mass Difference [ppm]	DBE
237.15639	42960.53	C9 H24 B N O3 S	237.15645	-0.06	-0.26	-1.0
		C15 H18 B N2	237.15576	0.63	2.65	8.5
		C7 H22 B N4 O2 S	237.15510	1.28	5.40	-0.5
		C4 H18 B N8 O3	237.15894	-2.56	-10.79	0.5
		C5 H20 B N7 O S	237.15376	2.62	11.07	0.0
		C12 H22 B N2 S	237.15913	-2.74	-11.56	3.5

Figure S40: HRMS of carboxyborindolizine **3i** (DART+)

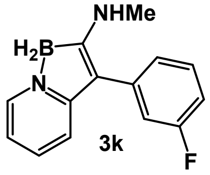

Aryl borindolizine 3k 46%, yellow solid. ^{1}H NMR (500 MHz, $CDCl_3$) δ 8.00 (d, *J* = 5.9 Hz, 1H), 7.37 (m, 2H), 7.10 (d, *J* = 1.3 Hz, 1H), 7.03 (d, *J* = 1.5 Hz, 1H), 6.99 (d, *J* = 8.6 Hz, 1H), 6.93 (td, *J* = 8.5, 1.0 Hz, 1H), 6.53 (t, *J* = 1.2 Hz, 1H), 5.43 (s, 1H), 2.97 (d, *J* = 4.9 Hz, 3H). ^{13}C NMR (126 MHz, $CDCl_3$) δ 163.8 (d, *J* = 246.5 Hz), 162.4, 142.4, 137.8, 137.2 (d, *J* = 8.0 Hz), 130.9 (d, *J* = 8.8 Hz), 124.7, 115.6 (d, *J* = 20.2 Hz), 113.9, 112.9 (d, *J* = 21.2 Hz), 111.9, 104.8, 34.2. ^{11}B NMR (160 MHz, $CDCl_3$) δ -12.47. ^{19}F NMR (377 MHz, $CDCl_3$) δ -112.39 (q, *J* = 8.8 Hz). HRMS m/z (DART+) calcd for $C_{14}H_{15}BN_2F$ ([M] + H) 241.13068 found 241.13034.

CRAPT ^{13}C NMR was used to characterize 3k to determine which carbon atoms were missing from each ^{13}C spectra. Note: ^{13}C NMR peak splitting by the ^{19}F nucleus is observed.

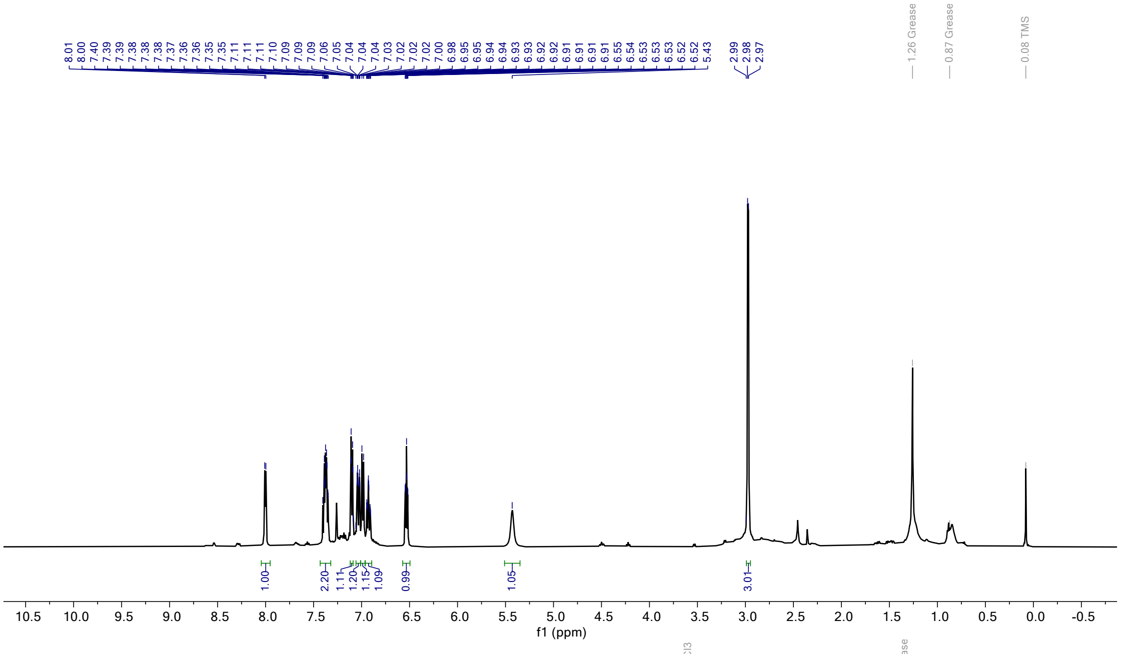

8.01 8.00 7.40 7.39 7.39 7.38 7.38 7.38 7.37 7.36 7.36 7.35 7.35 7.11 7.11 7.11 7.10 7.09 7.09 7.09 7.06 7.05 7.04 7.04 7.04 7.03 7.02 7.02 7.02 7.00 6.98 6.95 6.95 6.94 6.94 6.93 6.93 6.92 6.92 6.91 6.91 6.91 6.91 6.55 6.54 6.53 6.53 6.53 6.52 6.52 5.43
2.99 2.98 2.97
1.26 Grease
0.87 Grease
0.08 TMS
1.00 2.20 1.11 1.20 1.15 1.09 0.99 1.05 3.01
f1 (ppm)

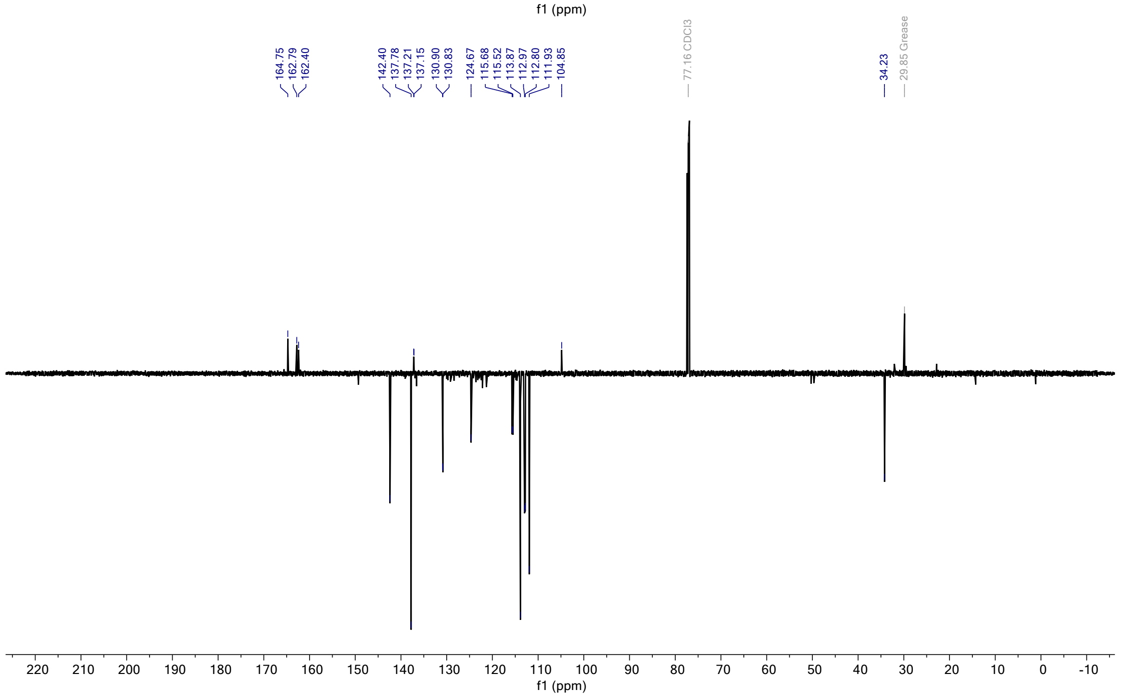

164.75 162.79 162.40
142.40 137.78 137.21 137.15 130.90 130.83
124.67
115.68 115.52 113.87 112.97 112.80 111.93
104.85
77.16 CDCl3
34.23
29.85 Grease
f1 (ppm)

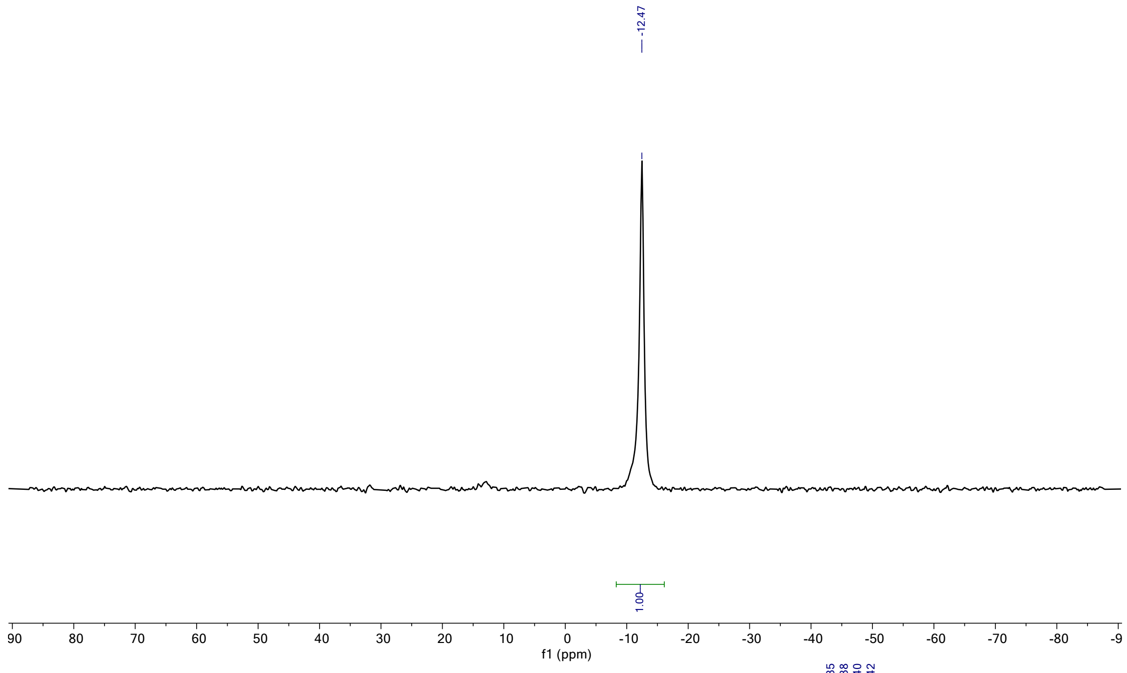

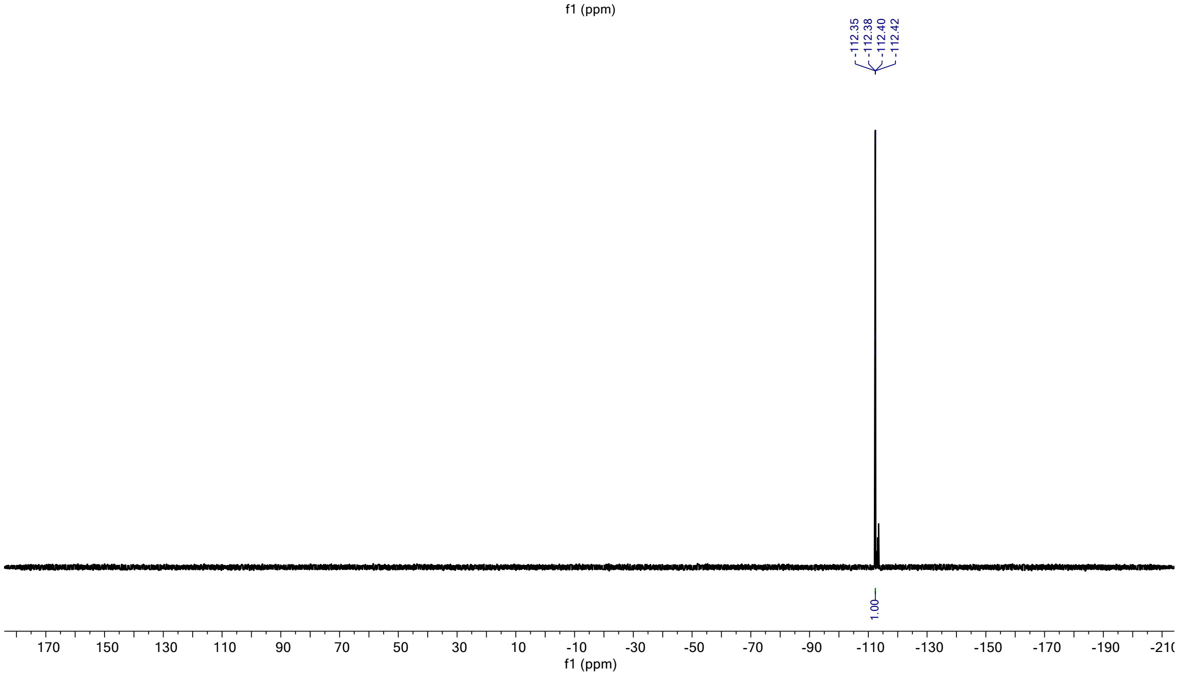

Figure S41: ^{1}H, ^{13}C, ^{11}B and 19F NMR of arylborindolizine **3k** in $CDCl_3$ (298K)

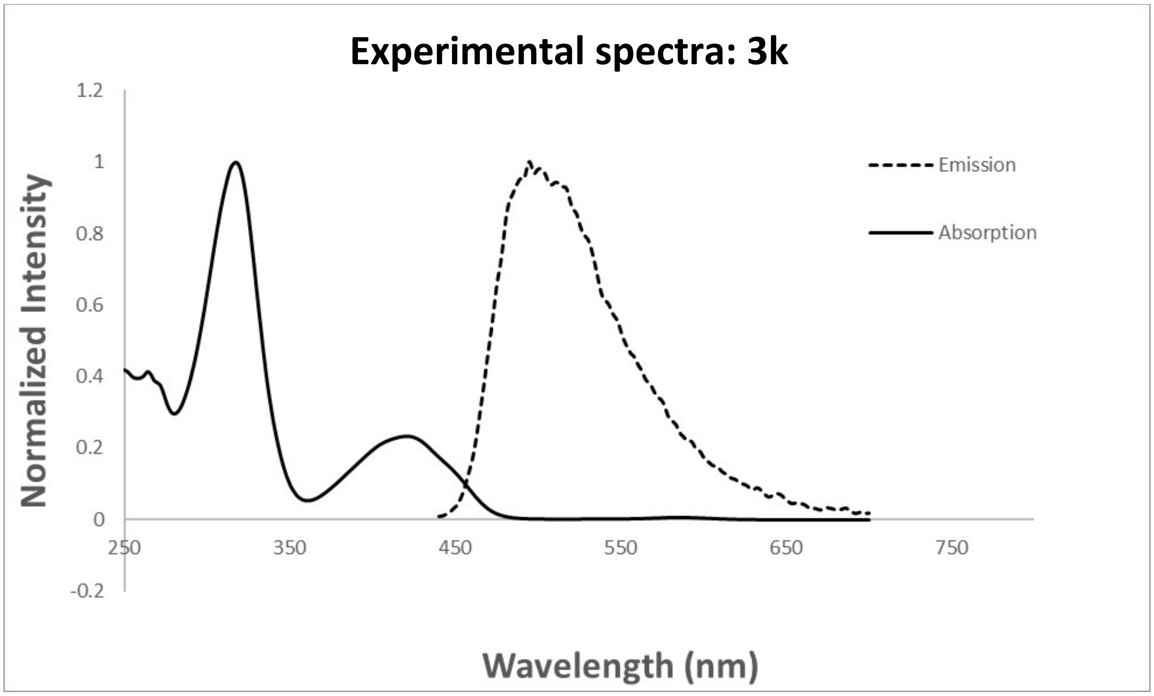

Figure S42: Overlayed absorption and emission spectra of borindolizine **3k** in dichloromethane.

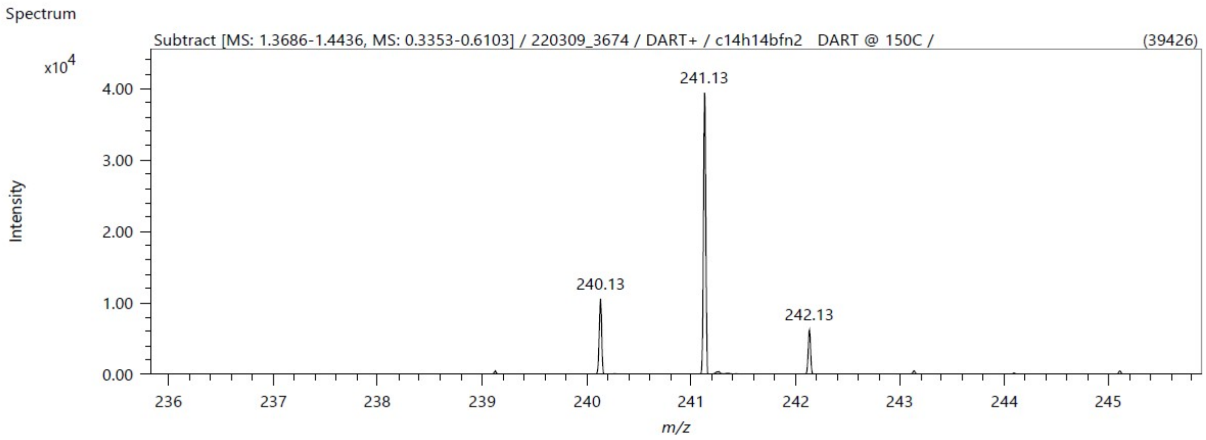

Elemental Composition

Parameters	
Tolerance:	±10.00 mDa
Electron:	Odd/Even
Charge:	+1
DBE:	-1.5 - 100.0

Elements Set 1:

Symbol	C	H	O	N	B	F
Min	0	0	0	0	1	0
Max	100	100	20	10	1	1

Results

Mass	Intensity	Formula	Calculated Mass	Mass Difference [mDa]	Mass Difference [ppm]	DBE
241.13034	39426.40	C3 H16 B N7 O5	241.13005	0.30	1.23	0.0
		C14 H15 B N2 F	241.13068	-0.34	-1.41	8.5
		C5 H18 B N4 O6	241.13139	-1.05	-4.34	-0.5
		C H14 B N10 O4	241.12871	1.64	6.80	0.5
		C11 H17 B N O3 F	241.12800	2.34	9.71	4.0
		C6 H14 B N8 O2	241.13273	-2.38	-9.89	4.5

Figure S43: HRMS of carboxyborindolizine **3k** (DART+)

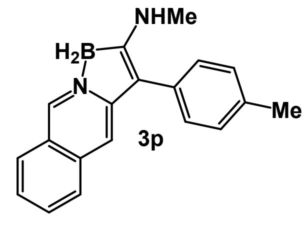

Isoquinolyl borindolizine 3p: 61% yield, red solid.^{1}H NMR (400 MHz, $CDCl_3$) δ 8.80 (s, 1H), 7.64 (d, *J* = 1.1 Hz, 1H), 7.41 (m, 2H), 7.31 (q, *J* = 8.2 Hz, 4H), 7.14 (ddd, *J* = 8.1, 6.1, 1.7 Hz, 1H), 7.08 (s, 1H), 5.17 (s, 1H), 2.99 (d, *J* = 4.9 Hz, 3H), 2.41 (s, 3H). ^{13}C NMR (126 MHz, $CDCl_3$) δ 158.5, 146.4, 139.2, 135.8, 132.2, 132.2, 130.2, 129.1, 128.6, 125.6, 123.5, 122.3, 106.0, 105.0, 34.1, 21.4.^{11}B NMR (192 MHz, $CDCl_3$) δ -11.97 HRMS m/z (ESI) calcd for $C_{14}H_{15}BN_2F$ ([M] + H) 286.16413 found 286.16408.

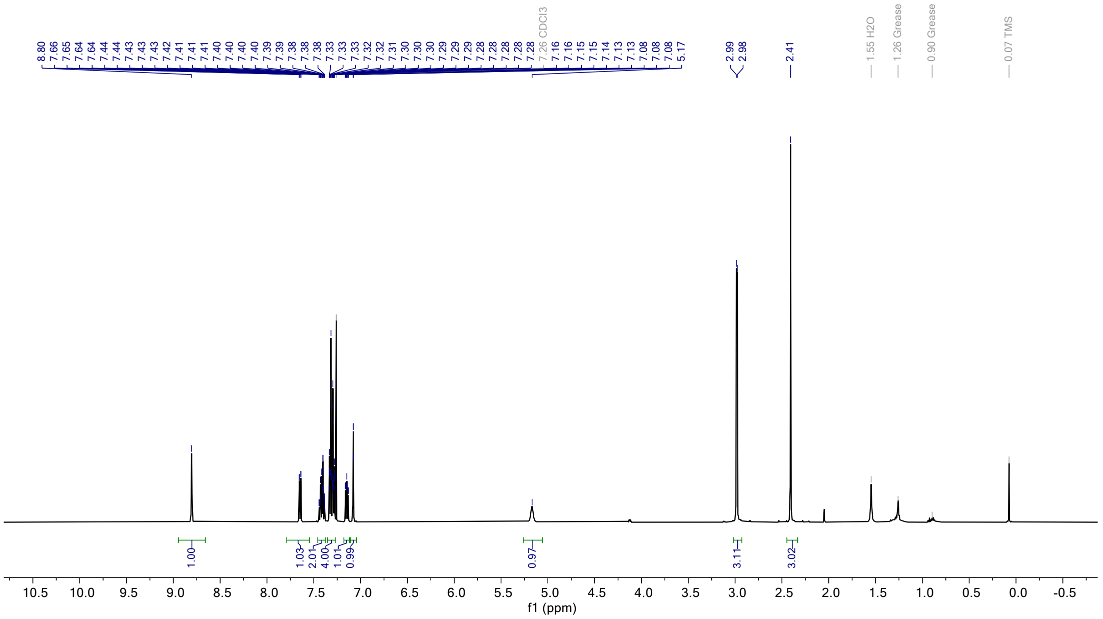

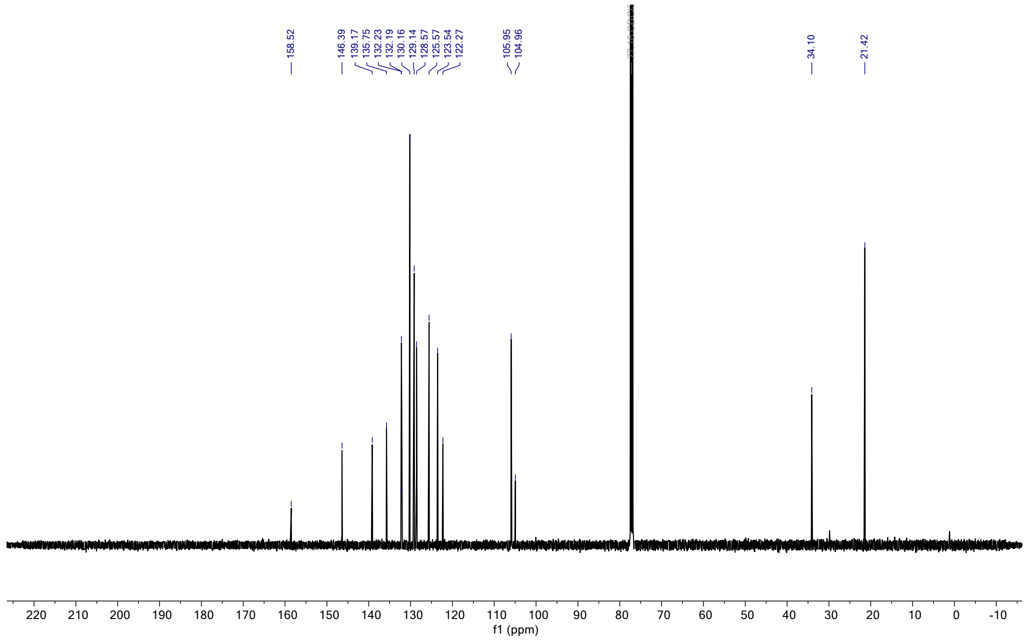

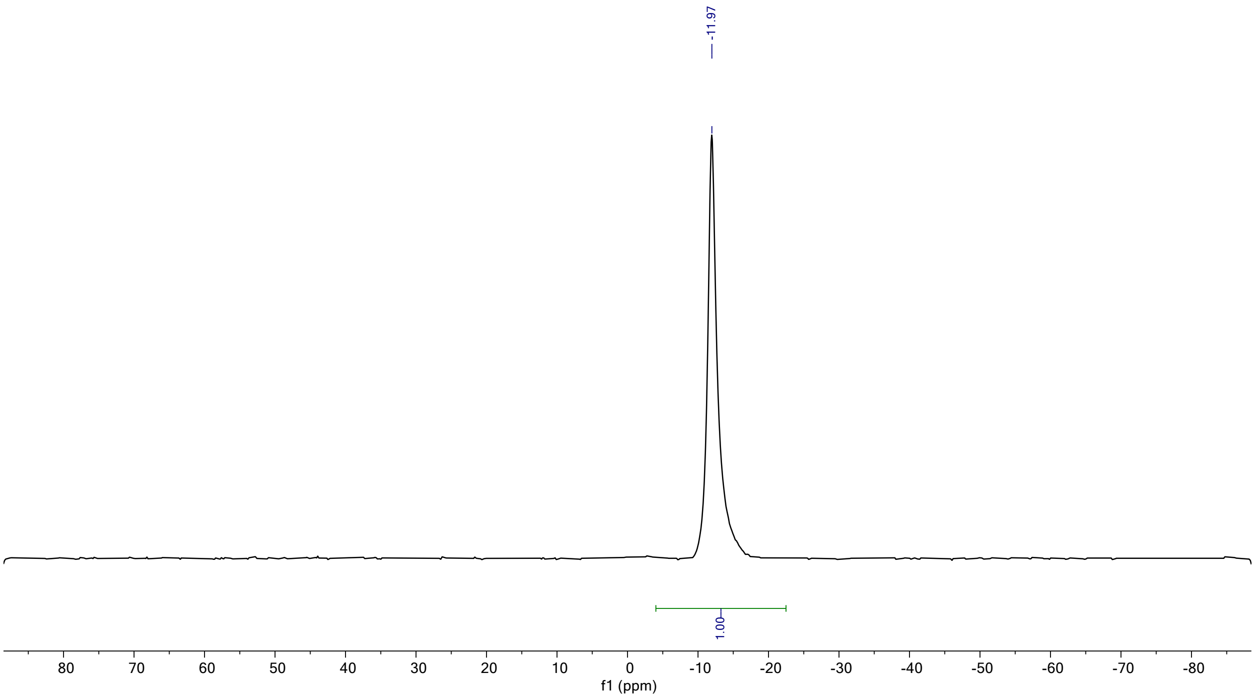

Figure S44: ^{1}H, ^{13}C and ^{11}B NMR of arylborindolizine **3p** in $CDCl_3$ (298K)

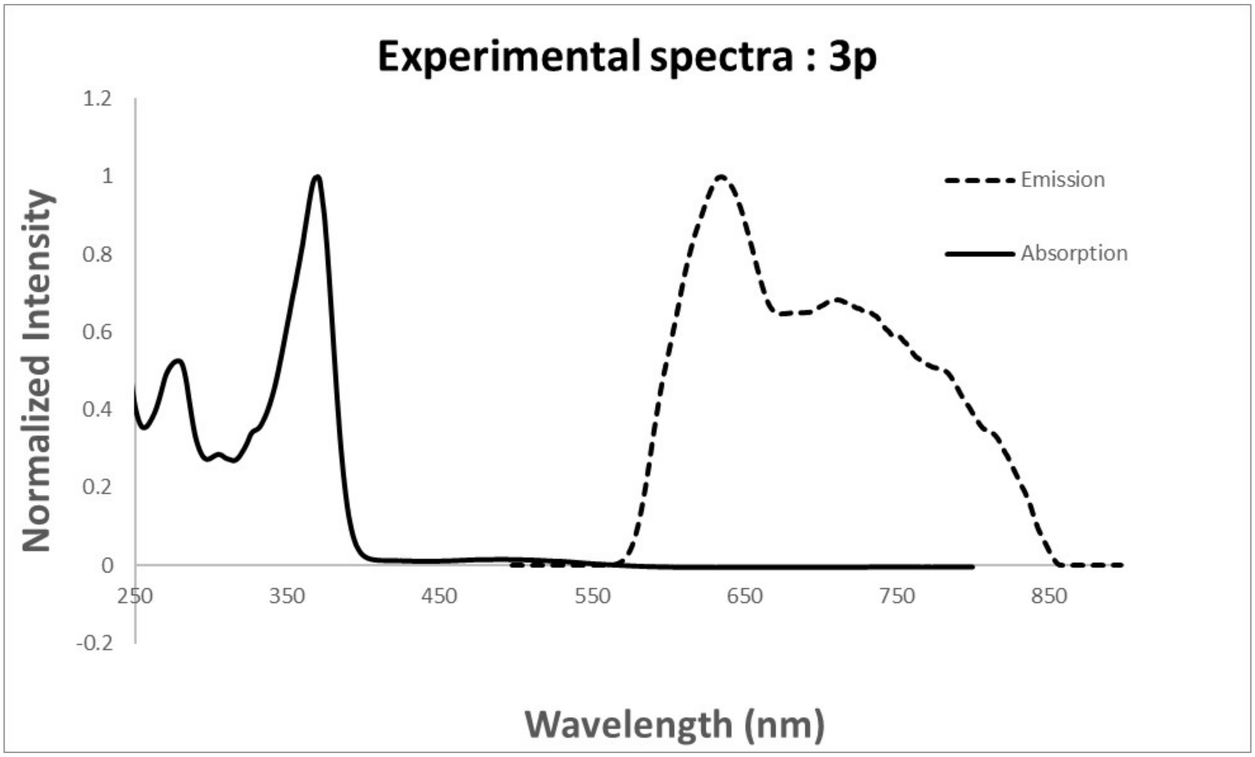

Figure S45: Overlayed absorption and emission spectra of borindolizine **3p** in dichloromethane.

Summarized Photophysical Data of Synthesized Compounds 3a – 3p

Table S1 contains absorption and emission maxima, stokes shift values, and quantum yields of selected compounds (**3a – 3p**) in dichloromethane.

Borindolizine	$\lambda_{(max, abs)}$ (nm)	$\lambda_{(max, em)}$ (nm)	Stokes Shift (cm^{-1})	Quantum Yield
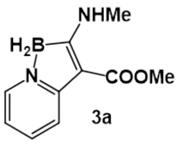 3a	289, 373	431	3,608	0.538
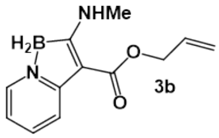 3b	288, 372	438	4,051	0.508
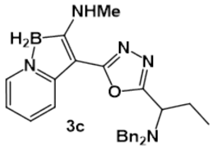 3c	306, 398	459	3,339	0.498
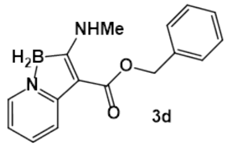 3d	286, 371	438	4,123	0.604
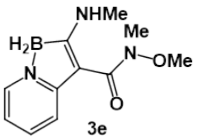 3e	290, 374	442	4,062	0.490
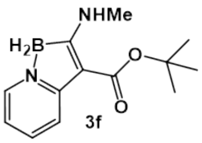 3f	289, 375	445	4,195	0.481

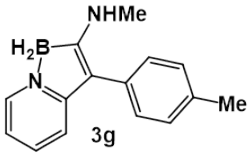 3g	320, 426	519	4,206	0.141
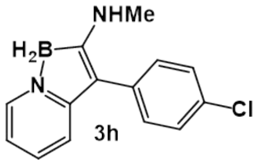 3h	316, 420	507	4,086	0.262
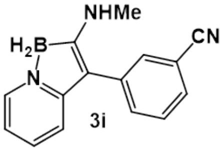 3i	314, 416	489	3,589	0.270
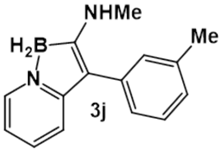 3j	320, 424	507	3,861	0.168
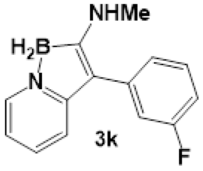 3k	318, 420	495	3,608	0.192
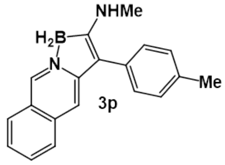 3p	278, 370	635	11,279	Unmeasurable

Table S1: Absorption maxima, emission maxima, Stokes shift and quantum yields of synthesized compounds (**3a – 3p**). The emission of **3p** was not detectable at absorbance ranges below 0.1.

Quantum Yield Measurements

Fluorescent quantum yield was determined using quinine hemisulfate monohydrate as a reference standard (Φ = 0.546). To acquire fluorescent quantum yields, borindolizines were dissolved in a given solvent and UV-Vis spectra were recorded. The absorbance values at the $\lambda_{abs,max}$ were recorded, and then fluorescent emission spectra were acquired using the same samples. This was repeated for a minimum of four times to establish a relationship between an absorbance range of ~ 0.09-0.01 and the integrated fluorescent emission intensities. Relative fluorescent yields were then calculated using this data and the equation:

$$\Phi_{sample} = \Phi_{reference} \times \frac{slope_{sample}}{slope_{reference}} \times (\frac{n_{sample}}{n_{reference}})^2$$

where the sample refers to the borindolizine sample, the reference refers to the quinine sulfate standard, the slopes refer to the slope of the relationship between emission peak integration and absorbance, and n refers to the refractive index of the solvent that the compound being measured was dissolved in. The quantum yields for each compound are tabulated below:

3a Quantum Yield (dichloromethane)

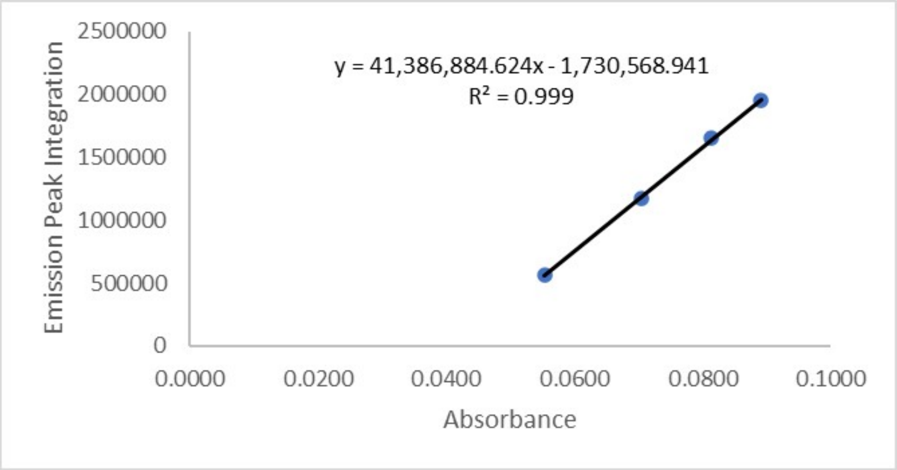

Φ = 0.538 in dichloromethane, calculated using quinine sulfate standard in 0.5 M H_2SO_4. Excitation wavelength was 373 nm.

3a Quantum Yield (acetonitrile)

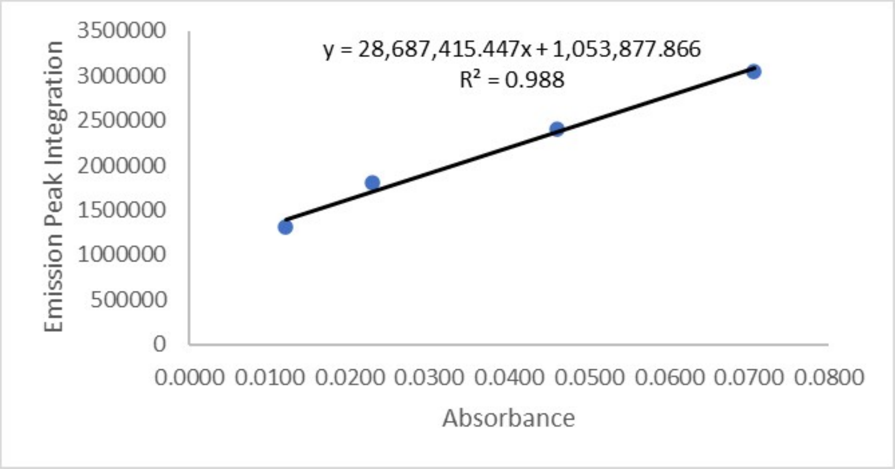

3a

Φ = 0.332 in acetonitrile, calculated using quinine sulfate standard in 0.5 M H_2SO_4. Excitation wavelength was 371 nm.

3b Quantum Yield (dichloromethane)

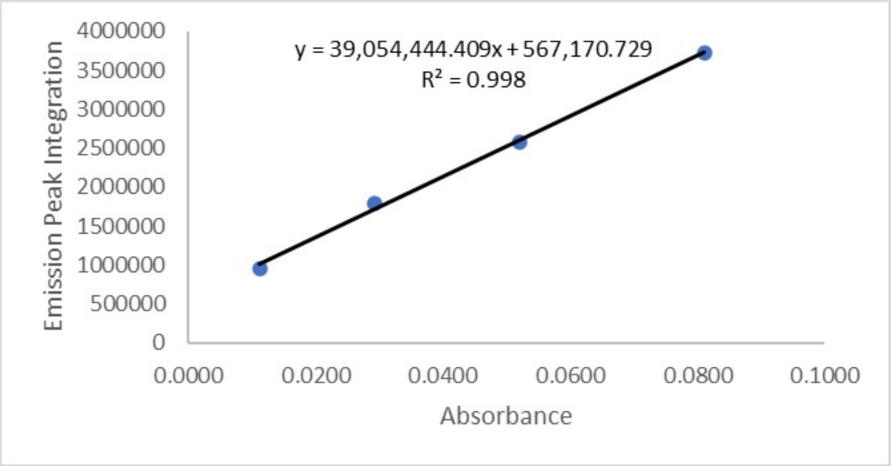

3b

Φ = 0.508 in dichloromethane, calculated using quinine sulfate standard in 0.5 M H_2SO_4. Excitation wavelength was 372 nm.

3b Quantum Yield (ethanol)

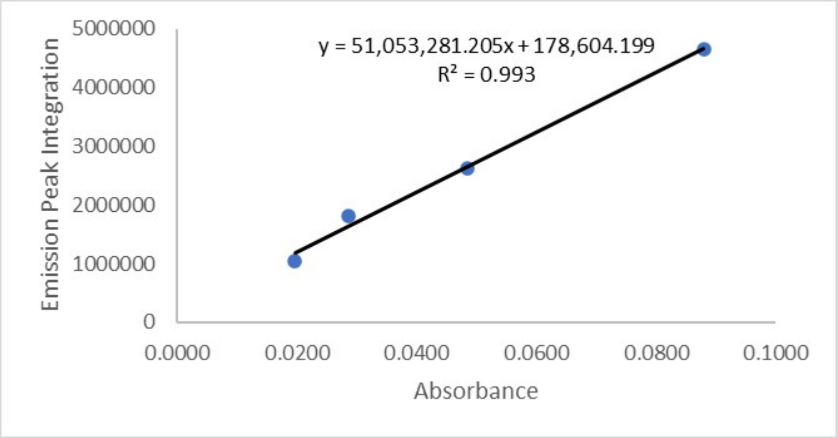

Φ = 0.607 in ethanol, calculated using quinine sulfate standard in 0.5 M H_2SO_4. Excitation wavelength was 368 nm.

3c Quantum Yield (dichloromethane)

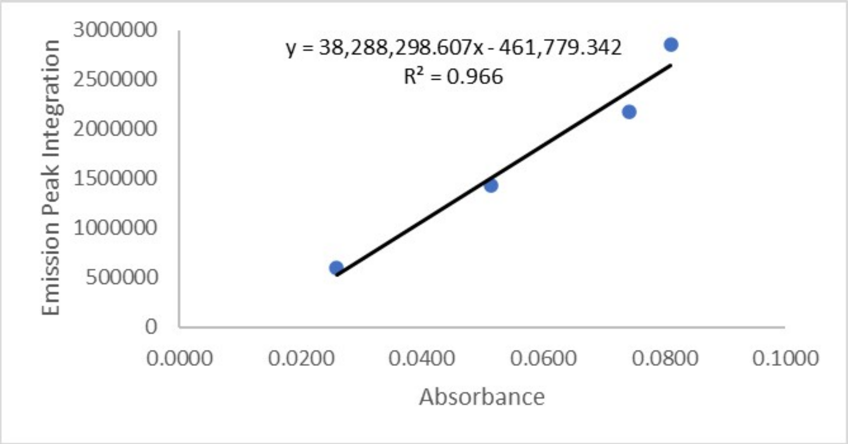

Φ = 0.498 in dichloromethane, calculated using quinine sulfate standard in 0.5 M H_2SO_4. Excitation wavelength was 398 nm.

3d Quantum Yield (dichloromethane)

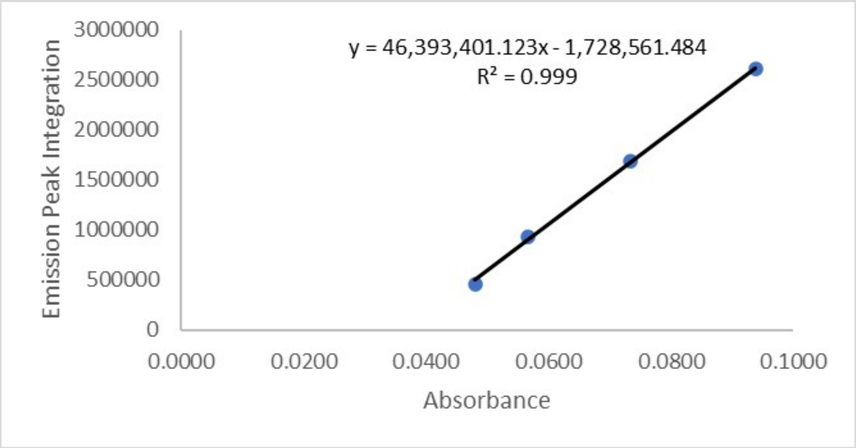

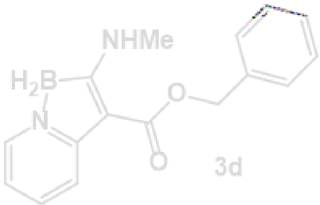

Φ = 0.604 in dichloromethane, calculated using quinine sulfate standard in 0.5 M H_2SO_4. Excitation wavelength was 371 nm.

3e Quantum Yield (dichloromethane)

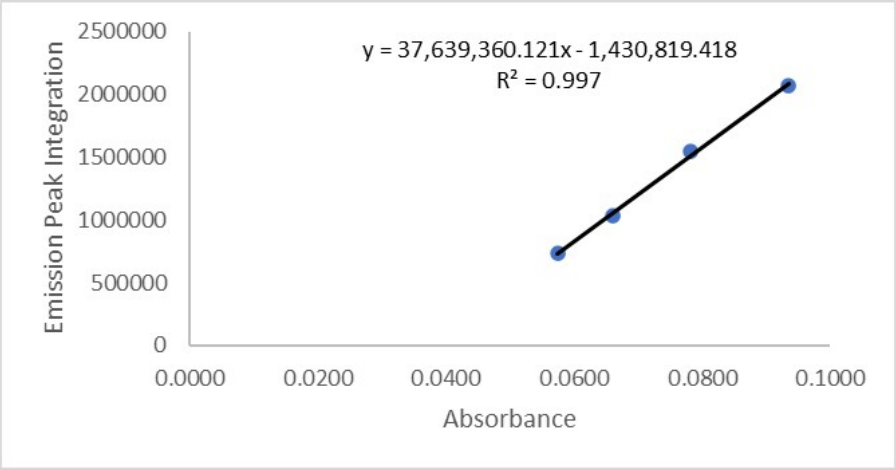

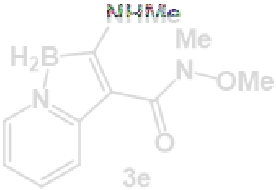

Φ = 0.490 in dichloromethane, calculated using quinine sulfate standard in 0.5 M

H_2SO_4. Excitation wavelength was 374 nm.

3f Quantum Yield (dichloromethane)

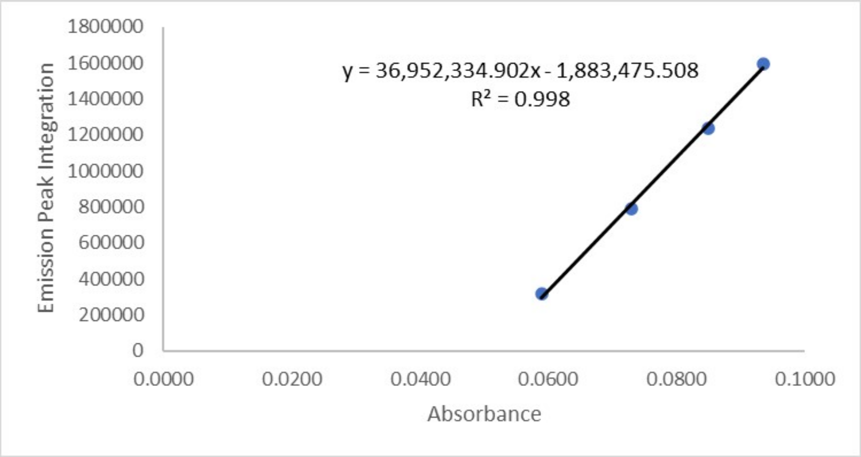

Φ = 0.481 in dichloromethane (black), calculated using quinine sulfate standard in 0.5 M H_2SO_4 (red). Excitation wavelength was 375 nm.

3g Quantum Yield (dichloromethane)

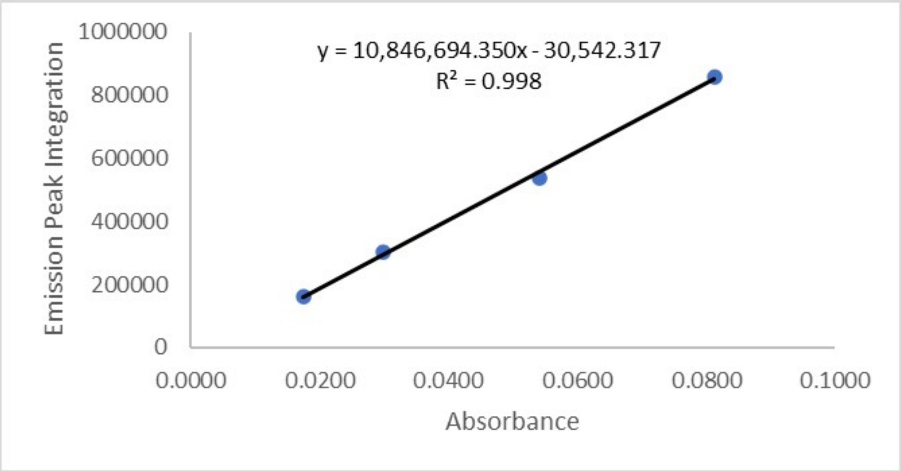

Φ = 0.141 in dichloromethane, calculated using quinine sulfate standard in 0.5 M H_2SO_4. Excitation wavelength was 426 nm.

3h Quantum Yield (dichloromethane)

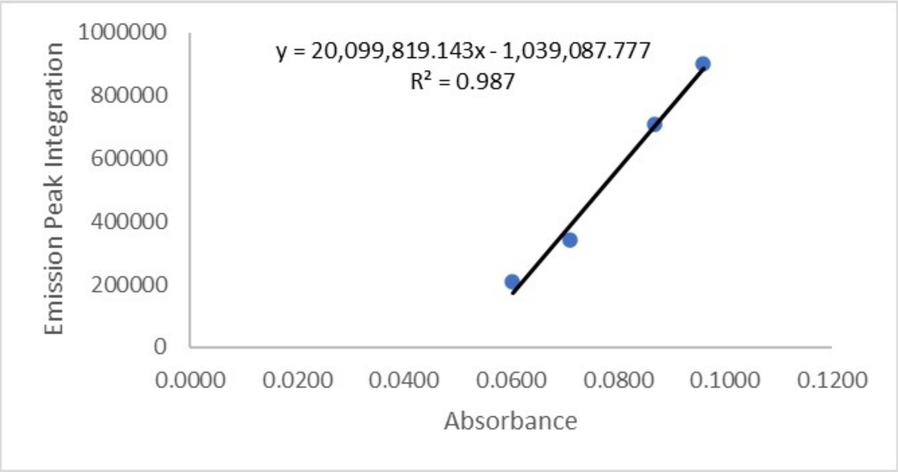

Φ = 0.262 in dichloromethane (black), calculated using quinine sulfate standard in 0.5 M H_2SO_4 (red). Excitation wavelength was 419 nm.

3i Quantum Yield (dichloromethane)

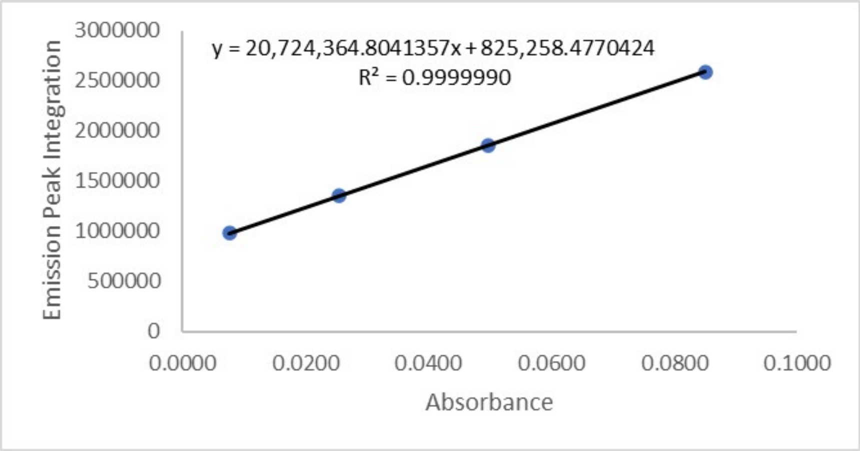

Φ = 0.270 in dichloromethane, calculated using quinine sulfate standard in 0.5 M H_2SO_4. Excitation wavelength was 415 nm.

3j Quantum Yield (dichloromethane)

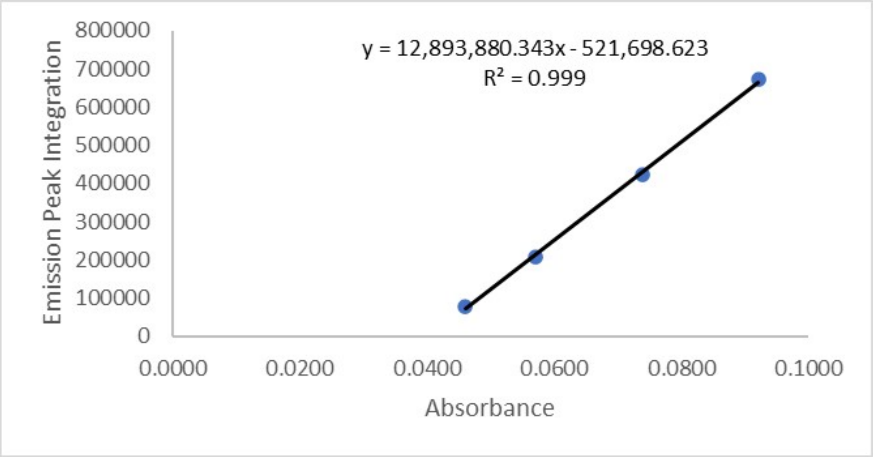

3j

Φ = 0.168 in dichloromethane, calculated using quinine sulfate standard in 0.5 M H_2SO_4. Excitation wavelength was 423 nm.

3k Quantum Yield (dichloromethane)

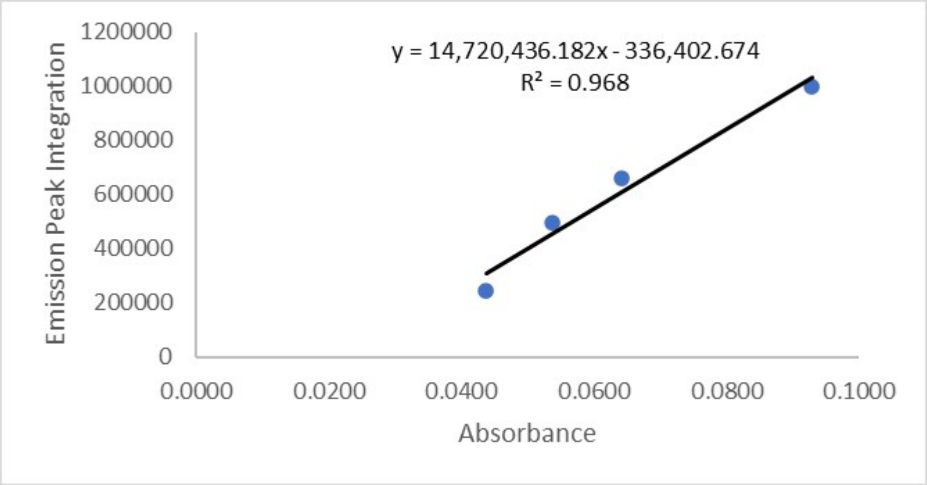

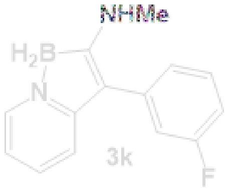

3k

Φ = 0.192 in dichloromethane, calculated using quinine sulfate standard in 0.5 M H_2SO_4. Excitation wavelength was 420 nm.

Computational Study: Spectral simulations

Initial conformer ensembles were generated using CREST[6] for every synthesized molecule in Table S1 **(3a – 3p)**, as well as a series of theoretical structures **(5a – 5l**, Figure S22). Conformer ensemble structures were then optimized using the GFN2-xTB semi-empirical method.[7] Hessian matrices were computed (using GFN2-xTB) for each structure to obtain normal modes of vibrations. Excited-state computations (TD-B3LYP/6-31G* basis, *c.f.* for benchmarking[8–12]) were performed using Q-Chem 5.2.[13] Excited-state gradients, projected over the normal modes, were then applied to compute absorption and emission spectra using the Vertical Gradient method, [14] utilizing software developed in the Aspuru-Guzik group. Spectral shapes were averaged using Boltzmann factors that relied on the ground state energies of the conformers at a temperature of 293.15 K. We note that no corrections were made to spectral shapes and intensities except for inhomogeneous broadening (500 cm^{-1}). Good agreement between experimental spectra measured in dichloromethane and simulated spectral shapes and intensities (using the refractive index of dichloromethane) was obtained.

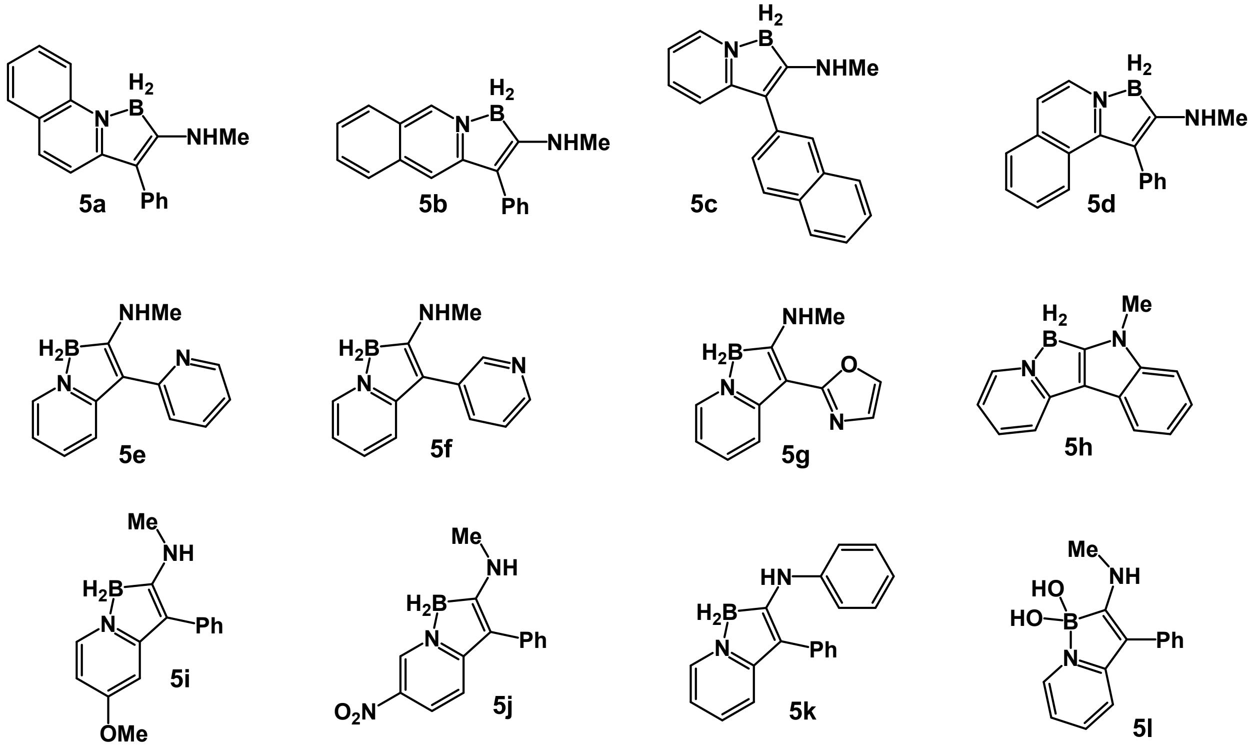

Figure S30: Library of computed structures aimed at modelling structure-emission relationships

The absorption and emission spectra of selected experimentally synthesized compounds (**3a** – **3p**) were simulated, and the maxima of all peaks observed was recorded.

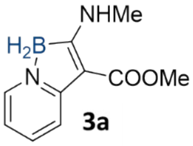

3a

	λmax (abs)	λmax (em)
computed	267 nm	403 nm
experimental	373 nm	431 nm

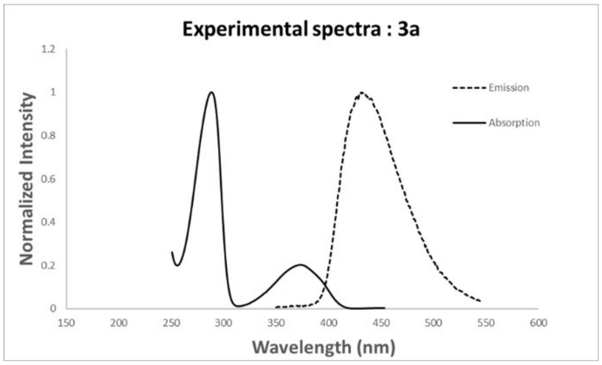

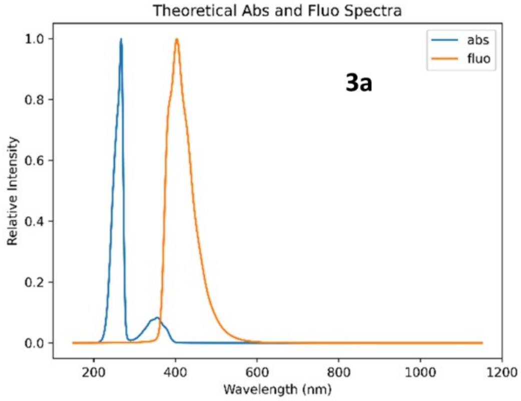

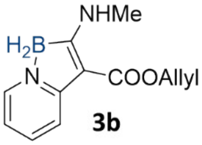

3b

	λmax (abs)	λmax (em)
computed	267 nm	403 nm
experimental	372 nm	438 nm

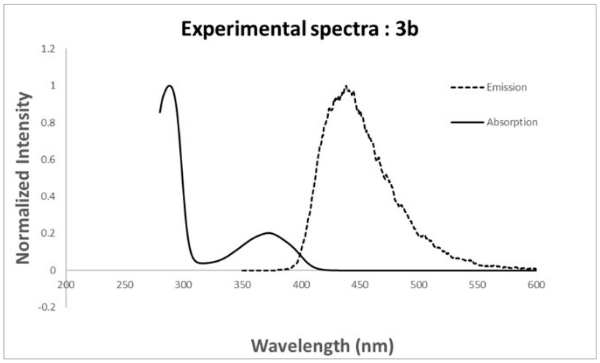

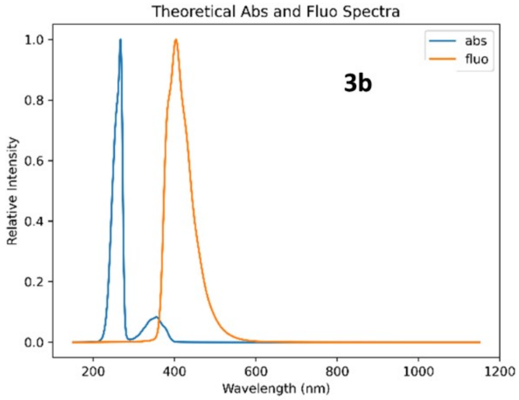

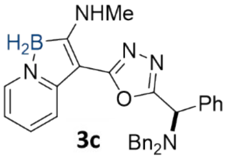

	λmax (abs)	λmax (em)
computed	316 nm	459 nm
experimental	398 nm	459 nm

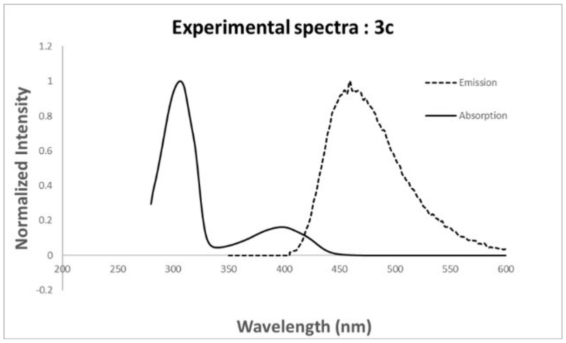

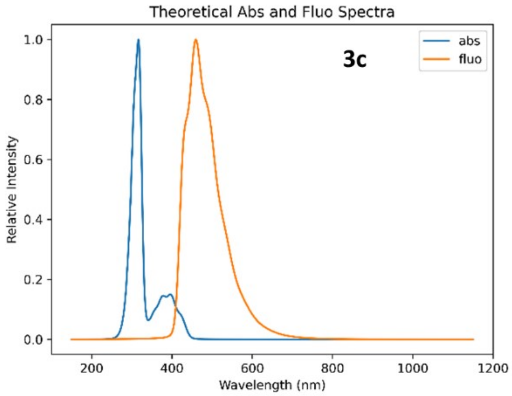

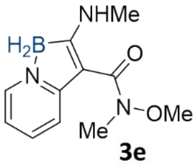

	λmax (abs)	λmax (em)
computed	263 nm	424 nm
experimental	374 nm	441 nm

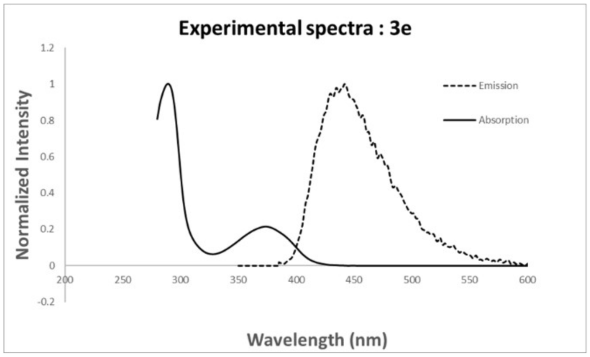

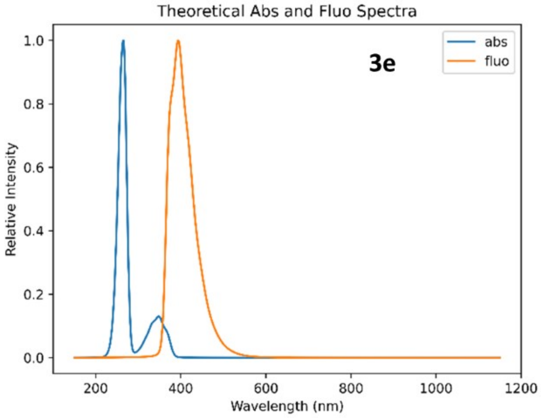

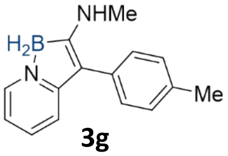

3g

	λmax (abs)	λmax (em)
computed	282 nm	475 nm
experimental	426 nm	519 nm

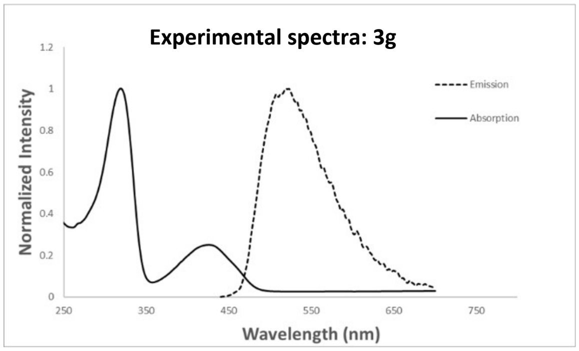

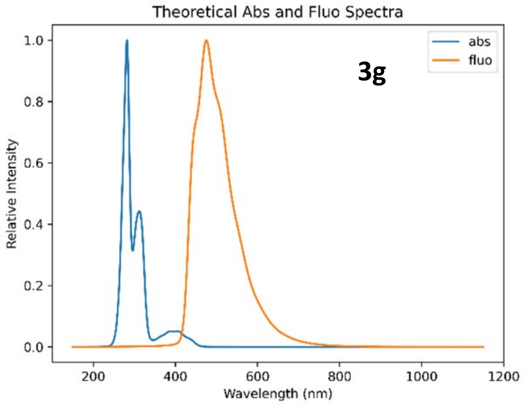

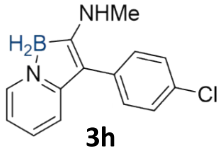

3h

	λmax (abs)	λmax (em)
computed	280 nm	464 nm
experimental	420 nm	507 nm

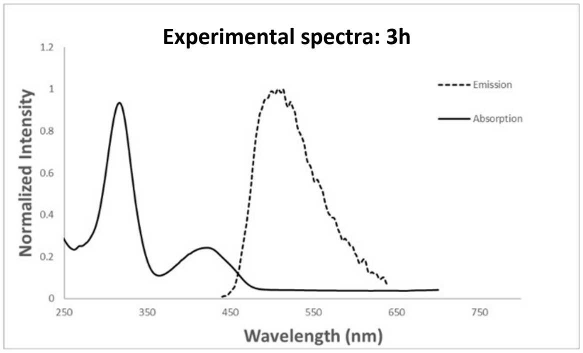

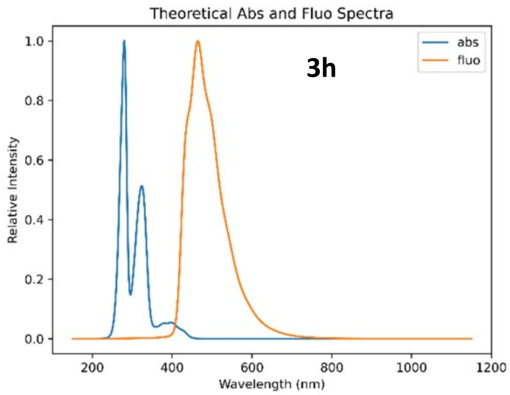

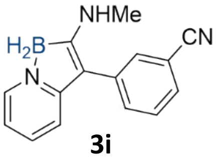

3i

	λmax (abs)	λmax (em)
computed	277 nm	477 nm
experimental	416 nm	489 nm

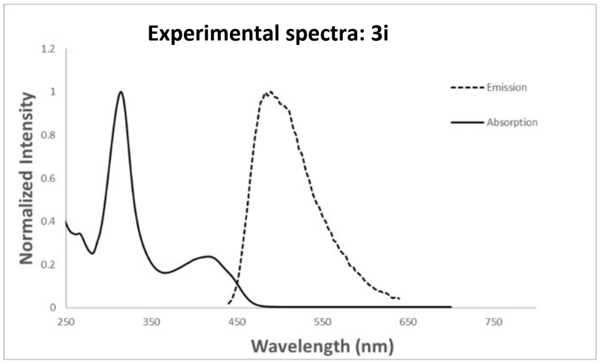

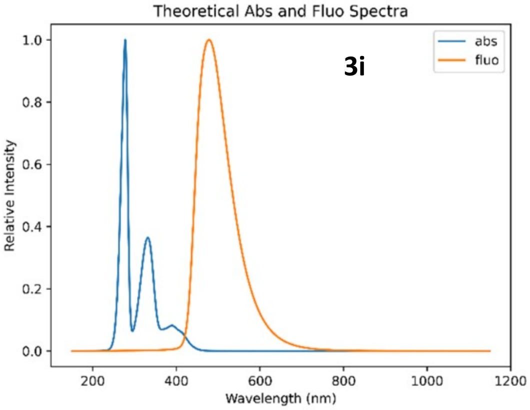

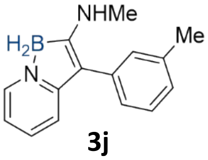

3j

	λmax (abs)	λmax (em)
computed	281 nm	472 nm
experimental	426 nm	489 nm

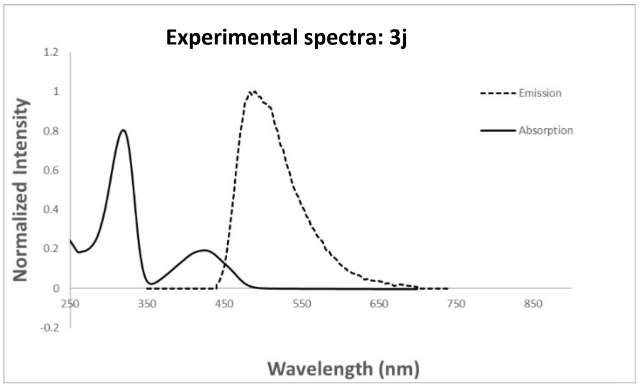

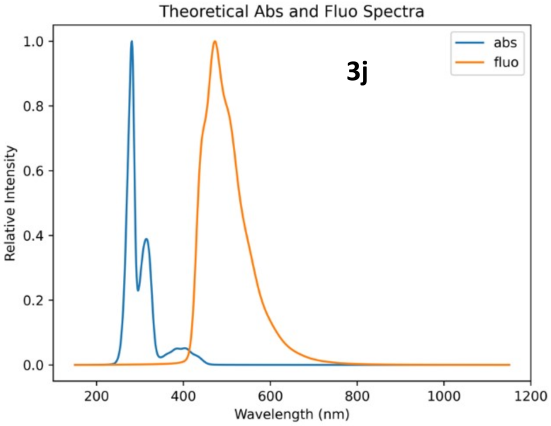

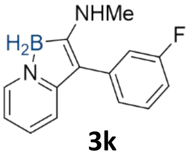

3k

	λmax (abs)	λmax (em)
computed	279 nm	462 nm
experimental	420 nm	495 nm

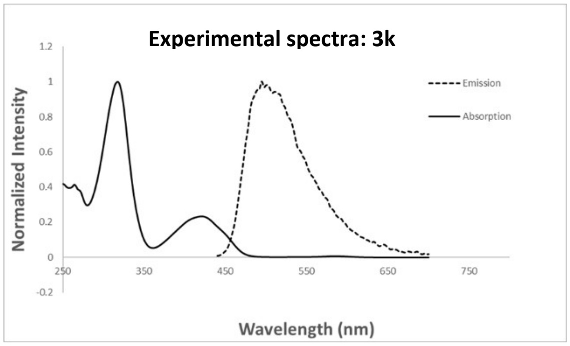

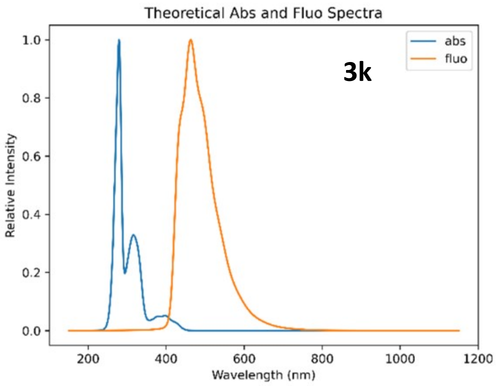

Compound	ems_max_exp (nm)	ems_max_theo (nm)
**3a**	431	403
**3b**	438	403
**3c**	459	459
**3e**	442	424
**3g**	519	475
**3h**	507	464
**3i**	489	477
**3j**	489	472
**3k**	495	462

Table S2: Compiled experimental and theoretical emission maxima

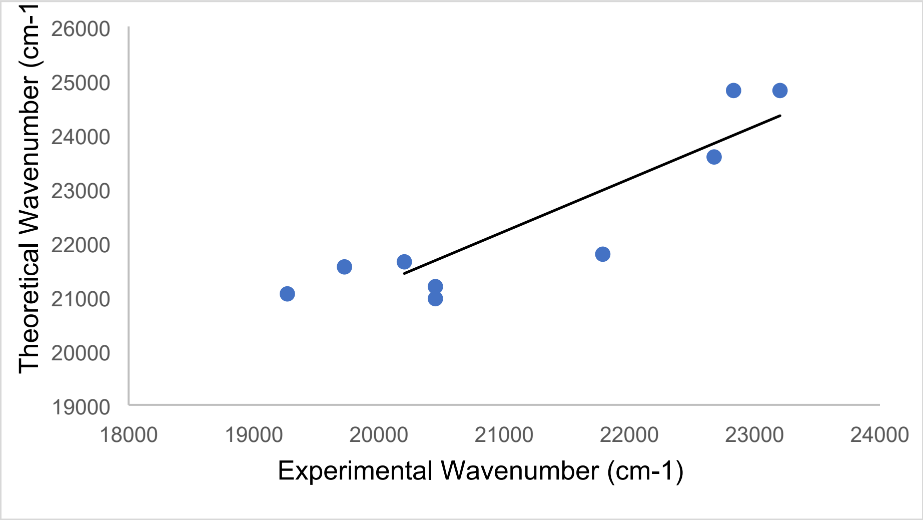

Figure S46: Linear fit for theoretical and experimental emission wavenumbers (cm^{-1})

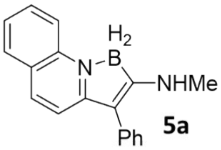

5a

	λmax (abs)	λmax (em)	HOMO-LUMO (eV)
computed	424 nm	492 nm	3.37

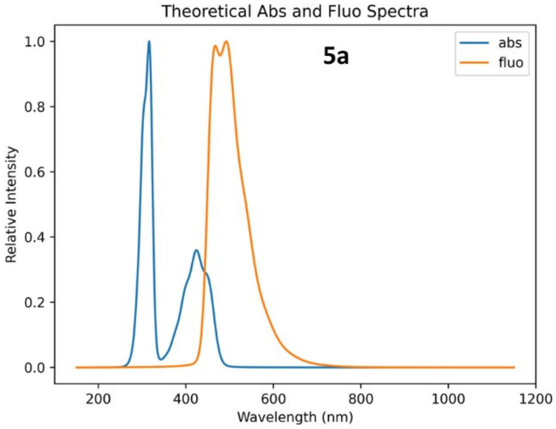

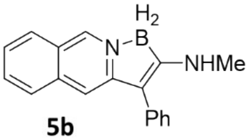

5b

	λmax (abs)	λmax (em)	HOMO-LUMO (eV)
computed	335 nm	646 nm	3.00

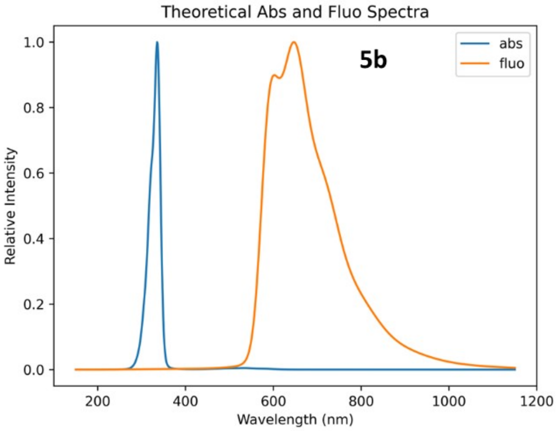

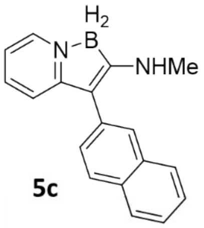

5c

	λmax (abs)	λmax (em)	HOMO-LUMO (eV)
computed	323 nm	423 nm	3.81

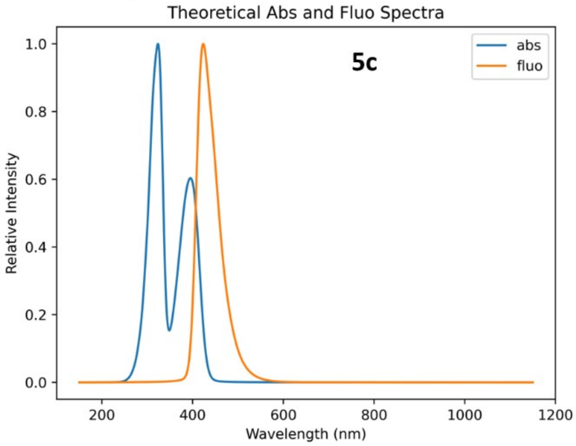

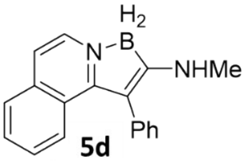

5d

	λmax (abs)	λmax (em)	HOMO-LUMO (eV)
computed	390 nm	412 nm	3.56

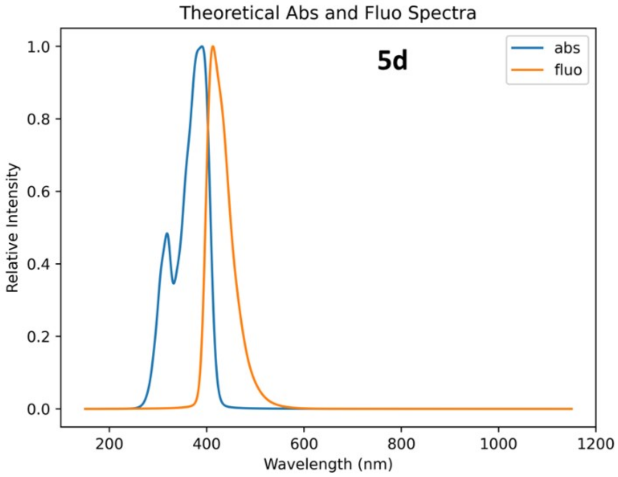

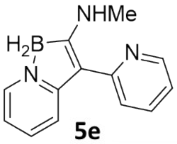

	λmax (abs)	λmax (em)	HOMO-LUMO (eV)
computed	312 nm	483 nm	3.76

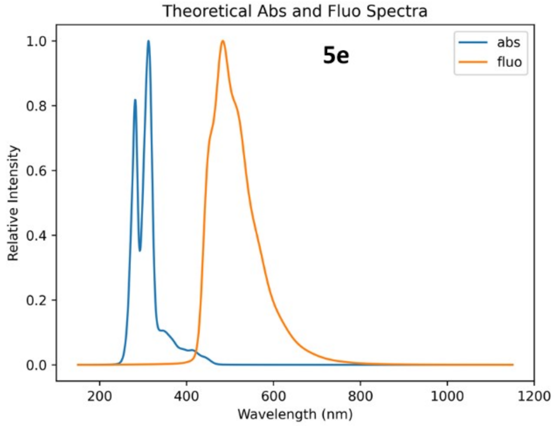

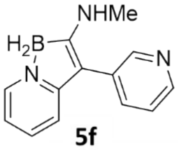

	λmax (abs)	λmax (em)	HOMO-LUMO (eV)
computed	280 nm	460 nm	-

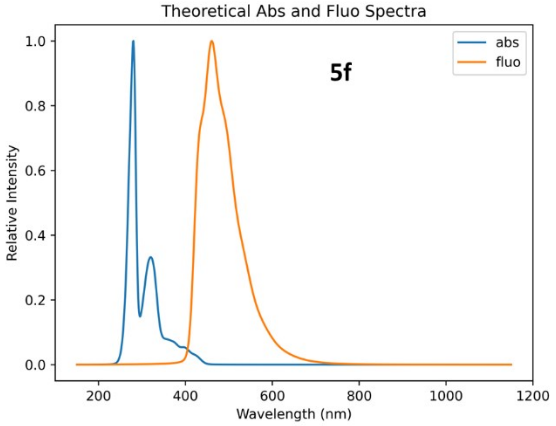

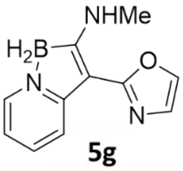

5g

	λmax (abs)	λmax (em)	HOMO-LUMO (eV)
computed	277 nm	482 nm	-

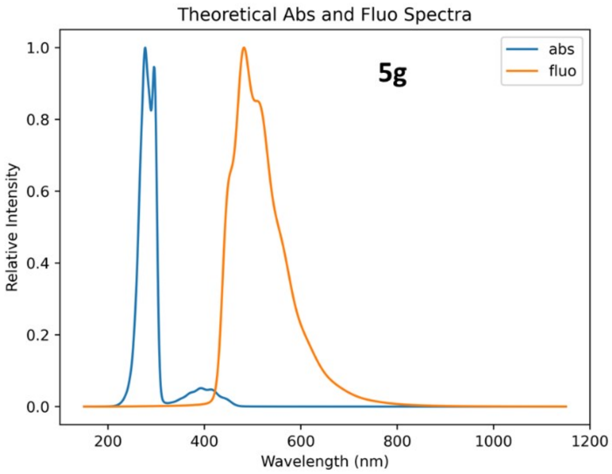

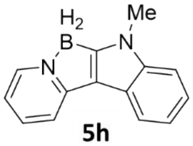

5h

	λmax (abs)	λmax (em)	HOMO-LUMO (eV)
computed	294 nm	457 nm	3.89

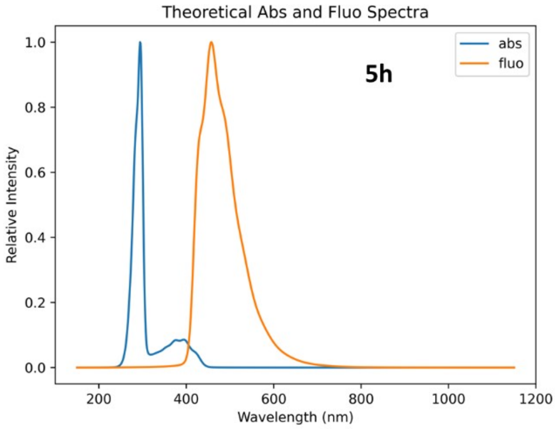

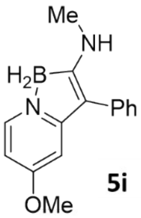

5i

	λmax (abs)	λmax (em)	HOMO-LUMO (eV)
computed	280 nm	422 nm	-

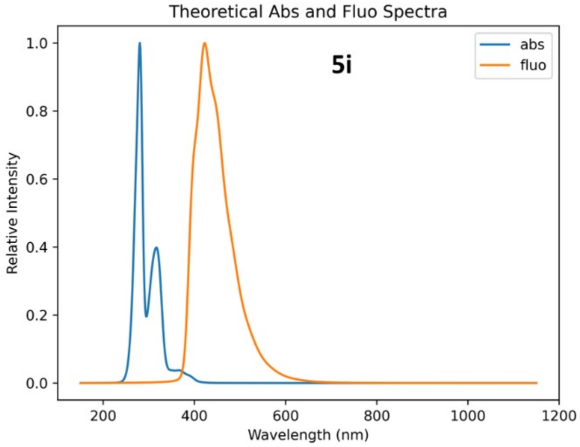

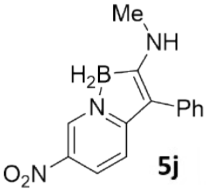

5j

	λmax (abs)	λmax (em)	HOMO-LUMO (eV)
computed	385 nm	471 nm	-

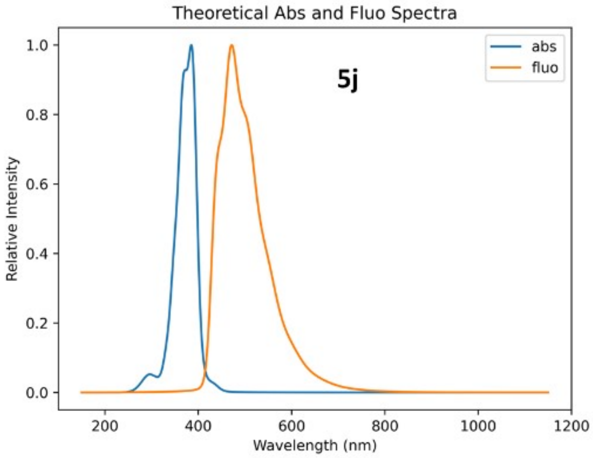

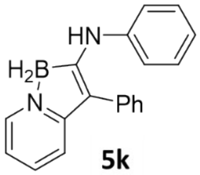

5k

	λmax (abs)	λmax (em)	HOMO-LUMO (eV)
computed	312 nm	476 nm	-

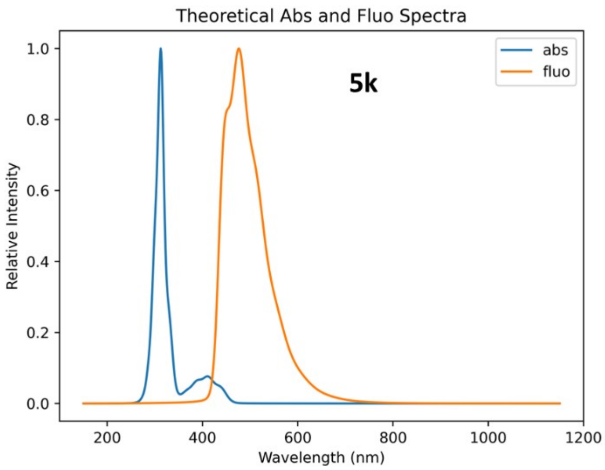

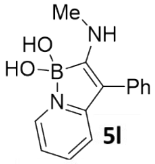

5l

	λmax (abs)	λmax (em)	HOMO-LUMO (eV)
computed	288 nm	521 nm	-

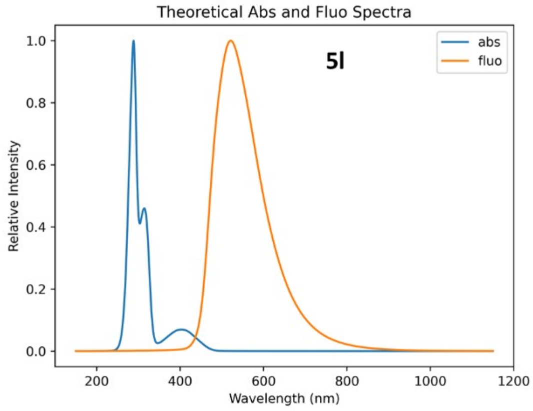

Crystallographic data

Crystallographic data of borindolizine 3a

Table 1. Crystal data and structure refinement for d2131_a.

Identification code	d2131_a	
Empirical formula	C11 H15 B N2 O2	
Formula weight	218.06	
Temperature	150(2) K	
Wavelength	1.54178 Å	
Crystal system	Monoclinic	
Space group	$P2_1/c$	
Unit cell dimensions	a = 11.3481(7) Å	α= 90°.
	b = 17.5922(11) Å	β= 100.544(4)°.
	c = 11.7050(7) Å	γ = 90°.
Volume	2297.3(2) $Å^3$	
Z	8	
Density (calculated)	1.261 Mg/m^3	
Absorption coefficient	0.694 mm^{-1}	
F(000)	928	
Crystal size	0.140 x 0.070 x 0.040 mm^3	
Theta range for data collection	3.962 to 66.182°.	
Index ranges	-13<=h<=13, -20<=k<=20, -13<=l<=13	
Reflections collected	49226	
Independent reflections	3950 [R(int) = 0.0721]	
Completeness to theta = 66.182°	98.5 %	
Absorption correction	Semi-empirical from equivalents	
Max. and min. transmission	0.7528 and 0.6287	
Refinement method	Full-matrix least-squares on F^2	
Data / restraints / parameters	3950 / 0 / 317	
Goodness-of-fit on F^2	1.047	
Final R indices [I>2sigma(I)]	R1 = 0.0379, wR2 = 0.0897	
R indices (all data)	R1 = 0.0491, wR2 = 0.0955	
Extinction coefficient	n/a	
Largest diff. peak and hole	0.226 and -0.189 e.$Å^{-3}$	

Table 3. Bond lengths [Å] and angles [°] for d2131_a.

O(1A)-C(10A)
O(2A)-C(10A)
O(2A)-C(11A)
N(1A)-C(7A)
N(1A)-C(3A)
N(1A)-B(1A)
N(2A)-C(1A)
N(2A)-C(8A)
N(2A)-H(2NA)
C(1A)-C(2A)
C(1A)-B(1A)
C(2A)-C(10A)
C(2A)-C(3A)
C(3A)-C(4A)
C(4A)-C(5A)
C(4A)-H(4AA)
C(5A)-C(6A)
C(5A)-H(5AA)
C(6A)-C(7A)
C(6A)-H(6AA)
C(7A)-H(7AA)
C(8A)-C(9A)
C(8A)-H(8AA)
C(8A)-H(8AB)
C(9A)-H(9AA)
C(9A)-H(9AB)
C(9A)-H(9AC)
C(11A)-H(11A)
C(11A)-H(11B)
C(11A)-H(11C)
B(1A)-H(1B1)
B(1A)-H(1B2)
O(1B)-C(10B)
O(2B)-C(10B)
O(2B)-C(11B)
N(1B)-C(7B)
N(1B)-C(3B)
N(1B)-B(1B)
N(2B)-C(1B)
N(2B)-C(8B)
N(2B)-H(2NB)
C(1B)-C(2B)
C(1B)-B(1B)
C(2B)-C(10B)
C(2B)-C(3B)
C(3B)-C(4B)
C(4B)-C(5B)
C(4B)-H(4BA)
C(5B)-C(6B)
C(5B)-H(5BA)

C(6B)-C(7B)
C(6B)-H(6BA)
C(7B)-H(7BA)
C(8B)-C(9B)
C(8B)-H(8BA)
C(8B)-H(8BB)
C(9B)-H(9BA)
C(9B)-H(9BB)
C(9B)-H(9BC)
C(11B)-H(11D)
C(11B)-H(11E)
C(11B)-H(11F)
B(1B)-H(1B3)
B(1B)-H(1B4)

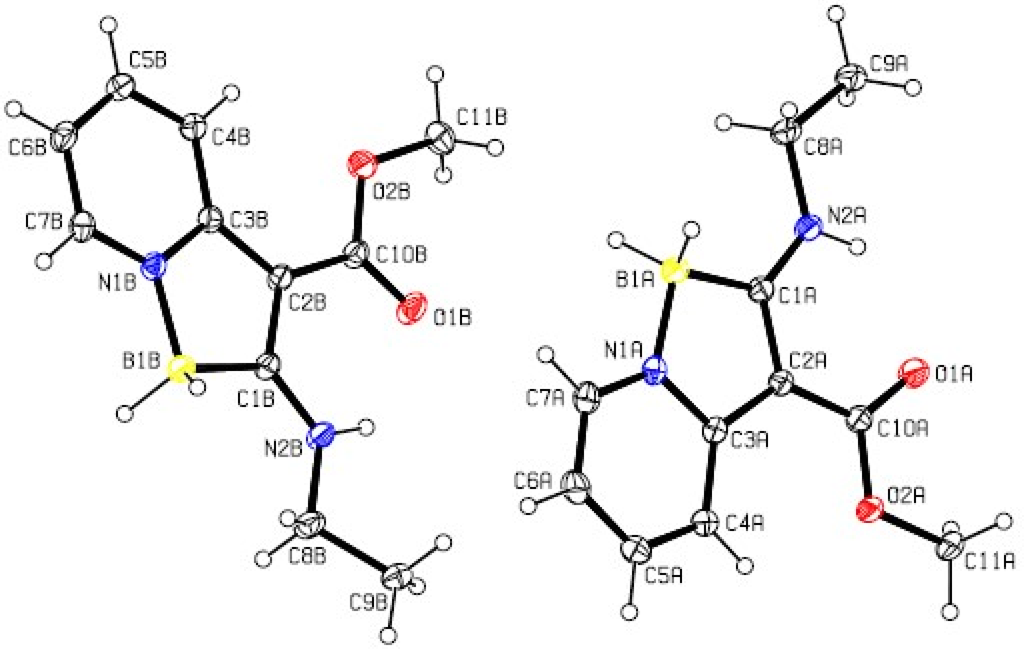

Crystallographic data of borindolizine 3p

Table 1. Crystal data and structure refinement for d2384_a.

Identification code	d2384_a	
Empirical formula	C19 H19 B N2	
Formula weight	286.17	
Temperature	150(2) K	
Wavelength	1.54178 Å	
Crystal system	Monoclinic	
Space group	C2/c	
Unit cell dimensions	a = 37.5497(19) Å	α= 90°.
	b = 10.0494(5) Å	β= 120.054(2)°.
	c = 19.2559(10) Å	γ = 90°.
Volume	6289.3(6) Å^3	
Z	16	

Density (calculated)	1.209 Mg/m^3
Absorption coefficient	0.536 mm^{-1}
F(000)	2432
Crystal size	0.290 x 0.120 x 0.100 mm^3
Theta range for data collection	2.719 to 65.859°.
Index ranges	-44<=h<=41, -11<=k<=11, -22<=l<=22
Reflections collected	82194
Independent reflections	5413 [R(int) = 0.0457]
Completeness to theta = 65.859°	99.1 %
Absorption correction	Semi-empirical from equivalents
Max. and min. transmission	0.7527 and 0.6642
Refinement method	Full-matrix least-squares on F^2
Data / restraints / parameters	5413 / 0 / 425
Goodness-of-fit on F^2	1.035
Final R indices [I>2sigma(I)]	R1 = 0.0392, wR2 = 0.1060
R indices (all data)	R1 = 0.0481, wR2 = 0.1136
Extinction coefficient	n/a
Largest diff. peak and hole	0.197 and -0.198 e.$Å^{-3}$

Table 2. Atomic coordinates (x 10^4) and equivalent isotropic displacement parameters ($Å^2$x 10^3) for d2384_a. U(eq) is defined as one third of the trace of the orthogonalized U^{ij} tensor.

	x	y	z	U(eq)
N(1A)	4254(1)	8332(1)	4506(1)	26(1)
N(2A)	3827(1)	6431(1)	5580(1)	31(1)
C(1A)	3918(1)	7196(1)	5114(1)	25(1)
C(2A)	4503(1)	8852(1)	4278(1)	28(1)
C(3A)	4373(1)	9840(1)	3686(1)	28(1)
C(4A)	4644(1)	10423(1)	3460(1)	34(1)
C(5A)	4509(1)	11427(1)	2910(1)	38(1)
C(6A)	4100(1)	11887(1)	2561(1)	36(1)
C(7A)	3832(1)	11344(1)	2764(1)	32(1)
C(8A)	3959(1)	10295(1)	3335(1)	27(1)
C(9A)	3699(1)	9711(1)	3575(1)	27(1)
C(10A)	3843(1)	8726(1)	4153(1)	25(1)
C(11A)	3641(1)	8023(1)	4509(1)	25(1)
C(12A)	4114(1)	5461(1)	6138(1)	38(1)
C(13A)	3206(1)	8245(1)	4270(1)	25(1)
C(14A)	3096(1)	8512(1)	4851(1)	26(1)
C(15A)	2690(1)	8716(1)	4644(1)	28(1)
C(16A)	2373(1)	8674(1)	3849(1)	28(1)
C(17A)	2479(1)	8418(1)	3264(1)	31(1)
C(18A)	2886(1)	8200(1)	3471(1)	29(1)
C(19A)	1935(1)	8891(2)	3644(1)	36(1)
B(1A)	4362(1)	7286(1)	5197(1)	28(1)
N(1B)	4252(1)	3344(1)	4508(1)	26(1)
N(2B)	3803(1)	1338(1)	5495(1)	32(1)
C(1B)	3898(1)	2132(1)	5049(1)	26(1)
C(2B)	4508(1)	3860(1)	4298(1)	27(1)
C(3B)	4388(1)	4850(1)	3710(1)	28(1)
C(4B)	4666(1)	5437(1)	3505(1)	33(1)
C(5B)	4537(1)	6434(1)	2951(1)	36(1)
C(6B)	4124(1)	6877(1)	2574(1)	35(1)
C(7B)	3849(1)	6332(1)	2758(1)	31(1)

C(8B)	3971(1)	5296(1)	3334(1)	26(1)
C(9B)	3703(1)	4707(1)	3553(1)	27(1)
C(10B)	3840(1)	3732(1)	4137(1)	24(1)
C(11B)	3626(1)	2987(1)	4454(1)	25(1)
C(12B)	4103(1)	482(1)	6121(1)	35(1)
C(13B)	3190(1)	3206(1)	4210(1)	26(1)
C(14B)	2926(1)	2139(1)	4096(1)	30(1)
C(15B)	2524(1)	2342(1)	3925(1)	33(1)
C(16B)	2362(1)	3617(1)	3835(1)	30(1)
C(17B)	2616(1)	4681(1)	3912(1)	29(1)
C(18B)	3021(1)	4486(1)	4103(1)	27(1)
C(19B)	1928(1)	3828(2)	3661(1)	40(1)
B(1B)	4350(1)	2271(1)	5180(1)	27(1)

Table 3. Bond lengths [Å] and angles [°] for d2384_a.

N(1A)-C(2A)	1.3214(16)
N(1A)-C(10A)	1.3952(15)
N(1A)-B(1A)	1.5817(18)
N(2A)-C(1A)	1.3493(16)
N(2A)-C(12A)	1.4507(17)
N(2A)-H(2NA)	0.932(16)
C(1A)-C(11A)	1.3845(17)
C(1A)-B(1A)	1.5977(18)
C(2A)-C(3A)	1.4017(18)
C(2A)-H(2AA)	0.9500
C(3A)-C(4A)	1.4174(18)
C(3A)-C(8A)	1.4248(18)
C(4A)-C(5A)	1.364(2)
C(4A)-H(4AA)	0.9500
C(5A)-C(6A)	1.411(2)
C(5A)-H(5AA)	0.9500
C(6A)-C(7A)	1.3647(19)
C(6A)-H(6AA)	0.9500
C(7A)-C(8A)	1.4212(18)
C(7A)-H(7AA)	0.9500
C(8A)-C(9A)	1.4024(17)
C(9A)-C(10A)	1.3818(18)
C(9A)-H(9AA)	0.9500
C(10A)-C(11A)	1.4369(17)
C(11A)-C(13A)	1.4765(17)
C(12A)-H(12A)	0.9800
C(12A)-H(12B)	0.9800
C(12A)-H(12C)	0.9800
C(13A)-C(14A)	1.3963(17)
C(13A)-C(18A)	1.4009(18)
C(14A)-C(15A)	1.3876(17)
C(14A)-H(14A)	0.9500
C(15A)-C(16A)	1.3912(18)
C(15A)-H(15A)	0.9500

C(16A)-C(17A)	1.3921(18)
C(16A)-C(19A)	1.5059(17)
C(17A)-C(18A)	1.3896(18)
C(17A)-H(17A)	0.9500
C(18A)-H(18A)	0.9500
C(19A)-H(19A)	0.9800
C(19A)-H(19B)	0.9800
C(19A)-H(19C)	0.9800
B(1A)-H(1A1)	1.142(15)
B(1A)-H(1A2)	1.119(15)
N(1B)-C(2B)	1.3214(16)
N(1B)-C(10B)	1.3952(15)
N(1B)-B(1B)	1.5794(17)
N(2B)-C(1B)	1.3456(16)
N(2B)-C(12B)	1.4503(17)
N(2B)-H(2NB)	0.923(17)
C(1B)-C(11B)	1.3864(17)
C(1B)-B(1B)	1.5936(18)
C(2B)-C(3B)	1.4017(18)
C(2B)-H(2BA)	0.9500
C(3B)-C(4B)	1.4176(17)
C(3B)-C(8B)	1.4267(17)
C(4B)-C(5B)	1.363(2)
C(4B)-H(4BA)	0.9500
C(5B)-C(6B)	1.414(2)
C(5B)-H(5BA)	0.9500
C(6B)-C(7B)	1.3637(19)
C(6B)-H(6BA)	0.9500
C(7B)-C(8B)	1.4192(18)
C(7B)-H(7BA)	0.9500
C(8B)-C(9B)	1.4017(17)
C(9B)-C(10B)	1.3829(17)
C(9B)-H(9BA)	0.9500
C(10B)-C(11B)	1.4392(17)
C(11B)-C(13B)	1.4770(17)
C(12B)-H(12D)	0.9800

C(12B)-H(12E)	0.9800
C(12B)-H(12F)	0.9800
C(13B)-C(14B)	1.4014(17)
C(13B)-C(18B)	1.4027(17)
C(14B)-C(15B)	1.3858(18)
C(14B)-H(14B)	0.9500
C(15B)-C(16B)	1.3919(18)
C(15B)-H(15B)	0.9500
C(16B)-C(17B)	1.3922(18)
C(16B)-C(19B)	1.5055(18)
C(17B)-C(18B)	1.3869(17)
C(17B)-H(17B)	0.9500
C(18B)-H(18B)	0.9500
C(19B)-H(19D)	0.9800
C(19B)-H(19E)	0.9800
C(19B)-H(19F)	0.9800
B(1B)-H(1B1)	1.142(15)
B(1B)-H(1B2)	1.122(16)

C(2A)-N(1A)-C(10A)	121.25(11)
C(2A)-N(1A)-B(1A)	127.96(10)
C(10A)-N(1A)-B(1A)	110.73(10)
C(1A)-N(2A)-C(12A)	122.29(11)
C(1A)-N(2A)-H(2NA)	116.7(10)
C(12A)-N(2A)-H(2NA)	118.8(10)
N(2A)-C(1A)-C(11A)	124.69(11)
N(2A)-C(1A)-B(1A)	124.38(11)
C(11A)-C(1A)-B(1A)	110.92(11)
N(1A)-C(2A)-C(3A)	122.31(11)
N(1A)-C(2A)-H(2AA)	118.8
C(3A)-C(2A)-H(2AA)	118.8
C(2A)-C(3A)-C(4A)	122.16(12)
C(2A)-C(3A)-C(8A)	117.75(11)
C(4A)-C(3A)-C(8A)	120.03(12)
C(5A)-C(4A)-C(3A)	120.04(13)
C(5A)-C(4A)-H(4AA)	120.0

C(3A)-C(4A)-H(4AA)	120.0
C(4A)-C(5A)-C(6A)	120.26(12)
C(4A)-C(5A)-H(5AA)	119.9
C(6A)-C(5A)-H(5AA)	119.9
C(7A)-C(6A)-C(5A)	121.15(13)
C(7A)-C(6A)-H(6AA)	119.4
C(5A)-C(6A)-H(6AA)	119.4
C(6A)-C(7A)-C(8A)	120.41(13)
C(6A)-C(7A)-H(7AA)	119.8
C(8A)-C(7A)-H(7AA)	119.8
C(9A)-C(8A)-C(7A)	122.99(12)
C(9A)-C(8A)-C(3A)	118.90(11)
C(7A)-C(8A)-C(3A)	118.11(11)
C(10A)-C(9A)-C(8A)	120.58(11)
C(10A)-C(9A)-H(9AA)	119.7
C(8A)-C(9A)-H(9AA)	119.7
C(9A)-C(10A)-N(1A)	119.16(11)
C(9A)-C(10A)-C(11A)	130.41(11)
N(1A)-C(10A)-C(11A)	110.37(10)
C(1A)-C(11A)-C(10A)	110.05(10)
C(1A)-C(11A)-C(13A)	126.87(11)
C(10A)-C(11A)-C(13A)	123.00(11)
N(2A)-C(12A)-H(12A)	109.5
N(2A)-C(12A)-H(12B)	109.5
H(12A)-C(12A)-H(12B)	109.5
N(2A)-C(12A)-H(12C)	109.5
H(12A)-C(12A)-H(12C)	109.5
H(12B)-C(12A)-H(12C)	109.5
C(14A)-C(13A)-C(18A)	116.87(11)
C(14A)-C(13A)-C(11A)	120.22(11)
C(18A)-C(13A)-C(11A)	122.92(11)
C(15A)-C(14A)-C(13A)	121.44(11)
C(15A)-C(14A)-H(14A)	119.3
C(13A)-C(14A)-H(14A)	119.3
C(14A)-C(15A)-C(16A)	121.43(12)
C(14A)-C(15A)-H(15A)	119.3

C(16A)-C(15A)-H(15A)	119.3
C(15A)-C(16A)-C(17A)	117.62(11)
C(15A)-C(16A)-C(19A)	120.16(12)
C(17A)-C(16A)-C(19A)	122.22(12)
C(18A)-C(17A)-C(16A)	121.04(12)
C(18A)-C(17A)-H(17A)	119.5
C(16A)-C(17A)-H(17A)	119.5
C(17A)-C(18A)-C(13A)	121.60(12)
C(17A)-C(18A)-H(18A)	119.2
C(13A)-C(18A)-H(18A)	119.2
C(16A)-C(19A)-H(19A)	109.5
C(16A)-C(19A)-H(19B)	109.5
H(19A)-C(19A)-H(19B)	109.5
C(16A)-C(19A)-H(19C)	109.5
H(19A)-C(19A)-H(19C)	109.5
H(19B)-C(19A)-H(19C)	109.5
N(1A)-B(1A)-C(1A)	97.89(9)
N(1A)-B(1A)-H(1A1)	111.3(7)
C(1A)-B(1A)-H(1A1)	113.1(7)
N(1A)-B(1A)-H(1A2)	109.7(8)
C(1A)-B(1A)-H(1A2)	113.7(7)
H(1A1)-B(1A)-H(1A2)	110.6(11)
C(2B)-N(1B)-C(10B)	121.65(10)
C(2B)-N(1B)-B(1B)	127.64(10)
C(10B)-N(1B)-B(1B)	110.71(9)
C(1B)-N(2B)-C(12B)	122.56(11)
C(1B)-N(2B)-H(2NB)	119.2(10)
C(12B)-N(2B)-H(2NB)	118.2(10)
N(2B)-C(1B)-C(11B)	125.05(11)
N(2B)-C(1B)-B(1B)	123.61(11)
C(11B)-C(1B)-B(1B)	111.24(10)
N(1B)-C(2B)-C(3B)	122.31(11)
N(1B)-C(2B)-H(2BA)	118.8
C(3B)-C(2B)-H(2BA)	118.8
C(2B)-C(3B)-C(4B)	122.36(12)
C(2B)-C(3B)-C(8B)	117.52(11)

C(4B)-C(3B)-C(8B)	120.08(12)
C(5B)-C(4B)-C(3B)	120.07(12)
C(5B)-C(4B)-H(4BA)	120.0
C(3B)-C(4B)-H(4BA)	120.0
C(4B)-C(5B)-C(6B)	120.15(12)
C(4B)-C(5B)-H(5BA)	119.9
C(6B)-C(5B)-H(5BA)	119.9
C(7B)-C(6B)-C(5B)	121.12(13)
C(7B)-C(6B)-H(6BA)	119.4
C(5B)-C(6B)-H(6BA)	119.4
C(6B)-C(7B)-C(8B)	120.57(12)
C(6B)-C(7B)-H(7BA)	119.7
C(8B)-C(7B)-H(7BA)	119.7
C(9B)-C(8B)-C(7B)	123.04(11)
C(9B)-C(8B)-C(3B)	118.96(11)
C(7B)-C(8B)-C(3B)	118.00(11)
C(10B)-C(9B)-C(8B)	120.87(11)
C(10B)-C(9B)-H(9BA)	119.6
C(8B)-C(9B)-H(9BA)	119.6
C(9B)-C(10B)-N(1B)	118.65(11)
C(9B)-C(10B)-C(11B)	130.84(11)
N(1B)-C(10B)-C(11B)	110.51(10)
C(1B)-C(11B)-C(10B)	109.57(10)
C(1B)-C(11B)-C(13B)	126.58(11)
C(10B)-C(11B)-C(13B)	123.68(11)
N(2B)-C(12B)-H(12D)	109.5
N(2B)-C(12B)-H(12E)	109.5
H(12D)-C(12B)-H(12E)	109.5
N(2B)-C(12B)-H(12F)	109.5
H(12D)-C(12B)-H(12F)	109.5
H(12E)-C(12B)-H(12F)	109.5
C(14B)-C(13B)-C(18B)	116.49(11)
C(14B)-C(13B)-C(11B)	121.41(11)
C(18B)-C(13B)-C(11B)	122.07(11)
C(15B)-C(14B)-C(13B)	121.61(12)
C(15B)-C(14B)-H(14B)	119.2

C(13B)-C(14B)-H(14B)	119.2
C(14B)-C(15B)-C(16B)	121.50(12)
C(14B)-C(15B)-H(15B)	119.3
C(16B)-C(15B)-H(15B)	119.3
C(15B)-C(16B)-C(17B)	117.29(11)
C(15B)-C(16B)-C(19B)	121.10(12)
C(17B)-C(16B)-C(19B)	121.62(12)
C(18B)-C(17B)-C(16B)	121.46(11)
C(18B)-C(17B)-H(17B)	119.3
C(16B)-C(17B)-H(17B)	119.3
C(17B)-C(18B)-C(13B)	121.56(11)
C(17B)-C(18B)-H(18B)	119.2
C(13B)-C(18B)-H(18B)	119.2
C(16B)-C(19B)-H(19D)	109.5
C(16B)-C(19B)-H(19E)	109.5
H(19D)-C(19B)-H(19E)	109.5
C(16B)-C(19B)-H(19F)	109.5
H(19D)-C(19B)-H(19F)	109.5
H(19E)-C(19B)-H(19F)	109.5
N(1B)-B(1B)-C(1B)	97.89(10)
N(1B)-B(1B)-H(1B1)	111.3(7)
C(1B)-B(1B)-H(1B1)	114.9(7)
N(1B)-B(1B)-H(1B2)	109.9(8)
C(1B)-B(1B)-H(1B2)	112.0(7)
H(1B1)-B(1B)-H(1B2)	110.2(11)

Symmetry transformations used to generate equivalent atoms:

Table 4. Anisotropic displacement parameters ($Å^2$x 10^3) for d2384_a. The anisotropic displacement factor exponent takes the form: $-2\pi^2[h^2 a^{*2}U^{11} + ... + 2 h k a^* b^* U^{12}]$

	U^{11}	U^{22}	U^{33}	U^{23}	U^{13}	U^{12}
N(1A)	25(1)	24(1)	29(1)	-3(1)	14(1)	0(1)
N(2A)	32(1)	28(1)	31(1)	5(1)	16(1)	2(1)
C(1A)	29(1)	21(1)	25(1)	-4(1)	13(1)	0(1)
C(2A)	26(1)	29(1)	31(1)	-5(1)	15(1)	-1(1)
C(3A)	30(1)	27(1)	29(1)	-6(1)	16(1)	-4(1)
C(4A)	31(1)	38(1)	36(1)	-6(1)	18(1)	-7(1)
C(5A)	40(1)	40(1)	37(1)	-4(1)	23(1)	-13(1)
C(6A)	46(1)	31(1)	32(1)	-1(1)	20(1)	-8(1)
C(7A)	35(1)	28(1)	31(1)	-1(1)	16(1)	-2(1)
C(8A)	31(1)	24(1)	27(1)	-5(1)	15(1)	-3(1)
C(9A)	26(1)	27(1)	30(1)	0(1)	15(1)	2(1)
C(10A)	25(1)	23(1)	27(1)	-3(1)	13(1)	1(1)
C(11A)	27(1)	22(1)	27(1)	-2(1)	14(1)	0(1)
C(12A)	47(1)	31(1)	33(1)	6(1)	19(1)	8(1)
C(13A)	28(1)	18(1)	30(1)	2(1)	15(1)	1(1)
C(14A)	27(1)	24(1)	26(1)	0(1)	12(1)	-1(1)
C(15A)	30(1)	26(1)	32(1)	-1(1)	19(1)	-2(1)
C(16A)	27(1)	22(1)	35(1)	3(1)	16(1)	-1(1)
C(17A)	28(1)	31(1)	27(1)	2(1)	10(1)	0(1)
C(18A)	32(1)	29(1)	28(1)	1(1)	16(1)	1(1)
C(19A)	28(1)	40(1)	40(1)	5(1)	16(1)	1(1)
B(1A)	27(1)	25(1)	29(1)	0(1)	12(1)	2(1)
N(1B)	24(1)	24(1)	29(1)	-3(1)	14(1)	0(1)
N(2B)	31(1)	31(1)	36(1)	7(1)	17(1)	2(1)
C(1B)	29(1)	21(1)	28(1)	-3(1)	14(1)	0(1)
C(2B)	24(1)	27(1)	31(1)	-4(1)	14(1)	-1(1)
C(3B)	30(1)	26(1)	28(1)	-6(1)	15(1)	-4(1)
C(4B)	30(1)	36(1)	34(1)	-5(1)	17(1)	-6(1)
C(5B)	39(1)	39(1)	36(1)	-4(1)	22(1)	-12(1)
C(6B)	44(1)	30(1)	32(1)	0(1)	19(1)	-7(1)
C(7B)	33(1)	28(1)	30(1)	0(1)	14(1)	-2(1)

C(8B)	29(1)	24(1)	26(1)	-5(1)	13(1)	-2(1)
C(9B)	24(1)	27(1)	28(1)	-2(1)	13(1)	0(1)
C(10B)	24(1)	22(1)	27(1)	-4(1)	12(1)	0(1)
C(11B)	26(1)	22(1)	28(1)	-2(1)	13(1)	0(1)
C(12B)	41(1)	29(1)	33(1)	5(1)	16(1)	3(1)
C(13B)	27(1)	25(1)	26(1)	-1(1)	14(1)	0(1)
C(14B)	30(1)	23(1)	38(1)	0(1)	17(1)	1(1)
C(15B)	28(1)	29(1)	41(1)	-2(1)	17(1)	-4(1)
C(16B)	26(1)	32(1)	30(1)	-3(1)	13(1)	1(1)
C(17B)	31(1)	26(1)	30(1)	-1(1)	15(1)	4(1)
C(18B)	29(1)	24(1)	30(1)	-2(1)	16(1)	-1(1)
C(19B)	28(1)	44(1)	46(1)	-7(1)	17(1)	1(1)
B(1B)	26(1)	25(1)	29(1)	1(1)	12(1)	2(1)

Table 5. Hydrogen coordinates (x 10^4) and isotropic displacement parameters ($Å^2$x 10^3) for d2384_a.

	x	y	z	U(eq)
H(2NA)	3551(5)	6371(15)	5434(9)	41(4)
H(2AA)	4779	8545	4524	34
H(4AA)	4919	10113	3692	41
H(5AA)	4692	11818	2761	45
H(6AA)	4010	12587	2179	43
H(7AA)	3558	11670	2523	38
H(9AA)	3421	9995	3338	33
H(12A)	4007	5108	6472	56
H(12B)	4381	5887	6480	56
H(12C)	4148	4731	5838	56
H(14A)	3305	8554	5399	32
H(15A)	2626	8889	5054	34
H(17A)	2270	8391	2716	37
H(18A)	2949	8018	3060	35
H(19A)	1764	9074	3069	55
H(19B)	1922	9649	3951	55
H(19C)	1833	8091	3780	55
H(1A1)	4469(4)	6300(15)	5067(9)	38(4)
H(1A2)	4604(4)	7704(15)	5784(9)	39(4)
H(2NB)	3535(5)	1309(16)	5390(10)	45(4)
H(2BA)	4784	3548	4556	33
H(4BA)	4943	5136	3754	39
H(5BA)	4724	6831	2817	44
H(6BA)	4037	7568	2185	42
H(7BA)	3574	6647	2499	37
H(9BA)	3424	4982	3296	32
H(12D)	3986	116	6435	53
H(12E)	4350	999	6473	53
H(12F)	4176	-248	5878	53
H(14B)	3024	1255	4138	37

H(15B)	2357	1595	3868	39
H(17B)	2510	5561	3833	35
H(18B)	3188	5237	4162	33
H(19D)	1911	4670	3899	60
H(19E)	1848	3094	3890	60
H(19F)	1741	3856	3079	60
H(1B1)	4481(4)	1321(15)	5068(9)	38(4)
H(1B2)	4572(4)	2692(15)	5785(9)	41(4)

Table 6. Torsion angles [°] for d2384_a.

C(12A)-N(2A)-C(1A)-C(11A)	172.21(12)
C(12A)-N(2A)-C(1A)-B(1A)	-8.99(19)
C(10A)-N(1A)-C(2A)-C(3A)	1.36(18)
B(1A)-N(1A)-C(2A)-C(3A)	-175.82(11)
N(1A)-C(2A)-C(3A)-C(4A)	178.12(12)
N(1A)-C(2A)-C(3A)-C(8A)	0.79(18)
C(2A)-C(3A)-C(4A)-C(5A)	-176.76(12)
C(8A)-C(3A)-C(4A)-C(5A)	0.51(19)
C(3A)-C(4A)-C(5A)-C(6A)	-0.3(2)
C(4A)-C(5A)-C(6A)-C(7A)	0.0(2)
C(5A)-C(6A)-C(7A)-C(8A)	-0.1(2)
C(6A)-C(7A)-C(8A)-C(9A)	179.10(12)
C(6A)-C(7A)-C(8A)-C(3A)	0.32(18)
C(2A)-C(3A)-C(8A)-C(9A)	-1.98(17)
C(4A)-C(3A)-C(8A)-C(9A)	-179.37(11)
C(2A)-C(3A)-C(8A)-C(7A)	176.85(11)
C(4A)-C(3A)-C(8A)-C(7A)	-0.53(18)
C(7A)-C(8A)-C(9A)-C(10A)	-177.68(11)
C(3A)-C(8A)-C(9A)-C(10A)	1.09(18)
C(8A)-C(9A)-C(10A)-N(1A)	1.01(18)
C(8A)-C(9A)-C(10A)-C(11A)	177.75(12)
C(2A)-N(1A)-C(10A)-C(9A)	-2.28(17)
B(1A)-N(1A)-C(10A)-C(9A)	175.35(11)
C(2A)-N(1A)-C(10A)-C(11A)	-179.63(10)
B(1A)-N(1A)-C(10A)-C(11A)	-2.00(13)
N(2A)-C(1A)-C(11A)-C(10A)	177.90(11)
B(1A)-C(1A)-C(11A)-C(10A)	-1.04(14)
N(2A)-C(1A)-C(11A)-C(13A)	1.1(2)
B(1A)-C(1A)-C(11A)-C(13A)	-177.88(11)
C(9A)-C(10A)-C(11A)-C(1A)	-175.04(12)
N(1A)-C(10A)-C(11A)-C(1A)	1.92(14)
C(9A)-C(10A)-C(11A)-C(13A)	1.9(2)
N(1A)-C(10A)-C(11A)-C(13A)	178.90(10)
C(1A)-C(11A)-C(13A)-C(14A)	48.04(18)

C(10A)-C(11A)-C(13A)-C(14A)	-128.42(13)
C(1A)-C(11A)-C(13A)-C(18A)	-131.91(13)
C(10A)-C(11A)-C(13A)-C(18A)	51.63(17)
C(18A)-C(13A)-C(14A)-C(15A)	0.32(17)
C(11A)-C(13A)-C(14A)-C(15A)	-179.63(11)
C(13A)-C(14A)-C(15A)-C(16A)	-0.46(18)
C(14A)-C(15A)-C(16A)-C(17A)	-0.01(18)
C(14A)-C(15A)-C(16A)-C(19A)	179.61(11)
C(15A)-C(16A)-C(17A)-C(18A)	0.60(18)
C(19A)-C(16A)-C(17A)-C(18A)	-179.01(12)
C(16A)-C(17A)-C(18A)-C(13A)	-0.74(19)
C(14A)-C(13A)-C(18A)-C(17A)	0.27(18)
C(11A)-C(13A)-C(18A)-C(17A)	-179.78(11)
C(2A)-N(1A)-B(1A)-C(1A)	178.68(11)
C(10A)-N(1A)-B(1A)-C(1A)	1.26(12)
N(2A)-C(1A)-B(1A)-N(1A)	-179.05(11)
C(11A)-C(1A)-B(1A)-N(1A)	-0.11(13)
C(12B)-N(2B)-C(1B)-C(11B)	-178.75(12)
C(12B)-N(2B)-C(1B)-B(1B)	-2.75(19)
C(10B)-N(1B)-C(2B)-C(3B)	1.82(18)
B(1B)-N(1B)-C(2B)-C(3B)	-177.42(11)
N(1B)-C(2B)-C(3B)-C(4B)	177.72(11)
N(1B)-C(2B)-C(3B)-C(8B)	-0.10(18)
C(2B)-C(3B)-C(4B)-C(5B)	-177.44(12)
C(8B)-C(3B)-C(4B)-C(5B)	0.33(19)
C(3B)-C(4B)-C(5B)-C(6B)	-0.5(2)
C(4B)-C(5B)-C(6B)-C(7B)	0.5(2)
C(5B)-C(6B)-C(7B)-C(8B)	-0.2(2)
C(6B)-C(7B)-C(8B)-C(9B)	179.33(12)
C(6B)-C(7B)-C(8B)-C(3B)	0.06(18)
C(2B)-C(3B)-C(8B)-C(9B)	-1.53(17)
C(4B)-C(3B)-C(8B)-C(9B)	-179.40(11)
C(2B)-C(3B)-C(8B)-C(7B)	177.78(11)
C(4B)-C(3B)-C(8B)-C(7B)	-0.09(17)
C(7B)-C(8B)-C(9B)-C(10B)	-177.77(11)
C(3B)-C(8B)-C(9B)-C(10B)	1.50(18)

C(8B)-C(9B)-C(10B)-N(1B)	0.15(17)
C(8B)-C(9B)-C(10B)-C(11B)	-179.45(12)
C(2B)-N(1B)-C(10B)-C(9B)	-1.84(17)
B(1B)-N(1B)-C(10B)-C(9B)	177.51(11)
C(2B)-N(1B)-C(10B)-C(11B)	177.84(10)
B(1B)-N(1B)-C(10B)-C(11B)	-2.81(13)
N(2B)-C(1B)-C(11B)-C(10B)	176.72(11)
B(1B)-C(1B)-C(11B)-C(10B)	0.29(14)
N(2B)-C(1B)-C(11B)-C(13B)	1.2(2)
B(1B)-C(1B)-C(11B)-C(13B)	-175.22(11)
C(9B)-C(10B)-C(11B)-C(1B)	-178.79(12)
N(1B)-C(10B)-C(11B)-C(1B)	1.58(14)
C(9B)-C(10B)-C(11B)-C(13B)	-3.1(2)
N(1B)-C(10B)-C(11B)-C(13B)	177.25(11)
C(1B)-C(11B)-C(13B)-C(14B)	-44.96(18)
C(10B)-C(11B)-C(13B)-C(14B)	140.14(13)
C(1B)-C(11B)-C(13B)-C(18B)	133.09(13)
C(10B)-C(11B)-C(13B)-C(18B)	-41.81(18)
C(18B)-C(13B)-C(14B)-C(15B)	-2.96(18)
C(11B)-C(13B)-C(14B)-C(15B)	175.19(12)
C(13B)-C(14B)-C(15B)-C(16B)	1.8(2)
C(14B)-C(15B)-C(16B)-C(17B)	0.94(19)
C(14B)-C(15B)-C(16B)-C(19B)	-179.13(13)
C(15B)-C(16B)-C(17B)-C(18B)	-2.51(18)
C(19B)-C(16B)-C(17B)-C(18B)	177.56(12)
C(16B)-C(17B)-C(18B)-C(13B)	1.35(19)
C(14B)-C(13B)-C(18B)-C(17B)	1.39(18)
C(11B)-C(13B)-C(18B)-C(17B)	-176.75(11)
C(2B)-N(1B)-B(1B)-C(1B)	-178.02(11)
C(10B)-N(1B)-B(1B)-C(1B)	2.68(12)
N(2B)-C(1B)-B(1B)-N(1B)	-178.23(11)
C(11B)-C(1B)-B(1B)-N(1B)	-1.74(13)

Symmetry transformations used to generate equivalent atoms:

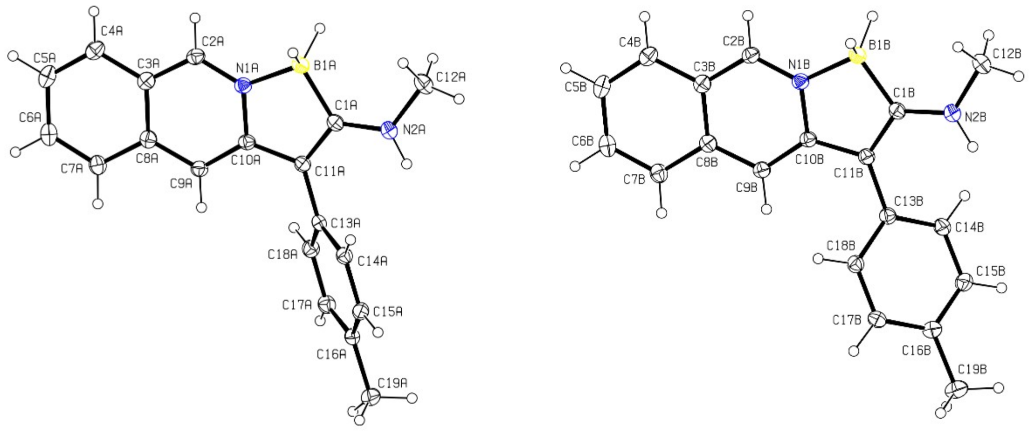

References

(1) Jiang, X.; Boehm, P.; Hartwig, J. F. Stereodivergent Allylation of Azaaryl Acetamides and Acetates by Synergistic Iridium and Copper Catalysis. *J. Am. Chem. Soc.* **2018**, *140* (4), 1239–1242. https://doi.org/10.1021/jacs.7b12824.

(2) Abrunhosa, I.; Gulea, M.; Masson, S. Efficient New Protocol to Synthesize Aromatic and Heteroaromatic Dithioesters. *Synthesis (Stuttg).* **2004**, *2004* (6), 928–934. https://doi.org/10.1055/s-2004-822311.

(3) Ramazani, A.; Zeinali Nasrabadi, F.; Ahmadi, Y. One-Pot, Four-Component Synthesis of Fully Substituted 1,3,4-Oxadiazole Derivatives from (Isocyanoimino)Triphenylphosphorane, a Primary Amine, an Aromatic Carboxylic Acid, and Chloroacetone. *Helv. Chim. Acta.* **2011**, *94* (6), 1024–1029. https://doi.org/10.1002/hlca.201000356.

(4) Dong, C.; Wang, X.; Pei, Z.; Shen, R. Metal-Free Denitrogenative C-C Couplings of Pyridotriazoles with Boronic Acids to Afford α-Secondary and α-Tertiary Pyridines. *Org. Lett.* **2019**. https://doi.org/10.1021/acs.orglett.9b01334.

(5) Lebdev, Y.; Apte, C.; Cheng, S.; Lavigne, C.; Lough, A.; Aspuru-Guzik, A.; Seferos, D.S.; Yudin, A.K. Boramidine: A Versatile Structural Motif for the Design of Fluorescent Heterocycles. *J. Am. Chem. Soc.* **2020,** 142 (31), 13544-13549. https://doi.org/10.1021/jacs.0c05410

(6) Bannwarth, C.; Ehlert, S.; Grimme, S. GFN2-XTB—An Accurate and Broadly Parametrized Self-Consistent Tight-Binding Quantum Chemical Method with Multipole Electrostatics and Density-Dependent Dispersion Contributions. *J. Chem. Theory Comput.* **2019**, *15* (3), 1652–1671. https://doi.org/10.1021/acs.jctc.8b01176.

(7) Grimme, S. Exploration of Chemical Compound, Conformer, and Reaction Space with Meta-Dynamics Simulations Based on Tight-Binding Quantum Chemical Calculations. *J. Chem. Theory Comput.* **2019**, *15* (5), 2847–2862. https://doi.org/10.1021/acs.jctc.9b00143.

(8) Humeniuk, A.; Bužančić, M.; Hoche, J.; Cerezo, J.; Mitrić, R.; Santoro, F.; Bonačić-Koutecký, V. Predicting Fluorescence Quantum Yields for Molecules in Solution: A Critical Assessment of the

Harmonic Approximation and the Choice of the Lineshape Function. *J. Chem. Phys.* **2020**, *152* (5), 054107. https://doi.org/10.1063/1.5143212.

(9) Laurent, A. D.; Adamo, C.; Jacquemin, D. Dye Chemistry with Time-Dependent Density Functional Theory. *Phys. Chem. Chem. Phys.* **2014**, *16* (28), 14334–14356. https://doi.org/10.1039/C3CP55336A.

(10) Batra, K.; Zahn, S.; Heine, T. Benchmark of Simplified Time-Dependent Density Functional Theory for UV–Vis Spectral Properties of Porphyrinoids. *Adv. Theory Simulations* **2020**, *3* (1), 1900192. https://doi.org/10.1002/adts.201900192.

(11) Charaf-Eddin, A.; Planchat, A.; Mennucci, B.; Adamo, C.; Jacquemin, D. Choosing a Functional for Computing Absorption and Fluorescence Band Shapes with TD-DFT. *J. Chem. Theory Comput.* **2013**, *9* (6), 2749–2760. https://doi.org/10.1021/ct4000795.

(12) Petrenko, T.; Neese, F. Analysis and Prediction of Absorption Band Shapes, Fluorescence Band Shapes, Resonance Raman Intensities, and Excitation Profiles Using the Time-Dependent Theory of Electronic Spectroscopy. *J. Chem. Phys.* **2007**, *127* (16), 164319. https://doi.org/10.1063/1.2770706.

(13) Shao, Y.; Gan, Z.; Epifanovsky, E.; Gilbert, A. T. B.; Wormit, M.; Kussmann, J.; Lange, A. W.; Behn, A.; Deng, J.; Feng, X.; et al. Advances in Molecular Quantum Chemistry Contained in the Q-Chem 4 Program Package. *Mol. Phys.* **2015**, *113* (2), 184–215. https://doi.org/10.1080/00268976.2014.952696.

(14) de Souza, B.; Neese, F.; Izsák, R. On the Theoretical Prediction of Fluorescence Rates from First Principles Using the Path Integral Approach. *J. Chem. Phys.* **2018**, *148* (3), 034104. https://doi.org/10.1063/1.5010895.
